# Prehabilitation in older patients prior to elective cardiac procedures (PRECOVERY): study protocol of a multicenter randomized controlled trial

**DOI:** 10.1186/s13063-023-07511-w

**Published:** 2023-08-15

**Authors:** Carolin Steinmetz, Stephanie Heinemann, Ingo Kutschka, Gerd Hasenfuß, Thomas Asendorf, Bjoern Andrew Remppis, Ernst Knoglinger, Clemens Grefe, Johannes Maximilian Albes, Hassina Baraki, Christian Baumbach, Susanne Brunner, Susann Ernst, Wolfgang Harringer, Dirk Heider, Daniela Heidkamp, Christoph Herrmann-Lingen, Eva Hummers, Thomas Kocar, Hans-Helmut König, Simone Krieger, Andreas Liebold, Andreas Martens, Marcus Matzeder, Friedrich Mellert, Christiane Müller, Miriam Puls, Nils Reiss, Martin Schikora, Thomas Schmidt, Martin Vestweber, Monika Sadlonova, Christine A. F. von Arnim, PRECOVERY investigators, Michael Denkinger, Oliver Dewald, Michael Don, Julia Frankenhauser-Manuß, Christine Kleber-Peukert, Anna-Maria Kloidt, Tim Matthes, Annemieke Munderloh, Elisabeth Schieffer

**Affiliations:** 1 Department of Geriatrics, University of Goettingen Medical Center, Robert-Koch-Straße 40, 37075 Goettingen, Germany; 2 Department of Cardiovascular and Thoracic Surgery, University of Goettingen Medical Center, Goettingen, Germany; 3 German Center for Cardiovascular Research (DZHK), Partner Site Goettingen, Goettingen, Germany; 4 Department of Cardiology and Pneumology, University of Goettingen Medical Center, Goettingen, Germany; 5 Department of Medical Statistics, University of Goettingen Medical Center, Goettingen, Germany; 6 Heart and Vascular Center Bad Bevensen, Bad Bevensen, Germany; 7 Kirchberg Clinic, Bad Lauterberg, Germany; 8 Clinic and Rehabilitation Center Lippoldsberg, Wesertal, Germany; 9 Immanuel Clinic Bernau, Brandenburg Heart Center, Bernau, Germany; 10 Clinic Fallingbostel, Bad Fallingbostel, Germany; 11 ZAR Center for Outpatient Rehabilitation GmbH, Ulm, Germany; 12 Department of Cardiac, Thoracic and Vascular Surgery, Braunschweig Municipal Hospital, Brunswick, Germany; 13 Department of Health Economics and Health Services Research, University Medical Center Hamburg-Eppendorf, Hamburg-Eppendorf, Germany; 14 Rehabilitation Center Oldenburg, Oldenburg, Germany; 15 Department of Psychosomatic Medicine and Psychotherapy, University of Goettingen Medical Center, Goettingen, Germany; 16 Department of General Practice, University of Goettingen Medical Center, Goettingen, Germany; 17 Geriatric Center Ulm, Ulm, Germany; 18 Department for Thoracic, Cardiac and Vascular Surgery, Ulm University Medical Center, Ulm, Germany; 19 Department of Cardiothoracic, Transplantation and Vascular Surgery, Hannover Medical School, Hannover, Germany; 20 AOK Health Insurance (AOK Lower Saxony), Helmstedt, Germany; 21 Department of Cardiac Surgery, Oldenburg Hospital, Oldenburg, Germany; 22 Schüchtermann-Schiller’sche Clinic, Bad Rothenfelde, Germany; 23 Brandenburg Clinic, Bernau Waldsiedlung, Germany; 24 Institute of Cardiology and Sports Medicine, Department Preventive and Rehabilitative Sport and Exercise Medicine, German Sport University, Cologne, Germany; 25 German Heart Foundation, Frankfurt am Main, Germany

**Keywords:** Prehabilitation, Preoperative preparation, Cardiac procedure, Older patients, Preoperative exercise, Preoperative preparation, Randomized controlled trial

## Abstract

**Background:**

Previous studies have demonstrated the efficacy of rehabilitation after a cardiovascular procedure. Especially older and multimorbid patients benefit from rehabilitation after a cardiac procedure. Prehabilitation prior to cardiac procedures may also have positive effects on patients’ pre- and postoperative outcomes. Results of a current meta-analysis show that prehabilitation prior to cardiac procedures can improve perioperative outcomes and alleviate adverse effects. Germany currently lacks a structured cardiac prehabilitation program for older patients, which is coordinated across healthcare sectors.

**Methods:**

In a randomized, controlled, two-arm parallel group, assessor-blinded multicenter intervention trial (PRECOVERY), we will randomize 422 patients aged 75 years or older scheduled for an elective cardiac procedure (e.g., coronary artery bypass graft surgery or transcatheter aortic valve replacement). In PRECOVERY, patients randomized to the intervention group participate in a 2-week multimodal prehabilitation intervention conducted in selected cardiac-specific rehabilitation facilities. The multimodal prehabilitation includes seven modules: exercise therapy, occupational therapy, cognitive training, psychosocial intervention, disease-specific education, education with relatives, and nutritional intervention. Participants in the control group receive standard medical care. The co-primary outcomes are quality of life (QoL) and mortality after 12 months. QoL will be measured by the EuroQol 5-dimensional questionnaire (EQ-5D-5L). A health economic evaluation using health insurance data will measure cost-effectiveness. A mixed-methods process evaluation will accompany the randomized, controlled trial to evaluate dose, reach, fidelity and adaptions of the intervention.

**Discussion:**

In this study, we investigate whether a tailored prehabilitation program can improve long-term survival, QoL and functional capacity. Additionally, we will analyze whether the intervention is cost-effective. This is the largest cardiac prehabilitation trial targeting the wide implementation of a new form of care for geriatric cardiac patients.

**Trial registration:**

German Clinical Trials Register (DRKS; http://www.drks.de; DRKS00030526). Registered on 30 January 2023.

**Supplementary Information:**

The online version contains supplementary material available at 10.1186/s13063-023-07511-w.

## Administrative information

**Table:** 

Title {1}	Prehabilitation in older patients prior to elective cardiac procedure (PRECOVERY): study protocol for a randomized, controlled, longitudinal, multicenter, two-arm, assessor-blinded trial
Trial registration {2a and 2b}	German Clinical Trials Register (DRKS; http://www.drks.de) (DRKS00030526)
Protocol version {3}	V3.0 from 27^th^ of March 2023
Funding {4}	Grant 01NVF21109 of the “Innovationsausschuss” of the German Federal Joint Committee (G-BA)
Author details {5a}	Carolin Steinmetz, PhD^1†^, Stephanie Heinemann, PhD^1†^, Ingo Kutschka, MD^2,3^, Gerd Hasenfuß, MD^3,4^, Thomas Asendorf, PhD^5^, Bjoern Andrew Remppis, MD^6^, Ernst Knoglinger, MD^7^, Clemens Grefe, MD^8^, Johannes Maximilian Albes, MD^9^, Hassina Baraki, MD^2,3^, Christian Baumbach, MD^6^, Susanne Brunner, MD^10^, Susann Ernst, MD^11^, Wolfgang Harringer, MD^12^, Dirk Heider, PhD^13^, Daniela Heidkamp, MD^14^, Christoph Herrmann-Lingen, MD^3,15^, Eva Hummers, MD^16^, Thomas Kocar, MD^17^, Hans-Helmut König, PhD^13^, Simone Krieger PhD^15^, Andreas Liebold, MD^18^, Andreas Martens, MD^19^, Marcus Matzeder^20^, Friedrich Mellert, MD^21^, Christiane Müller, MD^16^, Miriam Puls, MD^3,4^, Nils Reiss, MD^22^, Martin Schikora, MD^23^, Thomas Schmidt, PhD^24^, Martin Vestweber^25^, Monika Sadlonova, MD^1,2,3,15†^, Christine A.F. von Arnim^1,3†*^ and other associated PRECOVERY investigators as well as PRECOVERY investigators ^1^Department of Geriatrics, University of Goettingen Medical Center, Goettingen, Germany ^2^Department of Cardiovascular and Thoracic Surgery, University of Goettingen Medical Center, Goettingen, Germany ^3^German Center for Cardiovascular Research (DZHK), partner site Goettingen, Goettingen, Germany ^4^Department of Cardiology and Pneumology, University of Goettingen Medical Center, Goettingen, Germany ^5^Department of Medical Statistics, University of Goettingen Medical Center, Goettingen, Germany ^6^Heart and Vascular Center Bad Bevensen, Bad Bevensen, Germany ^7^Kirchberg Clinic, Bad Lauterberg, Bad Lauterberg, Germany ^8^Clinic and Rehabilitation Center Lippoldsberg, Wesertal, Germany ^9^Immanuel Clinic Bernau, Brandenburg Heart Center, Bernau, Germany ^10^Clinic Fallingbostel, Bad Fallingbostel, Germany ^11^ZAR Center for Outpatient Rehabilitation GmbH, Ulm, Germany ^12^Department of Cardiac, Thoracic and Vascular Surgery, Braunschweig Municipal Hospital, Braunschweig, Germany ^13^Department of Health Economics & Health Services Research, University Medical Center Hamburg-Eppendorf, Hamburg-Eppendorf, Germany ^14^Rehabilitation Center Oldenburg, Oldenburg, Germany ^15^Department of Psychosomatic Medicine and Psychotherapy, University of Goettingen Medical Center, Goettingen, Germany ^16^Department of General Practice, University of Goettingen Medical Center, Goettingen, Germany ^17^Geriatric Center Ulm, Ulm, Germany ^18^Department for Thoracic, Cardiac and Vascular Surgery, Ulm University Medical Center, Ulm, Germany ^19^Department of Cardiothoracic, Transplantation and Vascular Surgery, Hannover Medical School, Hannover, Germany ^20^AOK Health Insurance (AOK Lower Saxony) ^21^Department of Cardiac Surgery, Oldenburg Hospital, Oldenburg, Germany ^22^Schüchtermann-Schiller'sche Clinic, Bad Rothenfelde, Germany ^23^Brandenburg Clinic, Bernau Waldsiedlung, Germany ^24^Institute of Cardiology and Sports Medicine, Department Preventive and Rehabilitative Sport and Exercise Medicine, German Sport University, Cologne, Germany ^25^German Heart Foundation, Frankfurt am Main, German
Name and contact information for the trial sponsor {5b}	Innovationsausschuss beim Gemeinsamen Bundesausschuss (G-BA); Gutenbergstraße 13, 10,587 Berlin; Postfach 12 06 06, 10,596 Berlin; Email: info@if.g-ba.de The German Federal Joint Committee is a legal entity under public law Authorized representative: Prof. Josef Hecken; Competent supervisory authority: German Federal Ministry of Health
Role of sponsor {5c}	G-BA is a public sponsor in Germany. The G-BA had no role in the study design; collection, management, analysis, interpretation, or reporting of the data; report preparation; or publication decisions

## Introduction

### Background and rationale {6a}

In 2050, one out of six persons (about 13.6 million people) in Germany will be 75 years or older [1]. With increasing age, the focus of medical care shifts to the maintenance of quality of life (QoL) and functional capacity, which enables older persons to care for themselves as long as possible. A threat to QoL and functional capacity of older individuals are cardiovascular diseases, such as coronary heart disease and degenerative diseases of the heart valves. These cardiovascular diseases will increase in prevalence and incidence as demographic changes occur [2]. Survival rates after cardiac procedures are increasing despite rising age and the associated increase in concomitant diseases. Essential for the high survival rate is the continuous advancement in cardiac medicine and the establishment of gentler interventional procedures [3]. Nevertheless, frail patients with reduced muscle mass, strength, and endurance have a threefold increased risk of postoperative morbidity and mortality [4, 5]. Older cardiac patients also have a significantly increased risk of suffering a cerebrovascular or cardiac event during or after surgery [6]. Especially in geriatric patients, decreased exercise capacity and loss of physical functioning before, during, and after hospitalization have a strong negative impact on QoL, self-determination and everyday resilience [7].

Cardiac interventions are often followed by a long period of convalescence. Postoperative/ postinterventional delirium occurs in 12 to 53% of patients and is associated with a poor long-term outcome in terms of nursing home admission, cognitive decline, reduced independence in daily life, and increased mortality risk [8]. In frail or delirious patients, mortality is up to 40% [9, 10]. It is clear, that older cardiac patients are in need of additional support in order to ensure that their QoL and functional capacity can be maintained—or even improved—after a cardiac procedure. One form of additional support for older patients is a preparatory program, so-called multimodal prehabilitation, which seeks to improve functional capacity, psychological and physical health prior to the cardiac procedure [11].

Currently, there are five systematic reviews with meta-analyses examining the effect of exercise-based prehabilitation prior to cardiac procedures [12–16]. These publications show that exercise-based prehabilitation can shorten the hospital stay [12, 13, 16], improve functional capacity measured by 6-min walking test [12], and reduce various postoperative complications [12, 14–16] such as atrial fibrillation [12] or pulmonary complications [14–16]. However, high heterogeneity of the included studies regarding the therapeutic interventions, the detected outcome parameters, and the cohorts included limit the validity of these findings. A recently published scoping review came to same conclusion [11]. The authors summarized that the evidence is supportive of prehabilitation before cardiac procedures in older and frail individuals but there is a need for an adequately powered, randomized, controlled, assessor-blinded intervention trial to assess the benefit of prehabilitation in improving outcomes in older and frail patients [11]. Especially high-quality multicenter prehabilitation studies prior to an elective cardiac procedure are needed.

Prior to elective cardiac procedures, patients are good candidates for a prehabilitation intervention because of their often advanced age, sometimes long waiting times, and already existing reduced exercise capacity [17]. However, there is no structured cardiac prehabilitation program in Germany that is tailored to the needs of this patient group, and is coordinated across the healthcare sectors.

This intervention trial aims to fill the above described scientific gap. PRECOVERY (Prehabilitation “Karl-Heinz” focusing on cardiac and cognitive functions prior to the cardiac procedure: an analysis of the health status) will evaluate the clinical and health economic effectiveness of prehabilitation in older patients prior to an elective cardiac procedure in a multicenter, two-arm, outcome assessor-blinded randomized controlled trial, comparing the efficacy of a prehabilitation program with standard medical care (SMC). In Germany, SMC for these patients means that they remain at home until the planned cardiac procedure with general instructions (e.g., optimized medication plan) and the opportunity to contact their primary care physicians or cardiologists if they experience physical discomfort. After hospital discharge, all patients have the opportunity to participate in a 3-week in- or outpatient cardiac rehabilitation program that is integrated into the German healthcare system.

Patients will be followed up at intervals for a year after the cardiac procedure, in order to determine the effects of the prehabilitation intervention on 1-year mortality, QoL, and functional capacity, as well as other factors relating to clinical and health economic outcomes.

### Objectives {7}

The objective of this randomized controlled trial (RCT) is to evaluate the efficacy of a 2-week inpatient multimodal prehabilitation intervention called “Karl-Heinz” (*K*ognitiv & k*a*rdiale P*r*ehabilitation vor *He*r*zin*terventionen [cognitive and cardiac prehabilitation prior to cardiac procedures]) for patients aged 75 years or older undergoing elective cardiac procedure (e.g., coronary artery bypass graft surgery or transcatheter aortic valve replacement).

The primary research question is as follows:

Does participation in the “Karl-Heinz” intervention lead to improvements in the co-primary endpoints (QoL/health status and mortality after 12 months) compared with SMC?

The secondary research question is as follows:

Does participation in “Karl-Heinz” lead to improvements in daily function, cardiopulmonary fitness, anxiety and depression, cardiac-specific QoL, and cognition? Additionally, the question if the intervention is associated with reduction of healthcare costs after 12 months compared with SMC will be addressed.

The explanation of further endpoints can be found in the study protocol [see Additional file 1].

### Trial design {8}

PRECOVERY is a confirmatory, randomized, controlled, two-arm parallel group, assessor-blinded multicenter intervention trial. If positive, we will confirm the efficacy of the multimodal prehabilitation intervention with regard to patients’ QoL, medical, cognitive, and psychosocial outcomes 12 months after an elective cardiac procedure. We will randomize 422 patients aged 75 years or older scheduled for an elective cardiac procedure to either (a) SMC (*n* = 211) or (b) a 2-week multimodal prehabilitation intervention (“Karl-Heinz”, *n* = 211) with an allocation ratio of 1:1 per hospital, patient’s age and gender with a follow-up period of 12 months. An additional component of the RCT is a detailed health economic analysis by the Department of Health Economics and Health Services Research, University Medical Center Hamburg-Eppendorf, Germany. Furthermore, an independent process evaluation in a mixed-methods design will be provided by the Department of General Practice of the University Medical Center Goettingen, Germany.

The primary aim is to demonstrate superiority of the intervention group compared to SMC group in terms of QoL and mortality 12 months after the cardiac procedure. The secondary aim is to show the superiority of the intervention group compared to the SMC group in terms of functional and cognitive capacity, heart-specific QoL, and activities of daily living after 12 months.

For the health economic analysis, we anticipate at least equivalence, a better non-inferiority in healthcare costs after 12 months in the intervention group compared to SMC group.

## Methods: participants, interventions, and outcomes

### Study setting {9}

In this study, a new form of care for older heart patients is being implemented in partnership with a large German health insurance company, AOK Lower Saxony. Nine German departments for cardiac surgery and/or cardiology will recruit patients (primarily those who are insured by the AOK Lower Saxony) scheduled for an elective cardiac procedure. The 2-week multimodal prehabilitation intervention “Karl-Heinz” will be provided in seven inpatient cardiac-specific rehabilitation hospitals, and one outpatient rehabilitation hospital (prehabilitation centers).

**Fig. 1:**
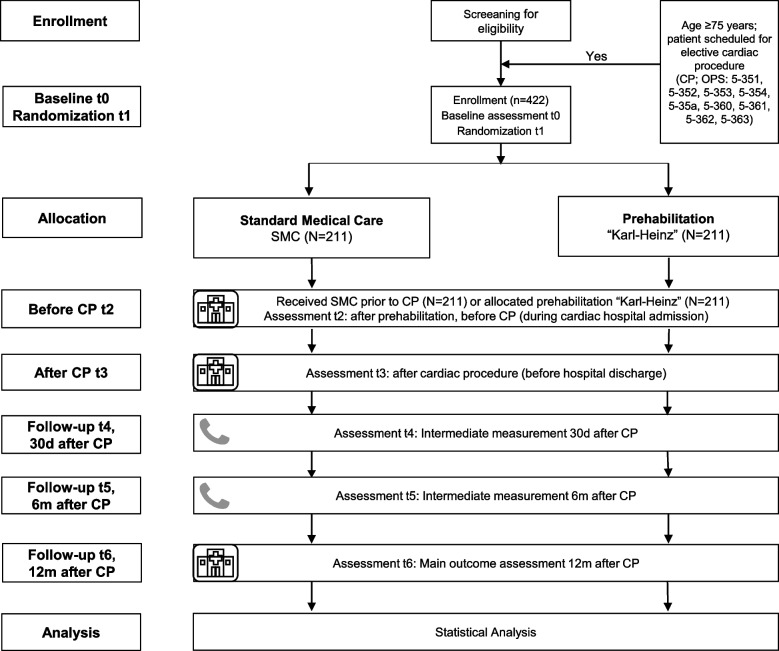
PRECOVERY flowchart. Abbreviations. CP = cardiac procedure; OPS = operation and procedure codes; SMC = standard medical care

A current list of all participating clinics and prehabilitation centers, as well as all scientific partners, can be found online at the project homepage (https://herzzentrum.umg.eu/precovery/). Likewise, in the supplemental material is a German and English version of the study protocol. The flow diagram of the study is presented in Fig. 1.

### Eligibility criteria {10}

We will include patients 75 years or older undergoing elective cardiac procedures defined by the German operation and procedure codes catalog (OPS, see Table 1). Due to legal reasons to the project funding participation of patients included in Lower Saxony is limited to patients of AOK Lower Saxony public health insurance provider.

**Table 1:** Explanation of the translated OPS Code

OPS Code and Procedure
5–35: Operations on heart valves and septa as well as on vessels close to the heart
5–351: Replacement of heart valves with prostheses
5–352: Change of heart valve prostheses
5–353: Valvuloplasty
5–354: Other operations on heart valves
5-35a: Minimally invasive surgery on heart valves
5–36: Coronary artery surgery
5–360: Desobliteration (endarterectomy) of coronary arteries
5–361: Creating an aortocoronary bypass
5–362: Creating an aortocoronary bypass using minimally invasive technique
5–363: Other revascularization of the heart

*Abbreviations OPS* operation and procedure codes

An adapted recruitment strategy has been developed as an option for the case that the number of eligible ≥ 75 years old AOK-insured persons is not adequate to reach our recruitment goals. First, the age requirement will be lowered by 10 years to include all retired persons ≥ 65 years. PRECOVERY is powered to EQ-5D-5L with a minimal clinically relevant effect. Current literature shows a positive effect of prehabilitation on QoL also in patients who are ≥ 53 years of age [18, 19]. The costs for prehabilitation are covered by the health insurance for retired persons; however, the pension fund is responsible for such costs for employed persons. Therefore, PRECOVERY is only open to retired persons. Second, by continued recruitment problems the study will be opened to include members of all health insurance companies.

Inclusion and exclusion criteria are summarized in Table 2.

**Table 2:** PRECOVERY inclusion and exclusion criteria

**Inclusion criteria**
Age ≥ 75 years
Planned elective cardiac procedure (OPS: 5–351 to 354, 5-35a, 5–360 to 363)
Sufficient independence, ability to care for self and participate in prehabilitation
Insurance status of all patients from Lower Saxony to patients of AOK (public health insurance provider)
Ability to give consent
Willingness to participate voluntarily in the study after informed consent with signed consent form
Ability to speak, read, and understand German
**Exclusion criteria**
Lack of capacity to consent
Katz index 0
Need for treatment in an acute care hospital
Severe dementia or severe mental disorders, acute delirium
Diagnosis of acute alcohol or drug abuse
Unstable angina pectoris
Heart failure, NYHA IV
Myocarditis, hypertrophic obstructive cardiomyopathy, main stem stenosis ≥ 80%
Severe refractory cardiac arrhythmias
Recent aortic dissection
Peripheral arterial occlusive disease stage ≥ III according to Fontaine
Symptomatic carotid stenosis or carotid stenosis requiring therapy
Renal insufficiency requiring dialysis
Hepatic insufficiency, child B and child C
Advanced (metastasized) oncological disease
Neurological, orthopedic or rheumatic comorbidities that militate against physical training
Participation in another intervention study (participation in registry studies is allowed)

*Abbreviations* *OPS* operation and procedure codes, *NYHA* New York Heart Association

### Who will take informed consent? {26a}

Prior to baseline assessment, eligible patients and their relatives will be informed about the study goals, duration of the study, the role of each participant, randomization, and any risks in written and oral forms. After written informed consent from patients, and their relatives by a study physician, baseline assessment (t0) will take place and patients will be randomized (t1). Original patient information and consent forms are available on request from the corresponding author. If a patient does not have any relatives, then persons close to him or her can be consulted. Participation in the study is also possible without relatives or close persons.

### Additional consent provisions for collection and use of participant data and biological specimens {26b}

Enrolled participants are offered the opportunity to participate in a voluntary sub-study where biological samples will be analyzed. This specific research topic in the study population is not covered by the grant of this trial.

## Interventions

### Explanation for the choice of comparators {6b}

The “standard medical care” comparator appears to be the appropriate option for a pragmatic effectiveness analysis. The control group will receive an information folder with general information and SMC provided as part of the cardiac procedure process.

### Intervention description {11a}

In contrast, the intervention group will receive a 2-week individualized prehabilitation program “Karl-Heinz” prior to the elective cardiac procedure. Study participation will have no influence on perioperative processes.

### Prehabilitation

Patients in the intervention group receive a 2-week preventive, intensive, and full-day prehabilitation in selected cardiac-specific rehabilitation facilities (prehabilitation centers) for targeted, holistic preparation for the cardiac procedure. Prehabilitation comprises a multimodal, interdisciplinary therapeutic approach individually adapted to the patients’ needs in order to achieve the best possible physical and mental health status with improved functional reserve prior to the planned cardiac procedure. The prehabilitation program “Karl-Heinz” includes seven modules: (1) sports and exercise therapy, (2) occupational therapy, (3) cognitive training, (4) psychosocial support, (5) disease-specific education, (6) informative talks with relatives, and (7) special hygiene training/nutritional intervention [details see Additional file 3].

To standardize the prehabilitation program, a treatment manual [details see Additional file 3] and a minimum of therapy requirements (see Table 3) was designed in a multidisciplinary team from all participating centers and the two participating health insurance companies (AOK Lower Saxony and AOK Baden-Württemberg). Based on this, a train-the-trainer concept with detailed standard operating procedures (SOPs) and additional training videos was created. All coordinating team members of each prehabilitation center (the so-called “multipliers”) will receive 2 days of train-the-trainer instruction on the individual modules of the treatment manual “Karl-Heinz”. These persons are trained to perform their tasks as “multipliers” a few weeks before the first patient is enrolled.

**Table 3:** Overview of the minimum of therapy requirements during prehabilitation program “Karl-Heinz”

**Form of therapy**	**Minimum of requirements**
(1) Sports and exercise therapy	Scope: 1 active unit per day (5 × per week) of 30 min each
	Content: endurance, strength/flexibility, coordination training
(2) Breathing therapy	Scope: 3 times per week of 30 min each
	Content: Group setting with daily self-training
(3) Occupational therapy	Scope: 2 times per week of 30 min each
	Content: see exercise therapy plus tips for the sustainability of prehabilitation
(4) Cognitive training	Scope: 2 times per week of 30 min each
	Content: attention and concentration training
(5) Psychosocial intervention	Scope: 2 times during prehabilitation period
	Content: Delirium education, preparation for surgery inclusive procedures in the hospital and PMR
(6) Disease-specific education	Scope: 3 units in the prehabilitation period, video training if necessary
	Content: sleep, nutrition, exercise, risk factors, mindfulness, social counseling
(7) Informative talks with relatives	Scope: 1 time during the prehabilitation period
	Content: see treatment manual in the additional file 3

Subsequently, on-site training sessions will be conducted by the multipliers for the intervention staff of the respective prehabilitation centers. The individual elements of the “Karl-Heinz” prehabilitation are not new, but the embedding of a heart-specific program in a structured trans-sectoral concept represents a previously unaddressed need in the care of older patients.

The initial assessment of the individual exercise capacity is carried out by the responsible physicians of the prehabilitation center. Experienced therapists are in regular exchange with the medical staff to individually increase the exercise intensity depending on the primary cardiac disease.

The contents of the individual training interventions are based on current study results, surveys of the participating prehabilitation centers, and interviews with experts. Due to the limited number of studies and the high heterogeneity of existing prehabilitation training intervention studies in cardiological patients, the design of the training forms was based on existing secondary prevention recommendations for cardiological sports and exercise therapy [20–22].

### Criteria for discontinuing or modifying allocated interventions {11b}

During the prehabilitation intervention, the supervising study physicians of the prehabilitation center will obtain feedback on the participants’ treatment experience minimum twice weekly during medical rounds. Therefore, the multimodal prehabilitation intervention can be adjusted at any time if necessary.

Adverse events (AEs) including serious adverse events (SAEs) will be documented in the medical record and transmitted to the recruiting center prior to the cardiac procedure. The cardiovascular AEs in form of major cardiac events (MACE) and predefined SAEs will be documented into the secuTrial® electronic database from the medical record after the prehabilitation intervention. To ensure blinding of study assessors, a non-blinded study investigator will fill cardiovascular AEs and SAEs of patients in the SMC and intervention group into the secuTrial® database at t2. An independent Data Safety Monitoring Board (DSMB) including national and international experts on cardiology, cardiac surgery, geriatrics, cardiac rehabilitation, prehabilitation, and patient advocates has been established. The DSMB reviews the cardiovascular AEs and SAEs of the prehabilitation intervention and SMC before the cardiac procedures on a quarterly basis and determines whether the safety of the patients is still ensured and whether the continuation of the study without any changes is justifiable, or—if necessary—makes recommendations for discontinuation or modifications of the study. To fulfill this task, the DSMB receives information about protocol deviations, the status of patient recruitment and the observed cardiovascular AEs and predefined SAEs.

### Strategies to improve adherence to interventions {11c}

All patients will fill out a daily diary and evaluate each therapy session. Adherence will be assessed by reviewing these documents. The team of the process evaluation will be responsible for the evaluation of the diaries. Likewise, close contact with the multiplier, regular visits, and therapies as individual therapy and in small groups should contribute to patients’ motivation and adherence to therapy.

### Relevant concomitant care permitted or prohibited during the trial {11d}

Participation in other intervention studies is prohibited (see exclusion criteria). However, it is allowed to treat the laboratory parameters collected in the recruiting clinic in case of abnormalities during prehabilitation. Likewise, medications can be adjusted for discomfort and/or cardiac and/or non-cardiac abnormalities.

### Provisions for post-trial care {30}

The study ends for all participants after the assessment t6 (follow-up 1 year after the cardiac procedure). All patients will sign the patient consent at the beginning of the study which includes the information that they agree to voluntarily participate in the clinical trial. The patient consent/information is available on request by the corresponding author. Participants carry no additional costs for post-trial care due to the fact that the German healthcare system does not require those who are insured to pay deductibles, co-payments, or out-of-pocket fees to receive medical care.

### Outcomes {12}

The co-primary outcomes are the QoL and mortality after 12 months. The QoL is measured by the EuroQol questionnaire (EQ-5D-5L, [23]). The EQ-5D-5L consists of five dimensions (mobility, self-care, usual activities, pain/discomfort, and anxiety/depression) and is an instrument for measuring QoL and determining quality-adjusted life years in health economic studies [23, 24]. Each dimension is scored on a 5-point scale. The responses to the individual items are combined into a QoL index using established algorithms. In addition, patients rate their health status on a visual analog scale (EQ-VAS; range 0–100) [23].

The following secondary outcomes measures are defined:

1. Activities of daily living (ADL) measured by Katz Index [25, 26]
2. Physical performance rated by Short Physical Performance Battery (SPPB; [27])
3. Cognitive performance measured by Montreal Cognitive Assessment Test (MoCa; [28, 29])
4. Disease-specific QoL assessed by Heart Quality of Life Questionnaire (HeartQoL; [30])
5. Anxiety and depression detected by Hospital Anxiety and Depression Scale (HADS; [31–33])
6. Healthcare costs after 12 months. Healthcare resource utilization within 12 months after the cardiac procedure. During follow-up, all direct medical and non-medical healthcare-related resource utilization will be monitored using a validated questionnaire for health-related resource use by older patients (Fragebogen zur Inanspruchnahme medizinischer und nicht-medizinischer Versorgungsleistungen im Alter (FIMA; [34]) in combination with routine data transmitted by the AOK Lower Saxony and AOK Baden-Württemberg.

Further endpoints are as follows:

Thirty-day mortality, length of stay in intensive and normal care unit, AE/SAE assessment, hand strength measured by Marin-Vigorimeter [35, 36], functional capacity collected by the 6-min walk test [37], bioelectrical impedance analysis to elevate body composition [38], Mini Nutritional Assessment to detect malnutrition [39, 40], Pittsburgh Sleep Quality Index to detect sleep quality [41–43], Maastricht Questionnaire to evaluate the vital exhaustion [44, 45], Informant Questionnaire on Cognitive Decline in the Elderly to assess opinion of relatives (IQCODE; [46]; Zarit Burden Interview to measure burden of relatives (G-ZBI; [47]), Clinical Frailty Scale to assess frailty (CFS; [48]), American Thoracic Society (ATS) scale to evaluate dyspnea (dyspnea scale of the ATS; [49]), self-reported subjective memory impairment (SMI; [50]) to measure patients’ opinion about the own memory performance, Selbstwirksamkeits-überzeugung ([general self-efficacy scale], SWE) scale to identify self-efficacy expectancy [51–53], Hamburger Fragebogen zum Krankenhausaufhalt [Hamburg Questionnaire on Hospital Stay] to assess patients’ satisfaction with the hospital stay [54], optimism measured by Life-Orientation-Test revised (LOT-R; [55]), loneliness detected by UCLA Loneliness Scale [56, 57], illness perception measured by Illness Perception Questionnaire (IPQ; [58]), treatment expectancy measured by Treatment Expectation Questionnaire (TEX-Q; [59]), and institutionalization/dependence on care assessed in Clinical data / sociodemographic interview.

The mixed-methods process evaluation will evaluate intermediate outcomes (dose, reach, fidelity, adaptions) of the intervention [60]. It assesses the implementation of the prehabilitation itself and the reactions and perspectives of involved persons. Moreover, barriers and facilitating factors will be explored [61]. More details will be published in a separate study protocol.

### Participant timeline {13}

Table 4 summarizes the schedule of enrolment, intervention, and assessments in the PRECOVERY trial.

**Table 4:** Schedule of enrolment, intervention, and assessments in the PRECOVERY trial

Time point	t0 (Screening)	t1 (Randomization/ Baseline)	t2 (Before CP)	t3 (After CP)	t4 (30 days after CP)	t5 (6 months after CP)	T6 (12 months after CP)
Inclusion/Exclusion criteria	x						
Informed consent, enrollment, allocation	x						
Medical history, social demographics		x					
**Primary Outcomes**							
EQ-5D-5L (QoL)		x	x	x	x	x	x
1-year mortality							x
**Secondary Outcomes**							
Katz Index (daily activity)		x	x	x	x	x	x
SPPB (physical performance)		x	x				x
MoCa (mental performance)		x	x				x
HeartQoL (disease-specific QoL)		x	x		x	x	x
HADS (anxiety and depression)		x	x		x	x	x
**Other Outcomes**							
30-day mortality					x		
Hand grip strength (frailty)		x	x				x
6-min walk test (functional capacity)		x	x				x
BIA (body composition)		x	x				x
MNA (frailty, malnutrition)		x	x				x
PSQI (sleep quality)		x	x				x
Maastricht questionnaire (vital exhaustion)		x	x		x		x
UCLA Loneliness Scale (loneliness)		x			x		x
SMI (memory impairment)		x	x		x	x	x
CFS (frailty)		x	x	x			x
Pre- and post-procedure complications				x			
Length of stay in intensive and normal care				x			
Institutionalization/dependence on care		x	x	x	x	x	x
ATS scale (dyspnea)		x	x	x	x	x	x
SWE scale (general self-efficacy)		x			x	x	x
HFK (patients ‘ satisfaction with hospital stay)				x			
LOT-R (optimism)		x	x				
IPQ-B (illness perception)		x	x				x
TEX-Q (treatment expectancy)		x	x				
Process evaluation		x	x	x	x	x	x
FIMA Questionnaire (healthcare-related resource utilization)		x				x	x
Blood sample		x	x	x			x
AE/SAE			x	x	x	x	x
**Relatives:** G-ZBI (burden of relatives)		x				x	x
**Relatives:** IQCODE (opinion of relatives)		x				x	x

Abbreviations: *AE* adverse event, *ATS* American Thoracic Society, *BIA* Bioelectrical Impedance Analysis, *CP* cardiac procedure, *CFS* Clinical Frailty Scale, *d* days, *EQ-5D-5L (QoL)* Euro Quality of Life, *FIMA* Fragebogens zur Erhebung von Gesundheitsleistungen im Alter [Questionnaire for the survey of health services in old age], *G-ZBI* German Zarit Burden Interview, *HADS* Hospital Anxiety and Depression Scale, *HeartQoL* Heart Quality of Life, *HFK* Hamburger Fragebogen zum Krankenhausaufhalt [Hamburg Questionnaire on Hospital Stay], *IPQ-B* Illness Perception Questionnaire, *IQCODE* Informant Questionnaire on Cognitive Decline in the Elderly, *LOT-R* Life Orientation Test Revised, *m* month, *MNA* Mini Nutritional Assessment, *MoCa* Montreal Cognitive Assessment Test, *PSQI* Pittsburgh Sleep Quality Index, *SAE* serious adverse event, *SMI* Self-reported subjective memory impairment, *SPPB* Short Physical Performance Battery, *SWE* Selbstwirksamkeits-überzeugung ([Self-efficacy beliefs], *TEX-Q* Treatment Expectation Questionnaire, *UCLA* University of California Los Angeles

### Sample size {14}

A total number of 422 patients is planned for this intervention trial. The number of cases was planned on a confirmatory basis in order to be able to answer the primary questions with a power of 80%. A total of 338 patients (169 per treatment group) is sufficient to show a clinically relevant difference of 0.045 points in EQ-5D-5L at t6 with 80% power at the generally accepted two-sided significance level of 5%. It is assumed that the common standard deviation of the EQ-5D-5L is 0.17 and that the observations on the covariates have an R-squared of 0.25. Since this is a longitudinal study, a dropout rate of up to 20% is assumed, so a total of 422 patients will be recruited. The case number calculation was performed in nQuery version 8. The definition of a clinically relevant difference of 0.045 was based on the publication of McClure et al. [62], who restricted a minimal clinically relevant difference to values between 0.037 and 0.069 [62]. Since the publication did not include results on the German population, a value was chosen that was approximately 10% lower than the average of the countries considered in the publication. The assumption of a standard deviation of 0.17 is based on observations by [63], who observed a standard deviation of 0.171 at baseline and a standard deviation of 0.170 at 12-month follow-up in a sample of 1927 diabetes-2 patients [63].

### Recruitment {15}

The recruitment measures in PRECOVERY are integrated into the standard procedures of the participating recruiting centers. In these centers, patients scheduled for cardiac procedures in 4–6 weeks will be screened, informed, recruited (t0), and randomized (t1) according to the inclusion and exclusion criteria during a preoperative appointment in cardiology or cardiac surgery departments. To support the recruitment process, all referring centers will be informed about the study in advance and additional flyers will be produced to increase patient interest.

If the recruitment is not running well, the evaluation concept is designed through the use of the FIMA questionnaire in such a way that an extension to the inclusion of insured persons from other health insurance funds is also conceivable in Lower Saxony on the basis of the legal basis §630a German civil code.

## Assignment of interventions: allocation

### Sequence generation {16a}

After baseline assessment, randomization will be 1:1 stratified by study center, patient age (< 81 years vs. ≥ 81 years), and gender (block randomization with random block lengths). Randomization will be performed by unblinded study physicians at the recruiting centers. After randomization, the patient will be informed about the respective allocation. In case of allocation into the intervention group, the prehabilitation center will be contacted by the unblinded study physician. The individual outcome assessors at the recruiting centers remain blinded.

### Concealment mechanism {16b}

Participants will be randomized using secuTrial®, the electronic database for the pseudonymized data management of the PRECOVERY trial. Allocation concealment will be ensured, because the unblinded trial physician will not press the button for allocation until the patient has been recruited into the trial. The baseline assessments will be completed by an outcome assessor who is blinded to the allocation of the patient in intervention or SMC group.

### Implementation {16c}

The allocation order is determined by the Institute of Medical Statistics of the University Medical Center Goettingen, Germany.

## Assignment of interventions: blinding

### Who will be blinded {17a}

Due to the nature of the intervention, all participants and staff members in the prehabilitation centers cannot be blinded. Therefore, blinding to group allocation will be limited to outcome assessors.

### Procedure for unblinding if needed {17b}

In the recruiting centers, the outcome assessors are determined to be the blinded study personnel. Once unblinded, outcome assessors cannot perform tasks that require blinding. Unblinding must be reported in the deviation log, in the secuTrial® database as well as for the recruiting center in a correspondingly suitable place, e.g., assignment list.

## Data collection and management

### Plans for assessment and collection of outcomes {18a}

The various assessments to be applied in the different data collection dates are shown in Table 4 and in the translated approved study protocol [Additional file 1].

The medical history includes age, height, weight, hip to waist ratio, blood pressure, heart rate, cardiovascular risk factors, and other cardiac and non-cardiac conditions. Previous cardiac and vascular surgical procedures will be also documented. Sociodemographic data will be included in the database in the form of marital status and education level, among others. Routine laboratory chemistry data before and after the cardiac procedures will be incorporated into a standardized laboratory protocol. Furthermore, we will complete a cardiac surgery or cardiac intervention protocol detailing the cardiac procedure. The total length of inpatient stay, the length of stay in the intensive care unit, and the performance as well as duration of cardiac rehabilitation will be recorded. Likewise, before and after the cardiac procedure, the occurrence of dyspnea will be documented. Postoperative/postinterventional complications (e.g., hemorrhage, pleural effusions, pneumothorax, cardiac arrhythmias) as well as mortality after 30 days and 1-year are systematically recorded. In order not to overlook the occurrence of delirium, the patient record is reviewed. If there are signs of severe agitation or other delirious symptoms according to ICD-10, the patient is classified as delirious. All peri- and postoperative data will be collected from the hospital electronic database or routine clinical documentation.

General QoL will be assessed by EQ-5D-5L [24] and disease-specific QoL by HeartQol [30, 64].

Functional assessments include the SPPB [27, 65], 6-min walk test [37], and handgrip strength [35, 36]. Daily activity will be measured by Katz Index [25, 26].

Frailty will be identified with the CFS [48], the Mini Nutritional Assessment [39, 40], and in a subgroup analysis by Bioelectrical Impedance Analysis [38].

Cognitive function will be screened with MoCa [28, 29, 66], SMI [50], and IQCODE [46].

Psychosocial assessments include the HADS [31–33], Pittsburgh Sleep Quality Index [41–43], Maastricht Questionnaire [44, 45], SWE scale [51, 52], LOT-R [55], UCLA Loneliness Scale [56, 57], IPQ [58], and TEX-Q [59].

A modified version of the Hamburg Questionnaire on Hospital Stay will be assessed after the cardiac procedure, and G-ZBI will be used to measure the burden relatives experience during the perioperative time [47].

Healthcare costs will be collected using the FIMA questionnaire [34] in combination with routine data from the AOK (health insurance data).

Adherence to the prehabilitation program is monitored through documentation of each session created by the process evaluation team. A patient diary is completed by the patient after each session in order to evaluate patient performance afterwards.

All data collected in paper form are documented in secuTrial® (eCRF) by the outcome assessors immediately after the survey. Data entry is based on the 4-eye principle—each entry is checked again by another person.

Finally, optional blood samples will be collected and analyzed on four data collection dates to examine neurodegenerative, inflammatory, cardiac, genomic, and epigenetic markers, approximately 50 ml each.

For the process evaluation, quantitative data will be collected in standardized interviews with patients, their relatives, and professionals involved in the prehabilitation. Further data will be collected via patient diaries and therapy plans. In addition, qualitative data (interviews and focus groups) will be gathered to take into account the perspectives of patients and professionals involved in the prehabilitation process. The goal is to better understand the implementation of the intervention and its consequences.

All principle investigators, outcome assessors (1 day), and prehabilitation center multipliers (2 days) will receive an intensive training prior to enrollment of the first patient.

### Plans to promote participant retention and complete follow-up {18b}

In order to strengthen participant retention, e.g., travel costs for the journey to prehabilitation and back home as well as to t6, the last survey, are covered. At the individual survey times, patients also receive examinations outside of routine care, which can help to detect abnormalities at an early stage. To emphasize that, the outcome assessors point this out at the survey times to promote compliance.

### Data management {19}

Details on data management are described in detail in a data management plan. Assessments are recorded on paper and data are entered remotely via an electronic case report form (eCRF) and stored pseudonymously in a provided secuTrial® database, an established GCP-compliant web solution. The secuTrial® data entry screens resemble the paper forms used. Blinded outcome assessors enter data in the secuTrial® database. This data is confirmed by a second person in an independent step.

### Confidentiality {27}

The data for the evaluation stored in the secuTrial® are made available to the evaluating institutions via encrypted email program (e.g., Cryptshare). In addition to the data from the eCRF, pseudonymized routine data from the AOK are also made available for the health economic evaluation. These data are transmitted to the evaluating institution via a secure connection. The identification of the study participants at the health insurance companies is done via the insurance number, which is collected when the patients are enrolled in the study and transmitted to the respective AOK together with the study ID. The original paper-based completed questionnaires will be stored securely and destroyed after 10 years in accordance with the legal conditions of data protection.

### Plans for collection, laboratory evaluation, and storage of biological specimens for genetic or molecular analysis in this trial/future use {33}

In case if participants voluntarily consented to the supplemental study up to 50 ml of blood will be collected per visit (t0/t1, t2, t3, t6) and stored as cellular as well as non-cellular components. The focus is to detect neurodegenerative, inflammatory, and cardiac markers, genomic and epigenetic markers (e.g., ApoE-Genotyps) out of these biospecimens. The processing and storage of biospecimens (serum, plasma, RNA from PAXgene tubes) will be performed according to locally established SOPs. This is a multicenter study, thus blood samples will be collected using identical SOPs for processing and intermediate storage at different study sites. Finally, samples will be shared, exchanged, and analyzed under a common agreement. This means that the blood samples and medical data collected will first be stored, analyzed, and used at all study sites within the project. Because the blood-based biomarkers are valuable data and samples that will be of great value for research, there are no plans to destroy the samples. If a patient withdraws from study participation, he/she may decide whether biospecimens that have already been stored may either be anonymized, may be further used or must be destroyed and data deleted.

## Statistical methods

### Statistical methods for primary and secondary outcomes {20a}

Details on statistical analysis are described in a statistical analysis plan (see Additional file 2). The primary endpoint EQ-5D-5L will be evaluated using a joint model for longitudinal data. Stratification factors of age, sex, participation in DMPs, and center will be included as covariates in the model. Time courses of EQ-5D-5L are presented longitudinally for each group, and survival rates are presented descriptively using Kaplan–Meier curves. Treatment effects are reported with 95% confidence intervals. The secondary endpoint HeartQoL is evaluated analogously to the primary endpoint. Secondary endpoints Katz Index, SPPB, MoCa, and HADS are evaluated using mixed linear models, with the factors time point, treatment, and the interaction, and the covariates age, sex, DMP participation, and center. Marginal means are calculated for inter- and intra-group comparisons, and treatment effects are reported with 95% confidence intervals. Likewise, for the analysis of the health economic outcomes, mean differences are calculated in mixed regression models. Because of the skewed distribution of the cost data, the non-parametric bootstrapping procedure, which is also used in the uncertainty analysis of the incremental cost-effectiveness ratio (ICER), is additionally used to calculate the standard errors of the regression parameters. For the analysis of cost-effectiveness, the net-benefit regression procedure is used. Details can be found in the Additional file 1 “Study Protocol”.

Statistical methods in the process evaluation:

For the process evaluation, exploratory sensitivity analyses will be performed to assess the impact of dose, reach, fidelity, and adaption on the intervention’s efficacy. For this purpose, interaction-terms between the treatment variables and these factors will be included in the mixed linear models described above. In addition to the intention-to-treat population, a population will be defined that started the intervention as intended and stays adherent during the follow-up period (starting and adhering estimand).

### Interim analyses {21b}

An interim analysis will take place after 25% of the recruited patients. Furthermore, the DSMB reviews the study progress on a quarterly basis. In order to fulfill this task, the DSMB receives information about protocol deviations, the status of patient recruitment, and the observed cardiovascular AEs and SAEs.

### Methods for additional analyses (e.g., subgroup analyses) {20b}

Interviews and focus groups of the qualitative process evaluation will be analyzed by content analysis [67]. Later on, findings will be cross-mapped with results of the quantitative process evaluation according to the research interests. Methods will be described in more detail in the study protocol for the process evaluation (in preparation).

No prespecified subgroup analyses are planned.

### Methods in analysis to handle protocol non-adherence and any statistical methods to handle missing data {20c}

Participants will be considered adherent to the intervention if they complete the minimal requirements (see Table 3). For participants to be included, they must have baseline values and at least one follow-up value, then the missing data will be imputed using multiple imputation. Details on the type of multiple imputation will be added following a blinded data review.

### Plans to give access to the full protocol, participant-level data, and statistical code {31c}

The full protocol is uploaded and publicly available on the project homepage (https://herzzentrum.umg.eu/precovery/). All plans for granting access to the participant-level dataset, participant dataset, and statistical code are regulated in the project’s internal publication rules. All the principle investigators have been informed accordingly.

## Oversight and monitoring

### Composition of the PRECOVERY coordinating center, lead investigators, general assembly, trial steering committee, and monitoring {5d}

#### PRECOVERY coordinating center

The PRECOVERY coordinating center is made up of the consortium leader (Prof. Christine von Arnim) and her deputy (Dr. Monika Sadlonova) as well as two project coordinators (Dr. Carolin Steinmetz and Dr. Stephanie Heinemann). One project coordinator is responsible for the “Karl-Heinz” intervention and the processes involved with the RCT (Dr. Carolin Steinmetz). The other project coordinator is responsible for research management and contractual aspects (Dr. Stephanie Heinemann).

#### Lead investigators

In each participating recruiting center, a lead investigator (senior physician with expertise in cardiology or heart surgery) is responsible for the adherence to study protocol, including identification, recruitment, data collection, and completion of CRFs at several time points. The lead investigator is responsible for training personnel in the recruiting center and communicating with the PRECOVERY coordinating center and the personnel of the prehabilitation center.

In each participating prehabiliation center, a lead investigator (senior physician with expertise in cardiac rehabilitation) is responsible for the adherence to study protocol, including training all involved prehabilitation personnel to provide the intervention “Karl-Heinz” according to the intervention manual. The lead investigator is responsible for the communication with the PRECOVERY coordinating center and the unblinded physicians in the recruiting center.

#### General assembly

The General Assembly is made up of the lead investigators from the recruiting and prehabilitation centers. In addition, the consortium leader, head statistician, and the representatives of additional project partners, such as AOK Health Insurance (AOK Lower Saxony), the Department of Health Economics and Health Services Research, University Medical Center Hamburg-Eppendorf (economic evaluation), Department of General Practice of the University Medical Center Goettingen (process evaluation), and the Department of Psychosomatic Medicine and Psychotherapy (intervention training concept). The General Assembly meets once quarterly to discuss the progress of the study, current challenges, and any changes to the protocol deemed necessary by the Steering Committee.

### Steering committee

One investigator from a recruiting center and one investigator from a prehabilitation center are voted to the Steering Committee, joining the consortium leader, a representative of the AOK Health Insurance (AOK Lower Saxony) and the head statistician. The Steering Committee is responsible for regularly (at least once per quarter) reviewing the progress of the study and, if required, making changes to the protocol to ensure the smooth running of the study.

#### Monitoring

The Clinical Trials Unit of the University Medical Center Goettingen provides the personnel and know-how for the monitoring of the PRECOVERY randomized controlled trial and ensures data integrity.

### Composition of the data safety management board, its role and reporting structure {21a}

The DSMB is made up of four renowned experts in the field and a member of the German Heart Foundation (Deutsche Herzstiftung) as patient representative. The DSMB meets quarterly via online video meeting with the consortium leader during the patient recruitment phase of the PRECOVERY project to review AEs and SAEs reported in the follow-up time points after the “Karl-Heinz” intervention. The DSMB will assess the trial progress and safety, and accumulated trial data will be reported to the DSMB by the study statistician. The four experts in the DSMB receive a small expense allowance for their participation in the activities of the PRECOVERY DSMB—the German Heart Foundation does not receive any expense allowance. At their discretion, the DSMC may also formulate recommendations relating to the selection/recruitment of participants, their management, improving adherence to protocol-specified regimens and matter relating to patient retention, and procedures for data management and quality control.

### Adverse event reporting and harms {22}

A selection of AEs and SAEs will be recorded to monitor certain safety aspects of the study intervention. After randomization, all adverse cardiovascular events, revascularization procedures, or other invasive cardiac interventions and all SAEs (cardiac and non-cardiac reasons) will be documented in the eCRF. As mentioned above, the DSMB will assess the trial progress and safety, making appropriate recommendations for discontinuation or modifications of the study as needed.

### Frequency and plans for auditing trial conduct {23}

An independent member of the Clinical Trials Unit of the University Medical Center Goettingen will act as a monitor and regularly review the recruitment centers. After a site initiation visit, the monitor will visit once a year the recruitment facilities and conduct an interim visit. The monitor will review documentation of AEs and SAEs, patient and study records. Prehabilitation centers will be monitored indirectly by reviewing medical records sent to recruitment centers after prehabilitation by monitor. During the first year of the intervention, prehabilitation centers will be visited by a consortium team member for follow-up as well as refresher training in the “Karl-Heinz” treatment manual.

### Plans for communicating important protocol amendments to relevant parties (e.g., trial participants, ethical committees) {25}

Any changes to the protocol that affect or could affect the study design or procedures, the objectives and hypotheses, or patient safety must be submitted as an amendment to the ethics committees of the study centers for consultation. The new version of the study protocol will be made available at the projects website, including the rationale for any changes. The clinical trial entry will be adjusted if necessary. Minor changes, such as organizational adjustments, changes in written manuals for the pure reason of clarification of any processes, or changes in responsibilities that have no effects on the defined study goals and conduction, will be agreed upon by the consortium management of PRECOVERY. In such cases, the ethical committee of the leading study center will be notified of such changes.

### Dissemination plans {31a}

The study report of the main outcomes will be submitted for publication to a peer-reviewed medical journal. Furthermore, a study report must be submitted to the Federal Joint Committee (G-BA). A summary report of the final study results will be disseminated to all project partners and on the project homepage. Authorship in scientific publications will adhere to the recommendations of the International Committee of Medical Journal Editors [68].

## Discussion

PRECOVERY is a randomized controlled multicenter trial with a calculated sample size of 422 participants that aims to examine the long-term efficacy (after 12 months) of a tailored prehabilitation for patients aged 75 years or older undergoing elective cardiac procedures. RCTs focusing on cardiac prehabilitation have shown positive effects in terms of delirium rate, QoL, length of stay in intensive care unit, and improved compliance of postoperative rehabilitation with good safety [69, 70]. Likewise, pre- and postoperative psychological interventions before cardiac surgery additionally led to significant reduction in length of hospital stay and increase in self-efficacy [71, 72]. In the last decade, five reviews with meta-analyses emphasizing on exercise-based prehabilitation prior to elective coronary procedure have been published [12–16] and have shown positive effects as well. In fact, multimodal intervention studies are rare. Mostly, only single-intervention modules were addressed in the context of prehabilitation. Two or three different modules have hardly been conducted. However, it is known from prehabilitation intervention studies with cancer patients that a multimodal prehabilitation approach should include at least physical activity training, nutritional counseling, and psychological support to be effective [73, 74]. In the field of cardiology/cardiac surgery, evidence-based conclusions are lacking—but parallels seem obvious.

The main cohort included in cardiac prehabilitation studies are patients prior to coronary artery bypass graft surgery and/or valvular surgery. To the best of our knowledge, there is currently no evidence proving the effect of a multimodal prehabilitation prior to cardiac intervention such as catheter-based valve reconstructions (e.g., MitraClip, TriClip).

PRÄP-GO [75] and PRECOVERY are the first multicentric prehabilitation trials in Germany focusing on the target to include prehabilitation before elective procedures in older patients into the German healthcare system.

Both studies are funded by the “Innovationsauschuss” [Innovation Committee] of the Federal Joint Committee (G-BA). The objective of PRÄP-GO is to evaluate the efficacy of a 3-week prehabilitation program for patients ≥ 70 years of age with frailty or pre-frailty undergoing different kinds of elective surgery. Primary endpoint is the care dependency 1 year after surgery [75]. In comparison, PRECOVERY focusses on patients prior to elective cardiac procedure and the efficacy of a 2-week prehabilitation program for patients ≥ 75 years of age. The co-primary outcomes are QoL and mortality 12 months after the cardiac procedure.

The PRECOVERY study design has several strengths. The multicenter alignment enables the establishment of structures that can be further used in the event of a positive evaluation of the new care form “Karl-Heinz.” Furthermore, the 12-month follow-up has the potential to identify long-term effects of prehabilitation prior to the cardiac procedure (e.g., mortality, QoL or cost-effectiveness). Finally, the study analyzes a broad and representative population in cardiac surgery and cardiology. However, this study has several limitations. Firstly, due to the type of the intervention, blinding of patients and various clinical staff is not possible. In PRECOVERY, only the assessment of outcomes is blinded. Secondly, complete standardization of the intervention is not feasible, as modules are selected according to the individual needs of the patients. To standardize the prehabilitation program “Karl-Heinz,” a treatment manual [see Additional file 3] was created in an expert workshop consisting of physicians, psychologists, sports scientists, and physiotherapists. The manual gives information about the intervention framework and how it can be customized to the individual needs of the participants.

In addition to the treatment manual, the exercise programs are based on current study recommendations in combination with recommendations from secondary prevention of treatment in cardiac patients.

This new form of care that aims in particular to further develop intersectoral care funded by the innovation fund of the Federal Joint Committee (G-BA), the highest decision-making body of the joint self-government of physicians, dentists, hospitals, and health insurance funds in Germany can be considered after positive evaluation to be directly incorporated in the German healthcare system in the long term.

### Trial status

The kick-off meeting was held on February 16th, 2023 and the final staff training was conducted on March 16th and 17th, 2023. The first patient was randomized in April 2023. Recruitment will continue until the end of October 2024. Follow-up will be finished by the end of October 2025.

### Supplementary Information

**Additional file file 1.** 

**Additional file file 2.** 

**Additional file file 3.** 

**Additional file file 4.** 

**Additional file file 5.** 

## Data Availability

The University Medical Center Goettingen, Department of Medical Statistics will handle the randomization as well as the data analysis. The healthcare economic evaluation will be performed by University Medical Center Hamburg-Eppendorf, Department of Health Economics and Health Services Research. The study database, monitoring, and safety reporting are operated by the University Medical Center Goettingen, Department of Geriatrics and the Clinical Trials Unit. Regularly, safety reports are generated and distributed to the Data Safety and Monitoring Board. Deidentified datasets can be made available on reasonable scientific request to the consortium management committee after the primary publication. Access might be restricted due to German data protection laws.

## Abbreviations

AE: Adverse event
ATS: American Thoracic Society
BIA: Bioelectrical Impedance Analysis
CSHA: Canadian Study of Health and Aging
DSMB: Data Safety Management Board
eCRF: Electronic case report form
EQ-5D-5L: Euro Quality of Life Questionnaire
FIMA: Fragebogen zur Inanspruchnahme medizinischer und nicht-medizinischer Versorgungsleistungen im Alter (English: Healthcare Resource Utilization by the Elderly])
GCP: Good clinical practice
G-ZBI: German Zarit Burden Interview
HADS: Hospital Anxiety and Depression Scale
HeartQoL: Heart Quality of Life Questionnaire
HFK: Hamburger Fragebogen zum Krankenhausaufhalt (English: Hamburg Hospital Stay Questionnaire)
ICER: Incremental cost-effectiveness
ICU: Intensive care unit
IPQ-B: Illness Perception Questionnaire
IQCODE: Informant Questionnaire on Cognitive Decline in the Elderly
LOT-R: Life-Orientation-Test revised
MD: Mean difference
MNA: Mini Nutritional Assessment
MoCa: Montreal Cognitive Assessment Test
N: Number
PMR: Progressive muscle relaxation
PSQI: Pittsburgh Sleep Quality Index
SMC: Standard Medical Care
RCT: Randomized controlled trial
RR: Risk ratio
UCLA: University of California, Los Angeles
SAE: Serious adverse event
SMI: Self-reported subjective memory impairment
SPPB: Short Physical Performance Battery
SWE: Allgemeine Selbstwirksamkeitsüberzeugung
TEX-Q: Treatment Expectation Questionnaire
