# Supplementary material for: Prehabilitation in older patients prior to elective cardiac procedures (PRECOVERY): study protocol of a multicenter randomized controlled trial
Source: Trials. 2023 Aug 15;24:533. doi: 10.1186/s13063-023-07511-w (PMC10426165; doi:10.1186/s13063-023-07511-w)
Supplement: Supplementary file 1 — Additional file file 1. [file 13063_2023_7511_MOESM1_ESM.pdf]

UNIVERSITÄTSMEDIZIN GÖTTINGEN UMG

# Study protocol

Project title

Prehabilitation "Karl-Heinz" with focus on cardiac and cognitive functions prior to cardiac procedure: an analysis of health status

| | |
|---|---|
| Short title | PRECOVERY |
| Version number | 1.1 |
| Version date | January 10, 2023 |

Department of Geriatrics

University Medical Center Göttingen

Göttingen, January 2023

# 1 Signatures

Study director and biometrician

Göttingen, 05.12.2022

---

Place, date *Signature*

Christine von Arnim, Prof. Dr. med.

(Total) Principal Investigator/ Consortium Management

---

Place, date *Signature*

Thomas Asendorf , Dr.

Person responsible for data management and biometrics

Place, date *Signature*

Monika Sadlonova, Dr. med.

Medical Director/Deputy Principal Investigator

---

Place, date *Signature*

Ingo Kutschka, Prof. Dr. med.

Director of the Department of Cardiac, Thoracic and Vascular Surgery

Person responsible for recruitment in cardiac surgery

---

Place, date *Signature*

Gerd Hasenfuß, Prof. Dr. med.

Director of the Department of Cardiology and Pneumology

Person responsible for recruitment in cardiology

---

Place, date *Signature*

Eva Hummers, Prof. Dr.

Director of the Department of General Medicine

Person responsible for process evaluation

# Table of content


1 Signatures ........ 2
2 Summary of the project ........ 8
3 Responsibilities ........ 16
4 Scientific background ........ 20
5 Study objectives and endpoints ........ 24
6 Study Design and Participating Centers ........ 40
7 Study population ........ 43
8 Curriculum ........ 47
9 Risk-benefit assessment ........ 58
10 Adverse Events (AE) and Serious Adverse Events (SAE) ........ 61
11 Data Safety Monitoring Board (DSMB) ........ 62
12 Monitoring ........ 63
13 Biometrics/Analysis ........ 64
14 Data management and data protection ........ 66
15 Ethical concerns, legal and administrative regulations ........ 70
16 Handling of biosamples ........ 71
17 Subjects' insurance, commuting accident insurance ........ 73
18 Publication rules ........ 74
19 Literaturverzeichnis ........ 75

# Table of Figure

**Figure 1**. PSY-PREP model, modified from Salzmann et al. (2020) 21

**Figure 2**. CONSORT diagram of PRECOVERY randomized controlled trial 38

**Figure 3**. PRECOVERY study design including primary and secondary endpoints 50

**Figure 4**. Data flow between recruiting centers and the PRECOVERY evaluation 63

# List of tables

**Table 1**. Summary of study endpoints and assessments 13

**Table 2**. Summary of the quantitative process evaluation 14

**Table 3**: Summary of the qualitative process evaluation 14

**Table 4**: Study objectives and endpoints 23

**Table 5**. Example of a weekly schedule of prehabilitation intervention 48

**Table 6**. PRECOVERY schedule overview 54

# List of abbreviations

| | |
|---|---|
| Aβ-peptide | abeta-peptide |
| ACTH | adrenocorticotrophic hormone |
| ADL | Activity of daily living |
| AE | Adverse Events |
| AOK | Allgemeine Ortskrankenkasse |
| ApoE genotype | Apolipoprotein E genotyping |
| ASHT | American Society of Hand Therapists |
| ATS | American Thoracic Society |
| BIA | Bioelectrical Impendance Analysis |
| Bzgl. | with regard to |
| Bzw. | or respectively |
| CFS | Clinical Frailty Scale |
| CSHA | Canadian Study of Health and Aging |
| CONSORT | Consolidated Standards of Reporting Trials |
| DMPs | Disease Management Programs |
| DRKS | German Clinical Trials Register |
| eCRF | electronic Case Report Form |

| | |
|---|---|
| ET | Individual Therapy |
| FIMA | questionnaire on the use of medical and non-medical care services in old age |
| GBA | Joint Federal Committee |
| GCP | Good Clinical Practice |
| GDF-15 | Growth-Differentiation Factor 15 |
| Ggf. | If applicable |
| GKV | Statutory health insurance |
| GZB | Zarit Burden Interview |
| GZB-G | Zarit Burden Interview - German Version |
| HADS | Hospital Anxiety and Depression Scale |
| HIF-alpha | Hypoxia-induced factor alpha |
| HOCM | hypertrophic obstructive cardiomyopathy |
| hsTNT | high-sensitive troponin T |
| ICAM | Intercellular adhesion molecule |
| ICD | International Statistical Classification of Diseases |
| IL-1β | interleukin 1β |
| IL1RA | Interleukin-1 receptor antagonist |
| IL-4 | Interleukin 4 |
| IL-6 | Interleukin 6 |
| IL-10 | Interleukin 10 |
| INTERMACS | Interagency Registry for Mechanically Assisted Circulatory Support |
| IPQ | Illness Perception Questionnaire |
| i.S. | in the sense of |
| IQCODE | Informant Questionnaire on Cognitive Decline in the Elderly |
| CHD | Coronary heart disease |
| LOT-R | Life Orientation Test revised |
| MHH | Hanover Medical School |
| MMST | Mini Mental Status Test |
| MNA | Mini Nutritional Assessment |
| MoCa | Montreal Cognitive Assessment Test |
| mod. | modified |
| NfL | Neurofilament light chain |
| NSE | Neuron-specific enolase |

| | |
|---|---|
| NTproBNP | N-terminal pro-brain natriuretic peptide |
| OP | surgery |
| PAVK | Peripheral arterial occlusive disease |
| PSQI | Pittsburgh Sleep Quality Index |
| Q | Quarter |
| QOL | Quality of life |
| QUALYs | Quality-adjusted life years |
| RAID | Redundant Array of Independent Disks |
| RCT | Randomized Controlled Trial |
| SAE | Serious adverse events |
| SAP | Statistical Analysis Plan |
| SDV | Source Data Verification |
| SMI | Self-reported subjective memory impairment |
| SOPs | Standard Operating Procedures |
| SPPB | Short Physical Performance Battery |
| SWE | Self-efficacy expectancy |
| TAVI | Transcatheter Aortic Valve Implantation |
| TEX-Q | Treatment Expectancy Questionnaire |
| TNF-alpha | Tumor necrosis factor-alpha |
| UK | University Hospital |
| ULCA | University of California at Los Angeles |
| UMG | University Medical Center Göttingen |
| UKE | University Medical Center Hamburg-Eppendorf |
| VCAM | Vascular cell adhesion molecules |
| ZAR | Centre for Outpatient Rehabilitation |
| e.g. | for example |
| 6MWT | 6-minute walk test / 6-minute walk test |

# 2 Summary of the project

Recent scientific studies suggest that not only rehabilitation after a medical intervention, but also "prehabilitation" *before* the intervention can have a positive influence on the patient's state of health. Prehabilitation is a treatment concept that improves the physical and mental performance of patients prior to a medical treatment. This randomized, controlled, longitudinal, multicenter, two-arm, assessor-blinded study is designed to investigate the influence of a two-week prehabilitation program on the health status of patients aged 75 years or older and who are undergoing elective cardiac procedure. A total of 422 patients are included. The patients will be randomized 1:1 into the intervention and control groups. Patients in the intervention group will be admitted to a cardiac rehabilitation center for a period of two weeks and receive structured prehabilitation tailored to their individual needs. For patients in the control group no additional interventions outside of the clinical practice routine are planned for patients. Primary endpoints are quality of life (EQ-5D-5L) and mortality one year after the cardiac procedure; secondary endpoints are activities of daily living, cognitive ability and physical performance, disease-related quality of life as well psychological outcomes. An additional component of the randomized study is a detailed health economic analysis. The costs of the intervention, the utilization of health services in the follow-up period and their costs are taken into account, as well the utility values calculated from the results of the EQ-5D-5L. For this part of the project, it is planned to take over routine data from the AOK Lower Saxony and the AOK Baden-Württemberg. Furthermore, a process evaluation takes place in a mixed-methods design. By means of quantitative methods (standardized surveys, patient diaries, therapy plans) the study examines how the quality and implementation of PRECOVERY is influenced by general conditions and resources. Via qualitative methods (e.g., interviews, focus groups), the perspectives of the actors are presented in a differentiated way. The study is funded by the Innovation Fund of the Federal Joint Committee with 5.3 million Euros.

**Synopsis**

| | |
|---|---|
| Title | Prehabilitation "Karl-Heinz" with a focus on cardiac and cognitive functions prior to cardiac procedure: a health status analysis |
| Short title | PRECOVERY |
| UMG registration number | 2020-01057 |
| DRKS | DRKS00030526 |
| Subject area | Integration and networking of rehabilitative measures to increase the treatment success of SHI (statutory health insurance) services |

| | |
|---|---|
| Project goals | Prehabilitation prior to cardiac procedure in the elderly (≥75 years) at 12 months leads to:<br>1) Improvement in quality of life/1-year mortality,<br>2) Improvement in daily function, heart-specific quality of life, physical and mental performance as well as<br>3) Reduction of associated health care costs compared to the control group. |
| Inclusion criteria | 1. Patients ≥75 years of age who are scheduled for one of the following elective cardiac procedures: (a) replacement of heart valves by prosthesis (5-351), (b) exchange of prosthetic heart valves (5-352), c) Valvuloplasty (5-353), d) Other operations on heart valves (5-354), e) Minimally invasive operations on heart valves (incl. catheter-assisted aortic valve implantation - TAVI and transvenous clip reconstruction of the mitral valve - MitraClip) (5-35a), f) Desobliteration (endarterectomy) of the coronary arteries (5-360), g) Placement of an aortocoronary bypass (5-361), h) Placement of an aortocoronary bypass by minimally invasive technique (5-362) or i) Other revascularization of the heart (5-363).<br>2. Sufficient independence and ability to care for oneself to participate in prehabilitation<br>3. Insurance status of all patients from Lower Saxony with the AOK<br>4. Ability to give consent<br>5. Willingness to participate in the study on a voluntary basis with signed informed consent form<br>6. Sufficient knowledge of the German language to fill in/answer the questionnaires |
| Exclusion criteria | 1. Lack of capacity to consent<br>2. Katz index 0<br>3. Need for treatment in an acute care hospital<br>4. Severe dementia; severe mental disorders (acute psychoses, severe depressive episode, acute suicidality), acute delirium<br>5. Diagnosis of acute alcohol or drug abuse<br>6. Unstable angina pectoris<br>7. Heart failure, NYHA IV<br>8. Myocarditis, HOCM, main stem stenosis ≥ 80%.<br>9. Severe refractory cardiac arrhythmias<br>10. Post aortic dissection<br>11. PAVK stage ≥ III according to Fontaine<br>12. Symptomatic carotid stenosis or carotid stenosis requiring treatment<br>13. Renal insufficiency requiring dialysis<br>14. Hepatic insufficiency, Child B and Child C<br>15. Advanced (metastatic) oncological disease<br>16. Neurological, orthopedic or rheumatic impairing co-morbidities that militate against physical training<br>17. Participation in another intervention study (participation in registry studies is allowed) |
| Endpoints | <u>Primary endpoint:</u> EQ-5D-5L (general quality of life) and mortality 12 months after cardiac procedure |

| | |
|---|---|
| | Secondary endpoint:<br>Katz index (daily living function), HeartQoL (disease-related quality of life), Short Physical Performance Battery (SPPB, physical performance) and MoCa (mental performance), HADS (assessment of anxiety and depression)<br><br>Other endpoints:<br>30-day mortality, hand strength measurement (frailty, malnutrition), 6-minute walk test (functional capacity), Bioelectrical analysis (body composition), Mini Nutritional Assessment (MNA, malnutrition), Pittsburgh Sleep Quality Index (PSQI, sleep quality), Maastricht Questionnaire (vital fatigue), Informant Questionnaire on Cognitive Decline in the Elderly (IQCODE, assessment of family members), Zarit Burden Interview (GZBI, assessment of the burden on family members), Clinical Frailty Scale (CSHA Frailty Index, frailty), ATS Scale (Dyspnoea scale of the American Thoracic Society), self-reported subjective memory impairment (SMI), length of stay in intensive care and normal ward, peri- and post-operative/interventional complications, AE/SAE-assessments, SWE scale (self-efficacy expectation), Hamburg Hospitalization Questionnaire (patient satisfaction), optimism (Life Orientation Test revised - LOT-R, self-efficacy), loneliness (ULCA Loneliness Scale), Illness Perception Questionnaire (IPQ), Treatment Expectancy (TEX-Q), Institutionalization/Dependence on Care<br><br>Health economic endpoints:<br>Intervention costs, utilization and costs during follow-up, determination of utility values (quality-weighted life years, EQ-5D-5L), cost-effectiveness, questionnaire on the use of medical and non-medical care services in old age (FIMA questionnaire, routine data of the AOK).<br><br>Process evaluation:<br>A mixed-methods process evaluation will be carried out, using quantitative methods (standardized surveys, patient diaries, therapy plans) to examine how the quality and implementation of PRECOVERY are influenced by the framework conditions and resources, with qualitative methods (interviews, focus groups) will be used to differentiate the perspectives of the actors. |
| Study design | Randomized, controlled, longitudinal, multicenter, 2-arm, assessor-blinded study. This will be accompanied by a mixed methods process evaluation. |
| Statistical Analysis | Clinical endpoints:<br>The evaluation of the primary endpoint EQ-5D-5L is performed using a joint model for longitudinal data. Stratification factors age, gender, participation in DMPs and center are included as covariates in the model. Time courses of the EQ-5D-5L are calculated longitudinally for each group and survival rates are survival rates with the help of Kaplan-Meier curves. Treatment effects will be reported with 95% confidence intervals. |

| | |
|---|---|
| | The secondary endpoint HeartQoL will be evaluated analogously to the primary endpoint. Secondary endpoints Katz index, SPPB, MoCa and HADS are evaluated using mixed linear models, with the factors time point, treatment and the interaction as well as the covariates age, gender, DMP participation and center.<br>Marginal means are calculated for inter- and intra-group comparisons and treatment effects are reported with 95% confidence intervals. The other endpoints are also evaluated using generalized mixed linear models, depending on the data type.<br><br>Health economic outcomes:<br>Mixed regression models are used to test for differences in means. Due to the skewed distribution of the cost data the standard errors of the regressors, the non-parametric bootstrapping method is used, which is also used in the uncertainty analysis of the IKER. For the analysis of cost-effectiveness, the net-benefit regression method is used.<br><br>Evaluation of the mixed-methods process evaluation:<br>Quantitative data are evaluated descriptively and qualitative data are analyzed using content analysis according to Kuckartz. Both parts of the evaluation are then merged. |
| Sample size | n=422 patients (211 intervention / 211 control group) |
| Duration of studies | Total running time: — 01.11.22 – 31.10.26 48 month<br>Recruitment period ("first patient in" to "last patient out"): — 29 month<br>Treatment duration per patient ("first patient in" to "first patient out"): — 14 month<br>"Follow up" pro Patient: — 30 days (t4), 6 month (t5) and 12 month (t6) post-operative |
| Planned times | Inclusion of the 1st patient. "First patient in“ — Q2 2023<br>Inclusion of the last patient "Last patient in" — Q2 2024<br>End of study "Last patient out“ — Q3 2025<br>Final statistical analysis — Q1 2026<br>Planned interim analysis — After 25% of the recruited patients |
| Participating locations | Recruitment centers: Clinic for Cardiac, Thoracic and Vascular Surgery, UMG Göttingen; Clinic for Cardiology and Pneumology, UMG Göttingen; Clinic for Thoracic, Cardiac and Vascular Surgery, MHH Hannover; Clinic for Thoracic, Cardiac and Vascular Surgery, UK Ulm, Clinic for Thoracic, Cardiac and Vascular Surgery, Braunschweig, UK for Cardiac Surgery, Klinikum Oldenburg, Heart Center Brandenburg, Immanuel Klinikum Bernau, Bernau Waldsiedlung |

| | |
|---|---|
| | <u>Recruitment and prehabilitation centers:</u> Schüchtermann-Schiller'sche Kliniken, Bad Rothenfelde, Herz- und Gefäßzentrum, Bad Bevensen<br><br><u>Prehabilitation centers:</u> Oldenburg Rehabilitation Center, Lippoldsberg Clinic and Rehabilitation Centre, Kirchberg Clinic, Bad Lauterberg, ZAR Center for Outpatient Rehabilitation GmbH am Main.<br>Lauterberg, ZAR Centre for Outpatient Rehabilitation GmbH at the UK Ulm, Klinik Fallingbostel, Bad Fallingbostel, Brandenburg Klinik |
| Third-party funder | Innovation Fund of the Federal Joint Committee |

Table 1. Summary of study endpoints and assessments

| **Study Period** | Screening, enrollment, allocation, assessments prior to cardiac procedure | | | | | Assessments post cardiac procedure | | | |
|---|---|---|---|---|---|---|---|---|---|
| **Duration** | 4-6 weeks | | | | | 12 months | | | |
| **Timepoint** | | t0/t1 | **Prehabilitation „Karl-Heinz" (2 weeks, N=211) <u>or</u> Standard Medical Care (SMC, N=211)** | t2 | **Cardiac procedure (N=422)** | t3 | t4 (phone) | t5 (phone) | t6 |
| | Screening for eligibility | Baseline assessment/ Randomization | | post-prehabilitation (N=211) or post-SMC (N=211), prior to cardiac procedure (N=422) | | post cardiac procedure, before hospital discharge | 30 days post cardiac procedrue | 6 months post cardiac procedure | 12 months post cardiac procedure |
| **Enrollment:** | | | | | | | | | |
| Inclusion/Exclusion criteria | x | | | | | | | | |
| Informed consent, enrollment, allocation | | x | | | | | | | |
| Blood sample | | x | | | | | | | |
| Medical history, social demographics | | x | | | | | | | |
| **Assessments:** | | | | | | | | | |
| EQ-5D-5L (QoL) | | x | | x | | x | x | x | x |
| 1-year mortality | | | | | | | | | x |
| Katz-index (daily activity) | | x | | x | | x | x | x | x |
| SPPB (physical performance) | | x | | x | | | | | x |
| MoCa (mental performance) | | x | | x | | | | | x |
| HeartQoL (disease-specific QoL) | | x | | x | | | x | x | x |
| HADS (anxiety and depression) | | x | | x | | | x | x | x |
| 30-day-mortality | | | | | | | x | | |
| Hand grip strength (frailty) | | x | | x | | | | | x |
| 6-minute walk test (functional capacity) | | x | | x | | | | | x |
| BIA (body composition) | | x | | x | | | | | x |
| MNA (frailty, malnutrition) | | x | | x | | | | | x |
| PSQI (sleep quality) | | x | | x | | | | | x |
| Maastricht questionnaire (vital exhaustion) | | x | | x | | | x | | x |
| UCLA Loneliness Scale (loneliness) | | x | | | | | x | | x |
| SMI (memory impairment) | | x | | x | | | x | x | x |
| CSHA-Frailty-Index (frailty) | | x | | x | | x | | | x |
| Pre- and post-interventional complications | | | | | | x | | | |
| Length of stay in intensive and normal care | | | | | | x | | | |
| Institutionalization/dependence on care | | x | | x | | x | x | x | x |
| ATS scale (dyspnoe) | | x | | x | | x | x | x | x |
| SWE scale (self-efficacy expectancy) | | x | | | | | x | x | x |
| HFK (patients‘ satisfaction) | | | | | | x | | | |
| LOT-R (optimism) | | x | | x | | | | | |
| IPQ-B (illness perception) | | x | | x | | | | | x |
| TEX-Q (treatment expectancy) | | x | | x | | | | | |
| Process evaluation | | x | | x | | x | x | x | x |
| FIMA Questionnaire (healthcare-related resource utilization) | | x | | | | | | x | x |
| Blood sample | | x | | x | | x | | | x |
| AE/SAE | | x | | x | | x | x | x | x |
| **Relatives:** G-ZBI (burden of relatives) | | x | | | | | | x | x |
| **Relatives:** IQCODE (opinion of relatives) | | x | | | | | | x | x |

Abbreviations. EQ-5D-5L: Euro Quality of Life Questionnaire; SPPB: Short Physical Performance Battery; MoCa: Montreal Cognitive Assessment Test; HeartQoL: Heart Quality of Life Questionnaire; HADS: Hospital Anxiety and Depression Scale; BIA: Bioelectrical Impendance Analysis; MNA: Mini Nutritional Assessment; PSQI: Pittsburgh Sleep Quality Index; UCLA: University of California, Los Angeles; SMI: self-reported subjective memory impairment; CSHA: Canadian Study of Health and Aging; ATS: American Thoracic Society; SWE: Allgemeine Selbstwirksamkeitsüberzeugung; HFK: Hamburger Fragebogen zum Krankenhausaufhalt (English: Hamburg Hospital Stay Questionnaire); LOT-R: Life-Orientation-Test revised; IPQ-B: Illness Perception Questionnaire; TEX-Q: Treatment Expectation Questionnaire; FIMA: Fragebogen zur Inanspruchnahme medizinischer und nicht-medizinischer Versorgungsleistungen im Alter (English: Healthcare Resource Utilization by the Elderly]); AE: adverse event; SAE: serious adverse event; G-ZBI: German Zarit Burden Interview; IQCODE: Informant Questionnaire on Cognitive Decline in the Elderly; N: number; SMC: Standard Medical Care.

**Table 2**. Summary of the quantitative process evaluation

| | **Training** (on site & TtT*) before prehabilitation | **T0/T1** before prehabili-tation | **T2** pre-op, post prehabilita-tion | **T3** post-op | **T4** 30 d post-op | **T5** 6 m post-op | **T 6** 12 m post-op |
|---|---|---|---|---|---|---|---|
| **Patients IG** | | **x** | **x** | **x** | **x** | **x** | **x** |
| **Patients CG** | | **x** | | **x** | **x** | **x** | **x** |
| **Relatives IG** | | **x** | | | | **x** | **x** |
| **Relatives CG** | | **x** | | | | **x** | **x** |
| **Patients IG** | | **patient diaries (during prehabilitation)** | | | | | |
| **Facility managers** | | **x** | **x** | | | | |
| **Multipliers** | **x** | **x x x** | | | | | |
| **Multipliers** | | **therapy plans** | | | | | |
| **Therapists, nurses, doctors at prehabilitation centers** | **x** | **x x x** | | | | | |
| **Change of multiplier or staff at rehabilitation centers** | | **continuously during prehabilitation period** | | | | | |
| **Staff in study center** | **x** | | | | | | |

Abbreviations. IG = intervention group, CG = control group

**Table 3**. Summary of the qualitative process evaluation

| | **T1** before prehabilitatio n | **T2** post prehabilitatio n | **T4** 30 d post-op | **T6** 12 m post-op | after prehabilitation period |
|---|---|---|---|---|---|
| **Patients IG** | **x** | **x** | **x** | **x** | |
| **Patients CG** | **x** | | **x** | **x** | |
| **Relatives IG** | | | **x** | **x** | |
| **Relatives CG** | | | **x** | **x** | |
| **Multipliers** | | | | | **x** |
| **Therapists, nurses, doctors at prehabilitation centers** | | | | | **x** |
| **General practitioners of patients in the IG** | | | | **x (after last patient)** | |
| **Doctors in recruitment centers** | | | | **x (after last patient)** | |

Abbreviations. IG: intervention group, CG: control group

# 3 Responsibilities

### Study leader

Prof. Dr. Christine von Arnim, Director of the Department of Geriatrics at the University Medical Centre Göttingen, leads the PRECOVERY consortium.

| Name | Institution | Phone, Fax, Mail | Responsibility |
|---|---|---|---|
| Prof. Dr. Christine v. Arnim | Department of Geriatrics, University of Goettingen Medical Center (UMG) | 0551-39-62149; -62615 christine.arnim@med.uni-goettingen.de | Project management Consortium management |

### Participating scientists

| Name | Institution | Phone, Fax, Mail | Responsibility |
|---|---|---|---|
| Dr. Monika Sadlonova | Department of Cardiovascular and Thoracic Surgery, Department of Geriatrics, Department of Psychosomatic Medicine and Psychotherapy, UMG | 0551-39-66001; -66002 monika.sadlonova@med.uni-goettingen.de | Medical Project Management Deputy Head of Communication with Recruiting Centers Investigator Supervision trainings |
| Dr. Stephanie Heinemann | Department of Geriatrics, UMG | 0551 39 65391 stephanie.heinemann@med.uni-goettingen.de | Communication Contractual partners, contracts |
| Dr. Carolin Steinmetz | Department of Geriatrics, UMG | carolin.steinmetz@med.uni-goettingen.de | Treatment manual, communication preha centers |
| Dr. Simone Krieger | Department of Psychosomatic Medicine and Psychotherapy, UMG | simone.krieger@med.uni-goettingen.de | Treatment manual, trainings |
| Dr. Thomas Asendorf | Department of Medical Statistics, UMG | 0551-39-63026; -4995 thomas.asendorf@med.uni-goettingen.de | Study design and biometry |
| Marianne Richter | Studienzentrum, UMG | marianne.richter@med-uni-goettingen.de | Datamanagement |
| Prof. Dr. Tim Mathes | Department of Medical Statistics, UMG | tim.mathes@med.uni-goettingen.de | Process evaluation |
| Prof. Dr. Hans-Helmut König, M.P.H | Department for Health Economics and Health Services Research, University Medical Center Hamburg-Eppendorf (UKE) | 040 7410 – 54464; -40261 h.koenig@uke.de | Health economics |
| Prof. Dr. Eva Hummers Dr. Christiane Müller, MPH Annemieke Munderloh | Department of General Practice, UMG | 0551-39-22638; -9530 eva.hummers@med.uni-goettingen.de | Process evaluation |

| | | | |
|---|---|---|---|
| Prof. Dr. M. Denkinger, PD Dr. D. Dallmeier | Geriatric Center, Agaplesion Bethesda Ulm | 0731-187-185; -300 michael.denkinger@bethesda-ulm.de | Assessmenttrainings |
| Prof. Dr. Christoph Herrmann-Lingen | Department of Psychosomatic Medicine and Psychotherapy, UMG | 0551-39-66707; -19150 cherma@gwdg.de | Psychological Intervention |
| Jun. Prof. Dr. Thomas Schmidt | German Sport University, Cologne | 0221 4982-5075 t.schmidt@dshs-koeln.de | Cooperation Partner |

**Recruitment and Prehabilitation facilities involved**

| Name | Institution | Phone, Fax, Mail | Responsibility |
|---|---|---|---|
| Prof. Dr. Ingo Kutschka | Department of Cardiovascular and Thoracic Surgery, UMG | 0551-39-66001; -66002; ingo.kutschka@med.uni-goettingen.de | Recruitment Center |
| Prof. Dr. Gerd Hasenfuß | Department of Cardiology and Pneumology, UMG | 0551-39-20400; - 66389 hasenfus@med.uni-goettingen.de | Recruitment Center |
| Prof. Dr. Andreas Martens | Department of Cardiothoracic, Transplantation and Vascular Surgery, Hannover Medical School (MHH) | 0511-5326581; -5404 martens.andreas@mh-hannover.de | Recruitment Center |
| Prof. Dr. Andreas Liebold | Clinic for thoracic, cardiac and Vascular Surgery, UK Ulm | 0731-500-54303; -54304 andreas.liebold@uniklinik-ulm.d | Recruitment Center |
| PD Dr. Wolfgang Harringer | Department of Cardiac, Thoracic and Vascular Surgery, Braunschweig Municipal Hospital | 0531-595-2213; - 2658 w.harringer@klinikum-braunschweig.de | Recruitment Center |
| PD Dr. Friedrich Mellert | University Clinic for Cardiac Surgery, Oldenburg Clinic | 0441-403-2820; -2830 herzchirurgie@klinikum-oldenburg.de | Recruitment Center |
| Prof. Dr. Johannes Maximilian Albes | Brandenburg Heart Center, Immanuel Clinic, Bernau | 0333-869-4510; -4544 johannes.albes@mhb-fontane.de | Recruitment Center |
| Prof. Dr. Nils Reiss | Schüchtermann-Schiller'sche Clinic, Bad Rothenfelde | 05424641-30269; 05424641-206 NReiss@schuechtermann-klinik.de | Recruitment and Rehabilitation Center |
| Prof. Dr. DB. A. Remppis, Dr. Christian Baumbach | Heart and Vascular Center, Bad Bevensen | 05821 82-1703; -82888 ba.remppis@hgz-bb.de | Recruitment and Rehabilitation Center |
| Dr. med. Daniela Heidkamp | Rehabilitation Center Oldenburg | 0441405-2447 heidkamp.daniela@reha-ol.de | Prehabilitation Center |
| Dr. Michael Don, Prof. Dr. G. Hasenfuß, Ralf Pinnau | Clinic and Rehabilitation Center Lippoldsberg | 0557-241-1202; -1701 rpinnau@aol.com | Prehabilitation Center |

| | | | |
|---|---|---|---|
| Dr. Ernst Knoglinger | Kirchberg-Clinic, Bad Lauterberg | 05524 / 859253; -859766 knoglinger@kirchbergklinik.de | Prehabilitation Center |
| Susann Ernst | ZAR Center for Outpatient Rehabilitation GmbH at UKU | 0152/ 05752513 lauer@zar-ulm.de | Prehabilitation Center |
| Dr. Susanne Brunner | Clinic Fallingbostel, Bad, Fallingbostel | 05162 / 44-701; 44-88701 brunner@klinik-fallingbostel.de | Prehabilitation Center |
| Dr. Martin Schikora | Brandenburg Clinic, Bernau, Waldsiedlung | 033397- 3-1400; -33379 schikora@brandenburgklinik.de | Prehabilitation Center |

### Involved cooperation partners

| Name | Institution | Phone, Fax, Mail | Responsibility |
|---|---|---|---|
| Markus Matzeder<br>Dr. Sarah Rust | AOK Lower Saxony | 0511 – 285 13212; 0511 – 285 13108, Marcus.Matzeder@nds.aok.de Sarah.Rust@nds.aok.de | consortium partners |
| Christine Kleber-Peukert | AOK Baden-Württemberg, GB healthcare innovations & cross-sectoral utility solutions | 0711 6525-17719 Christine.Kleber-Peukert@bw.aok.de | consortium partners |
| Martin Vestweber | German Heart Foundation | 069 955128-121; vestweber@herzstiftung.de | consortium partners |
| Jun.-Prof. Dr. Thomas Schmidt | German Sport University Cologne | 0221 4982-5075 t.schmidt@dshs-koeln.de | consortium partners |

### Data Safety Monitoring Board (DSMB) involved

| Name | Institution | Phone, Fax, Mail | Responsibility |
|---|---|---|---|
| Prof. Dr. Andreas Böning | Clinic for cardiac, pediatric cardiac and vascular surgery University Hospital Gießen and Marburg | andreas.boening@chiru.med.uni-giessen.de, 0641-985 44301 | DSMB Bereich Herzchirurgie |
| PD Dr. Claudia Walther | MVC CCB at Agaplesion bethany hospital, cardiovascular center, Frankfurt am Main | c.walther@ccb.de, 069 9450280 | DSMB Bereich Kardiologie |
| Prof. Dr. Clemens Becker | Robert Bosch Hospital, geriatric research and geriatric rehabilitation | clemens.becker@rbk.de, 0711 8101 3101 | DSMB area geriatrics |
| Prof. Dr. Julie Silver | Harvard Medical School, Department of Physical medicine and rehabilitation | julie_silver@hms.harvard.edu, +1 866-378-9164 | DSMB area of pre-habilitation |

| | | | |
|---|---|---|---|
| Prof. Dr. Thomas Mengden | Kerckhoff Rehabilitation Center Bad Nauheim | t.mengden@reha.kerckhoff-klinik.de, 06032 999 5906 | DSMB Rehabilitation Division |
| Frau Christine Dehn | German Heart Foundation | dehn@herzstiftung.de, 069 955128 161 | DSMB area patient representation |

**Financing**

The study is funded by the Innovation Fund of the Federal Joint Committee financed by 5.3 million euros under grant number 01NVF21109. The blood draws are not funded externally.

**Registration**

The study was registered in the German Register of Clinical Studies (DRKS) under the number DRKS00030526.

# 4 Scientific background

According to the Federal Statistical Office (2016) around 13.6 million people in Germany reach the age of over 75 years. Until 2030, hospitalizations for older patients are expected to increase by approximately 30% (Federal Statistical Office 2016). Within this demographic, the prevalence and incidence of cardiovascular diseases (e.g., coronary heart disease [CHD] or degenerative-calcifying aortic valve stenosis) will increase.

According to the heart report, 37.984 isolated and combined coronary artery bypass graft (CABG) surgeries were carried out in Germany in 2020 (German Heart Foundation 2022). More than 40 % of the CABG patients were aged 70 years or older (German Heart Foundation 2022). The indication for CABG is the complexity of the coronary findings (e.g., severe triple-vessel CHD and/or high-grade blockages in any of the major coronary arteries if the percutaneous coronary intervention has failed to treat the blockages) in dependence on comorbidities (e.g., diabetes), and the patient's treatment preferences (Neumann et al. 2019). The in-hospital mortality for isolated CABG surgeries is in Germany under 3% (German Heart Foundation 2022). The increasing incidence of heart valve diseases (especially of the aortic and mitral valve) by elderly patients, the increasing life expectancy of the population and the associated increase in morbidity and mortality are becoming major cardiac health problems. In 2020, 17.998 isolated or combined conventional surgical heart valve surgeries (including 8.049 isolated aortic valve procedures, 6.050 isolated mitral valve procedures) were performed. The transcatheter aortic valve implantation (TAVI) has been the preferred treatment for patients aged 75 years or older with a severe aortic stenosis. In Germany, 21.544 TAVIs were performed in 2020. In primary (degenerative) mitral valve insufficiency, the European treatment guidelines from 2021 recommend catheter-based treatment options (Vahanian et al. 2021). In 2020, a total of 6.011 interventional mitral valve procedures with mean age of the treated patients of 78 years were performed in Germany.

Surgical and catheter-based cardiac procedures have been successful treatment alternatives with increased safety. However, the risk of cardiac procedures in elderly, multimorbid patients is still very high and is often associated with prolonged convalescence. For example, postoperative/postinterventional delirium occurs in 12-53% of cardiac patients with poor outcomes (e.g., prolonged hospitalization, impaired cognition, less independence in everyday life and increased risk of mortality) (van der Wulp et al. 2019).

Furthermore, delirium can be associated with an increased risk of postoperative cognitive dysfunction and dementia (Fong et al. 2015; Fuchs et al. 2020; Witlox et al. 2010; Vasunilashorn et al. 2018). The 1-year mortality is assumed to be around 15-20% and increases with age (Velazquez et al. 2016; McIsaac et al. 2016; Blumenstein et al. 2020). In delirious patients with frailty, mortality increases up to 40% (Kundi et al. 2019; Puls et al. 2014).

Surgical procedures, particularly heart surgeries, have been shown to increase markers of neuronal damage and neurodegeneration, such as total tau and neurofilament light chains (NfL) (Alifier et al. 2020; Chan et al. 2021; DiMeglio et al. 2019; Evered et al. 2018). These pathophysiological effects, including hypoxemia and disruption of the blood-brain barrier, are not only caused by anesthesia as previously assumed (Deiner et al. 2020). The use of the extracorporeal circulation on the heart-lung machine might be a relevant cause of neuronal damage. It is likely that patients with pre-existing risk factors (e.g., an underlying neurodegenerative disorder) may experience a significant 'second hit' that can accelerate neurodegenerative processes. This hypothesis can be supported by preoperative and postoperative measurements of important neurodegenerative (e.g., total tau, NfL), inflammatory (e.g., C-reactive protein, interleukins), and cardiac (e.g., GDF-15) biomarkers. In addition, patients often have to wait several weeks for an elective cardiac procedure which is associated with psychological stress and reduced functionality (Sampalis et al. 2001). Furthermore, prior to a cardiac procedure, there is a high prevalence of anxiety and depression that negatively affect the peri- and postoperative healing processes (Hoyer et al. 2008). This is of clinical relevance since the symptoms of depression and anxiety often persist for months after cardiac procedures (Doering et al. 2014). In addition, negative disease expectations can negatively affect morbidity and mortality in patients with CHD (Barefoot et al. 2011). A number of studies have shown that negative patient expectations before CABG or related surgeries can predict impairments in quality of life (Juergens et al. 2010), postoperative depression and anxiety, as well as a negative mental state (Auer et al. 2016; Rimington et al. 2010). The reduction of anxiety, depression, disease assumptions and treatment expectations as well as an optimal transfer of information and preparation of coping strategies represent important starting points for preoperative psychological and educational interventions (Rief et al. 2017; Salzmann et al. 2020; Howard Leventhal et al. 2020). This content is shown in Figure 1.

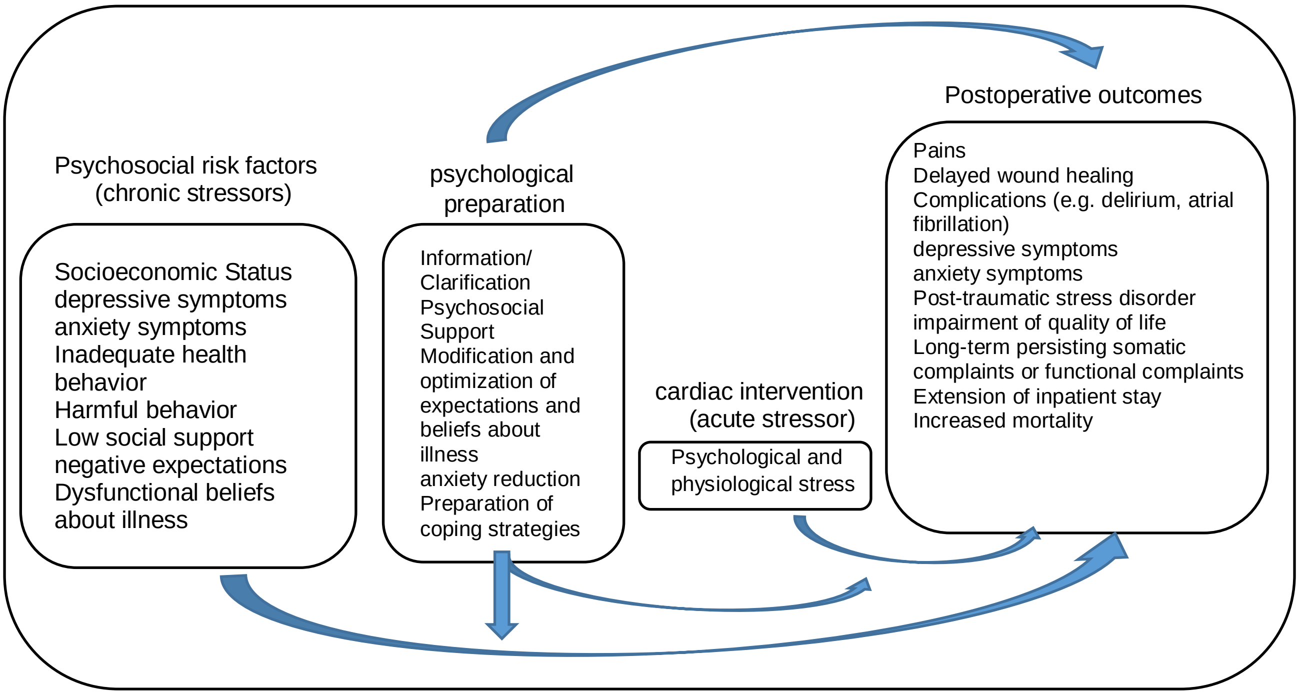


**Figure 1**. PSY-PREP model modified from Salzmann et al. (2020)

The evidence for the effectiveness of phase II rehabilitation for patients after cardiac procedures is well established. Especially elderly and multimorbid patients seem to benefit the most from cardiac rehabilitation (Spiroski et al. 2017; Vanhees et al. 2004; Busch et al. 2012; Scrutinio and Giannuzzi 2008; Bjarnason-Wehrens et al. 2007). Primary rehabilitation goals of this patient group are the improvements of physical performance, physical capacity, participation in everyday life, and avoidance of the need for long-term care (Eder et al. 2013). In order to achieve the above-mentioned goals in the best possible way, prehabilitation before elective cardiac procedures is becoming more and more important. The main idea of prehabilitation is to improve the physical and mental performance of the patient before a planned treatment to achieve the highest possible level of functional ability and recovery (Cabilan et al. 2016; Punt et al. 2017; Topp et al. 2002).

Frailty describes the geriatric syndrome including the following three main components: loss of muscle mass, muscle strength and muscle endurance (Fried et al. 2001). Frail patients have a threefold increase in postoperative morbidity and mortality (Afilalo et al. 2010). Furthermore, the risk of cerebrovascular or cardiac events during or after cardiac surgery is significantly increased in patients with frailty (Sepehri et al. 2014). Particularly in elderly and frail patients, reduction or loss of physical functioning before, during and after hospitalization may have a strong negative impact on postoperative/postinterventional outcomes (Hulzebos and van Meeteren 2016).

Recent reviews show that not only rehabilitation, but especially preparation for cardiac procedures in the context of prehabilitation can improve perioperative/peri-interventional outcomes and alleviate negative effects. Participation in prehabilitation prior to elective cardiac

surgery was associated with both preoperative and postoperative improvements in functional capacity as measured by 6-minute walk distance, reduced extubation time, reduced risk of postoperative pulmonary complications and reduced hospitalization (Steinmetz et al. 2022; Yau et al. 2021; Marmelo et al. 2018; Snowdon et al. 2014; Hulzebos and van Meeteren 2016). Further randomized controlled trials on cardiac prehabilitation show positive effects on delirium rates, quality of life, length of stay in the intensive care unit and improved compliance with postoperative rehabilitation (McCann et al. 2019; Hall et al. 2017). Pre- and perioperative psychological interventions in cardiac surgery also led to a reduction in length of stay and a significant increase in self-efficacy (Sadlonova et al. 2022; Auer et al. 2017).

The innovative concept of prehabilitation was developed to increase the functional capacity of patients in the perioperative phase and to reduce postoperative morbidity. Despite excellent data from clinical studies and the increasing number of cardiac interventions in older people, there is no structured cardiac prehabilitation program that is tailored to the needs and requirements of this group of patients and which is coordinated across the sectors involved in the health care system. This project aims to close this supply gap.

# 5 Study objectives and endpoints

**Table 4**. Study objectives and endpoints

| | Study objective | Endpoint |
|---|---|---|
| **Primary** | Evidence that the two-week Karl-Heinz" prehabilitation program before a planned cardiac procedure in patients aged ≥ 75 years compared to standard medical care (SMC) increases the heath status and reduces mortality in patients 12 months after the cardiac procedure. | EQ-5D-5L and mortality 12 months after a cardiac procedure |
| **Secondary** | Evidence that the two-week Karl-Heinz" prehabilitation program before a planned cardiac procedure in patients aged ≥75 years compared to SMC, improves daily function, quality of life, physical and mental performance and mental state. | Katz-Index<br>HeartQoL<br>SPPB<br>MoCa<br>HADS |
| **Further Study objectives and Endpoints** | Evidence that the two-week prehabilitation program "Karl-Heinz" prior to planned cardiac procedure in patients aged ≥75 years compared to SMC:<br>- 30-day mortality<br>- hand strength<br>- 6-minute walking distance<br>- body composition<br>- sleep quality<br>- vital state of exhaustion<br>- frailty<br>- shortness of breath<br>- length of hospital stay<br>- institutionalization/need for care<br>- self-efficacy<br>- subjective memory<br>- optimism<br>- loneliness<br>- acceptance of illness and | 30-day mortality<br>Hand strength measurement<br>6-minute walk test<br>BIA<br>Pittsburgh Sleep Quality Index<br>Maastricht Questionnaire<br>Clinical Frailty Scale<br>ATS Scale<br>Length of stay intensive and normal ward<br>Documentation of institutionalization and need for care<br>SWE scale<br>Self-reported subjective memory impairment<br>Life Orientation Test revised<br>ULCA<br>Illness Perception Questionnaire |

| | | |
|---|---|---|
| | - the expectation of treatment<br>Other goals of the<br>Prehabilitation program are:<br>- To detect and counteract preoperative malnutrition<br>- Reduction of peri-interventional/operative complications<br>- Improvement of patient satisfaction<br>- Education of relatives<br>- Changes in blood-based biomarkers (e.g., neurodegenerative, inflammatory and cardiovascular markers). | Treatment Expectation<br>Questionnaire<br>Mini Nutritional Assessment<br>Peri- and<br>post-interventional/surgical<br>complications, such as<br>Post-interventional delirium,<br>pneumonia, atrial fibrillation,<br>Pleural/pericardial effusion,<br>Pneumothorax, death<br>AE/SAE Assessment<br>Hamburg questionnaire on<br>hospitalization<br>IQCODE<br>G-ZBI<br>Determination of blood-based<br>factors (proteinomic,<br>metabolomic, genetic<br>and epigenetic), which are<br>positive course or<br>predict or modify complications. |
| **Health Economics Analysis** | Evidence that the structured prehabilitation "Karl-Heinz" leads to a reduction in associated health care costs for the SHI system and the hospital providers compared to the SMC. | - Costs of the intervention<br>- Utilization of<br>health services and<br>Costs during follow-up (FIMA and routine SHI data of the AOK from the areas of<br>outpatient<br>and inpatient care, medicines, remedies<br>and<br>remedies, rehabilitation,<br>care, DMP and HzV)<br>- Determination of utility values (quality-weighted life years - QALYs)<br>- Cost-effectiveness |
| **Process evaluation** | <u>Quantitative process evaluation:</u><br>Investigation of implementation strategies, and their implementation as well as the framework conditions,<br>Evaluation of the trainings and their implementation. | Program fidelity<br>Dose<br>Adjustments<br>Range<br>Influence of framework conditions<br>View of the implementation of the intervention as well as effects, reactions |

| | | |
|---|---|---|
| | Qualitative process evaluation:<br>Exploring expectations, experiences and perceived benefits of Karl-Heinz by those directly involved in the implementation (patients, related persons, multipliers, staff, and family physicians). Recording of influencing factors | and adjustments on the part of the participants and implementers<br>Evaluation of the trainings<br><br>No endpoints in qualitative research. |

**Clinical information, sociodemographics and protocol of cardiac intervention:**

The medical history includes age, height, weight, hip and waist circumference, blood pressure, pulse, cardiovascular risk factors such as diabetes mellitus, hypertension, peripheral occlusive disease, renal insufficiency, dyslipidemia, smoking, alcohol abuse as well as other cardiac and non-cardiac diseases. The previous cardiac and vascular surgical procedures are also documented. Socio-demographic data are included in the database in the form of marital status and level of education are included in the database. Routine laboratory measurements before and after the cardiac procedure (e.g., albumin, CRP, electrolytes, transaminases and others) from the IXSERV or ICCA (in the cardiac surgery intensive care unit) are transferred to a standardized laboratory protocol. Furthermore, we will provide a cardiac surgical and interventional protocol (including intraprocedural complications) with detailed information about the cardiac procedure. The total length of the inpatient stay, the length of stay in the intensive care unit and the performance and length of cardiac rehabilitation are also recorded. Before and after the cardiac procedure, the occurrence of dyspnea is also documented. Postoperative/postinterventional complications (e.g., pleural effusions, pneumothorax, cardiac arrhythmias) are also systematically recorded. In order not to miss the occurrence of delirium, the patient's medical records will be reviewed. Here, particular attention is paid to the nursing notes, especially during night shifts (chart review). If there are signs of severe agitation or other delirious symptoms according to ICD-10 diagnostic criteria as well as a prescription of medication for treatment of delirium or agitation (e.g., haloperidol), the patient is classified as delirious.

The endpoints are collected using the following validated assessments:

**Euro Quality of Life (EQ-5D-5L)**

The five-dimensional EuroQoL questionnaire (EQ-5D-5L) is an instrument for measuring quality of life and to determine quality-adjusted life years in health economic studies. The first version of the instrument (with 3 instead of 5 response options) has been validated (Hurst et al. 1997) and was also found to be valid with CHD patients (Ellis et al. 2005) and depressive patients (König et al. 2005). The extended version of the instrument with 5 response options (EQ-5D-5L) was developed to reduce ceiling effects. Among other things, this version was tested and validated for use in Germany by Ludwig et al. (2017). The EQ-5D-5L covers five dimensions: Mobility, self-care, usual activities, pain/discomfort and anxiety/depression. Each dimension is scored on a 5-point scale. The answers to the individual items are combined into a quality-of-life index using established algorithms. In addition, the patients rate their health status on a visual analogue scale (EQ-VAS; value range 0-100). The EQ-5D-5L or EQ-VAS was found to be a valid instrument with acceptable sensitivity to change.

**Katz Index (ADL)**

The Katz index provides valid information about how (in)dependent a patient is in the in the performance of activities of daily living. The questionnaire published by Katz et al. (1963) is particularly suitable for older patients to clarify the need for care. The index consists of the following six items: bathing/washing, dressing, toileting, transfer, continence, and feeding (Katz et al. 1963). A maximum of 6 points can be scored, with 5-6 points for full functional ability, 3-4 points for moderate and ≤ 2 points for severe functional impairment (Wallace and Shelkey 2007).

**Montreal Cognitive Assessment Test (MoCa)**

The MoCa is a validated cognitive instrument that includes 30 questions and is suitable for the cognitive impairment and early stages of dementia (sensitivity: 90%; specificity: 87%) (Nasreddine et al. (sensitivity: 90%; specificity: 87%) (Nasreddine et al. 2005). The following aspects are tested: cognitive abilities, such as short-term memory, visual-spatial abilities, attention, concentration, working memory, language and orientation to time and place. A maximum of 30 points can be achieved. A sum score of ≤ 26 points indicates cognitive impairment (Nasreddine et al. 2005; Luis et al. 2009; Smith et al. 2007).

**Heart Quality of Life (HeartQoL)**

The HeartQoL is a 14-item self-report instrument for the assessment of health-related quality of life that has been shown to be reliable, valid and suitable for assessing change in different cardiac populations (Oldridge et al. 2014). In our study, we will use the German version. The internal consistency (Cronbach's alpha) was $\alpha = 0.92$ for the global HeartQoL scale, $\alpha = 0.91$ for the physical subscale $\alpha = 0.91$ and for the emotional subscale $\alpha = 0.87$ (Smedt et al. 2016).

**Hospital Anxiety and Depression Scale (HADS)**

The Hospital Anxiety and Depression Scale (HADS) assesses the severity of anxiety and depression in physically ill patients. It is a self-assessment instrument on a four-point Likert scale with 14 items, with higher scores indicating increased severity. The German version was validated in several large samples of cardiac patients, mainly with coronary heart disease (Herrmann 1997). In a review of 747 studies using the HADS for different purposes, a mean Cronbach's alpha of 0.82 for the anxiety subscale and 0.83 for the depression subscale was described (Bjelland et al. 2002; Petermann, 2011).

**Hand strength**

The hand strength is measured with the Martin vigorimeter (Gebrüder Martin, Tuttlingen, Germany). It is a pseudo-dynamic dynamometer that measures the pressure when subjects squeeze a rubber ball that is connected to a manometer through a tube. The pressure is measured in kilopascals (Desrosiers et al. 1995). The vigorimeter showed good test-retest reliability in studies (Sipers et al. 2016). The measurement device is suitable for use with geriatric patients, as the requirements on the abilities of the test person are minimal (Sipers et al. 2016). The measurement is carried out according to the Clinical Assessment Recommendations of the American Society of Hand Therapists (ASHT). After a brief introduction to the device with the patient sitting upright and the upper arm resting against the side of the torso in neutral rotation is measured. The elbow is held in 90° flexion, the forearm in neutral rotation and the wrist has a slight extension of 0 to 30°. The vigorimeter is kept free, the hand and forearm are not supported. To minimize fatigue effects, recovery breaks are observed between measurements.

**Short Physical Performance Battery (SPPB)**

The SPPB is a test battery for the measurement of motor function of the lower limb (Guralnik et al. 1994). It is a reliable and valid measurement instrument that is used primarily with geriatric patients to assess their mobility (Büsching 2015). The test battery consists of a balance test, 4-metre walk and the sit-to-stand test. A maximum of 12 points can be achieved, which provide information about the patient's impairment of daily living (0-3 points: severe impairment; 4-6 points: moderate impairment; 7-9 points: mild impairment; 10-12 points: no everyday life impairment) (Büsching 2015).

**6 Minute Walk Test (6MWT)**

The 6MWT is an easy-to-perform test that does not require any additional equipment or preparatory training of the test persons. It is used above all to show comparisons interventions, to assess functional status, or to estimate the risk of morbidity and mortality (Fiorina et al. 2007). There is sufficient evidence that the 6MWT is reliable and valid (Du et al. 2009). The test is performed according to the recommendations of the American Thoracic Society (ATS Committee 2002). Functional capacity is also an important prognostic factor in patients with heart disease to prevent future cardiac events and avoid mortality (Sawatzky et al. 2014). A study by Beatty et al (Beatty et al. 2012) shows that in patients with stable CHD, a 6-minute walk distance of less than 419m is associated with a twofold increased risk of suffering a cardiac event, in contrast to patients who walked over 481 m. Perera et al. (2006) confirm for older people a minimal clinically important difference of 20m and a substantial one of 50m (Perera et al. 2006; Enright and Sherrill 1998).

**Body composition (bioelectrical impedance analysis, BIA).**

The BIA is used to determine muscle mass. With the help of an alternating current, the Body resistance is determined. Taking into account the conductivity of the different body compartments, the body composition can be calculated (Kyle et al. 2003). A measurement usually takes only a few seconds and is completely painless and free of side effects (Reiss et al. 2016). The BIA is used in this context to determine the muscle mass in the context of the new European guidelines for the identification of sarcopenia (Cruz-Jentoft et al. 2019).

**Mini Nutritional Assessment (MNA)**

The MNA is a simple and well-validated questionnaire for the early detection of malnutrition in the clinical setting and is mainly used for geriatric patients (Rasmussen et al. 2010; Guigoz et al. 2002). The European Society for Clinical Nutrition and Metabolism explicitly recommends the use of this questionnaire, since it contains additional aspects to better assess the risk of malnutrition in elderly patients (Kondrup et al. 2003). In the present study, the validated short form of the MNA, the MNA-SF (Short Form) is applied (Kaiser et al. 2009). Rubenstein et al. (2001) prove a strong correlation between the MNA-SF and the MNA (r = 0.945) (Rubenstein et al. 2001). The questionnaire includes 6 items on the topics food intake and weight loss in the last three months, mobility, acute illness and psychological stress, neuropsychological problems and body weight. Per item up to three points can be scored, depending on the answer, the higher the score, the lower the risk of malnutrition. A total of 14 points can be reached where 12-14 points indicate a normal nutritional status, 8-11 points indicate an existing risk of malnutrition and 0-7 points stand for a present malnutrition.

**Pittsburgh Sleep Quality Index (PSQI)**

The PSQI is a validated and reliable questionnaire for self-assessment of sleep quality in a clinical setting (Doi et al. 2000; Buysse et al. 1989; Gentili et al. 1995). In addition to the self-assessment questions, it also contains 5 external assessment questions, whereby these are not included in the quantitative analysis. The 10 items of the self-assessment questionnaire refer to the retrospective subjective sleep quality of the past four weeks. The questionnaire consists of the components subjective sleep quality, sleep latency, sleep duration, sleep efficiency, sleep disturbances, use of sleep medication and daytime sleepiness. In addition to recording metric data such as sleep duration, the test subjects answer questions about sleep quality on a four-point scale. A total of 21 points can be achieved, whereby a total score of > 5 indicates impaired sleep quality (Buysse et al., 1989). For the total score, studies indicate a sensitivity of more than 80% and a specificity between 83-87% (Gentili et al. 1995; Buysse et al. 1989).

**Self-reported subjective memory impairment (SMI)**

Studies show that a subjective deterioration in memory performance is associated with the later development of dementia (Geerlings et al. 1999; Jessen et al. 2010). A longitudinal study with 2,415 participants without objectifiable cognitive deficits at baseline showed a hazard ratio (HR) of 1.83 [confidence interval (CI) 1.12-2.99] for the occurrence of any type of dementia

and 3.04 [1.36-6.81] for the occurrence of Alzheimer's dementia over three years. For subjective memory deterioration with apprehension, the HR was 3.53 [2.07-6.03] for the occurrence of any type of dementia and 6.54 [2.82-15.20] for the occurrence of Alzheimer's dementia (Jessen et al. 2010). Both criteria are assessed using the Self-reported Subjective Memory Impairment (SMI). The test person is asked the simple question "Do you have the feeling that your memory is getting worse?" If the answer is "yes", the second question is "Does this worry you?".

**Clinical Frailty Scale (CFS)**

The CSHA Frailty Index as well as the Clinical Frailty Scale (CFS) resulted from the the Canadian Study of Health and Aging (CSHA). This longitudinal study examined the health status of approximately 10,000 Canadians aged 65 years or older over a period of ten years (Rockwood et al. 2001). The aim was to develop a better understanding of frailty. The CSHA Frailty Index consists of 70 variables that describe different symptoms that are associated with frailty. The addition of the existing variables results in the index. The CFS comprises 7 levels and is used for clinical assessment of the functionality/fragility of a patient by the practitioner. Physical fitness, symptoms of illness, activities of daily living and cognition are included in the assessment. A CFS of 1 defines a very fit person and a CFS of 7 one who is completely dependent on outside help. Rockwood et al. (2005) describe a high correlation ($r = 0.80$) of CFS and the CSHA Frailty Index. Both assessments are valid and clinically useful constructs to determine frailty and make valuable frailty and thus provide valuable perioperative predictors (Rockwood et al. 2005). The German translation of the clinical frailty scale (CFS) consists of 9 categories, whereby a CFS of 1 defines a very fit person and a CFS of 9 a terminally ill person. From category 5 onwards, frailty is assumed (Singler, Gosch und Antwerpen, 2020). The CFS has a high reliability with an ICC (Intraclass Correlation Coefficient) of 0.97 ($p < 0.001$) and good construct validity with a Pearson correlation coefficient of 0.80 ($p < 0.01$) in comparison with the CSHA Frailty Index (Rockwood et al. 2005). The prediction of mortality is 87%, the association with functionality 91% and with mobility 90 % (Church et al. 2020; German Geriatric Society 2020).

**Informant Questionnaire on Cognitive Decline in the Elderly (IQCODE)**

The IQCODE is used to assess the cognitive state of a patient by a caregiver reference person (Jorm et al. 1989). It is a well validated and reliable (Cronbach's-α coefficient = 0.93) screening instrument and reliable (Cronbach's-α-coefficient $\alpha = 0.93$) screening instrument for the detection of cognitive impairment (Jorm et al. 1989; Jorm 2004). The German short version

consists of 7 items. The caregiver is asked to rate the cognitive impairment compared to the last two years on a Likert scale from "much better" (1 point) to "much worse" (5 points). At the end, an IQCODE sum and an IQCODE score. The IQCODE score is calculated from the sum of all the points divided by the number of questions answered. From a cumulative score of 23 or an IQCODE score of 3.29, cognitive impairment is suspected (Ehrensperger et al. 2010).

**Zarit Burden Interview (G-ZBI, German version)**

The Zarit Burden Interview (ZBI) is used to determine the subjective burden of caring of family caregivers (Zarit et al. 1980). The ZBI exists in different languages and has consistently shown good psychometric quality criteria in validation studies (Arai et al. 1997; Chan et al. 2005; Chattat et al. 2011). The interview consists of 22 items and covers the areas of "own health", "well-being", "finances", "social participation" and "relationship". The items are recorded on a five-point scale. Cut-off values according to Zarit and Zarit (1987) are: < 20 points (no stress), 21-40 points (low stress), 41-60 points (moderate stress) and > 60 points (high stress) (Zarit and Zarit 1987). Braun et al. (2010) demonstrate the validity with a high psychometric quality (Cronbach's-α coefficient α = 0.91) of the German version of the ZBI (Braun et al. 2010).

**SWE - General Self-Efficacy Expectancy Scale (short version)**

This questionnaire is developed based on Bandura's social-cognitive learning theory (Bandura 1979) and its concept of positive situation-action expectancy. The questionnaire presented here is a short version with 10 items for the general self-efficacy expectation. In the full version it measures the school-specific self-efficacy, study-specific self-efficacy and general self-efficacy. The test has been tested for reliability (reliability: Cronbach's alpha .71 to .89) and confirmed (given internal consistency) and is a recognized valid method. There are no subscales in the short version. The self-report questions refer to the perceived effectiveness of one's own ability to find solutions or to deal with difficulties and problems (Jerusalem and Schwarzer 2003).

**Hamburg Questionnaire on Hospital Stay (modified)**

This questionnaire is a shortened version of the original questionnaire, consisting of 62 items. The reduction is based on the practical decision not to confront patients with questions that have already been asked elsewhere. What is meant here are the first two scales in the questionnaire ("Information about yourself", A.1. to A.5. and "Information about your stay", B.1. to B.4.). The original questionnaire was developed to assess the hospital stay from the patient's

perspective (via five-point scale "yes" over "partly" to "no", as well as school grades 1-5). In addition to the collection of demographic data (not required in PRECOVERY), questions are asked about admission, nursing and medical care, ward rounds, information and education, assessment of the ward climate, accommodation and catering, ward organization, discharge and the success of the treatment (are collected in in PRECOVERY). The questionnaire ends with a request for an overall assessment and the possibility of free response to make critical comments. The questionnaire was pretested with N = 739 patients and proved to be reliable in differentiating between different wards and can be used in deficit analysis for quality management and certification purposes (Lecher et al. 2002).

**Life Orientation Test Revised (LOT-R)**

The LOT - R questionnaire consists of 10 items with a five point scale for patients. It measures the construct optimism. In this respect, the LOT-R is the most frequently used measuring instrument. The test has two subscales, optimism and pessimism, and was tested in the German translation on 4938 persons for quality criteria and norm values. The reliability of .59 (Cronbach's alpha) is to be regarded as unsatisfactory. The recommendation is that the questionnaire can be used for research purposes. The use in PRECOVERY follows the recommendation for scientific use of the questionnaire and is complemented by multiple other data collections as described (Glaesmer et al. 2008).

**Treatment Expectation Questionnaire (TEX-Q)**

The TEX-Q questionnaire, in the short version consisting of 15 items, aims to assess the expectations of patients regarding upcoming heart surgery using a ten-point scale. The content addresses the relief of symptoms, benefits of treatment symptoms, the benefits of treatment, expectations of health improvement, improvements in daily activities, improvement in quality of life, or the extent of expected risks. The questionnaire has been qualitatively tested and has a theory-based multidimensional structure regarding the recording of treatment expectations. The items used are considered (after examination) to be understandable and were assessed as fitting the theoretical framework (Alberts et al. 2020).

**Illness Perception Questionnaire (IPQ-B)**

The Illness Perception Questionnaire consists of 9 items in the short version. It showed good test-retest good test-retest reliability and high validity in relevant measurements. In this way, the short version of the IPQ enables a rapid assessment of patients' perception of their illness

(Broadbent et al. 2006). The long version of the Illness Perception Questionnaire consists of 14 dichotomous items, 18 tick-box items on a five-point scale and one free-response question. Thematically, the IPQ-R (German version) captures the illness assumptions of patients. The second part is the self-assessment of the disease with 18 scaled items. The correlation coefficient could be proven and shows a positive, linear correlation (Pearson correlations 0.32-0.63) (Broadbent et al. 2006). By differentiating between complaints/symptoms, the IPQ-R supports a differentiated view: depending on the diagnosis, patients develop specific beliefs about them, from which in turn coping behavior can be derived (Leventhal, H., Nerence, D.R. and Steele).

**Maastricht questionnaire - vital exhaustion questionnaire (short version)**

The nine-item short version of the Maastricht questionnaire allows a quick assessment of the degree of exhaustion level and proves to be valid (Balog and Konkolÿ Thege 2019 and Schnorpfeil et al. 2002). In the original version, the questionnaire captures the construct of vital exhaustion in 21 items (e.g. feelings of discouragement, lack of energy, increase in irritability). The German version was developed by the working group of the Department of Psychosomatic Medicine at the University of Lübeck under the direction of Priv.-Doz. Dr. med. Günter Jantschek in 2009. The items are to be answered on a three-point scale (no/?/yes) and determine a score from 0 to 42 points in the result. The internal consistency is very high with a Cronbach's alpha of .89 is very high (Billmann 2009).

**UCLA Loneliness Scale (short version)**

In PRECOVERY, the short version of the questionnaire with eight items is used, which is acceptable in terms of reliability and factorial reliability and factorial validity (Wilson et al. 1992). The questionnaire identifies three dimensions of loneliness: "feelings of loneliness", "perceived emotional isolation" and "perceived social isolation" and "perceived social isolation". In the full version it consists of 20 items. It is scaled fivefold and, with a Cronbach's alpha of 89, is highly internally consistent (Döring and Bortz 1993).

**FIMA-Questionnaire**

The utilization of services is assessed with the help of a modified version of the questionnaire for the assessment of health services in elderly patients (FIMA). The FIMA is a standardized and generic questionnaire for the assessment of the use of health-related resources (Seidl et al. 2015) and enables the recording of health care services in the sectors of outpatient medical

care, therapeutic medical care, remedies, nursing and domestic care, medication, rehabilitation measures, inpatient and outpatient care, rehabilitation measures, inpatient and day-care hospital stays as well as housing the calculation of the corresponding individual costs for health care services. The validity of the self-reports between the quality of life and the resources used in the area of nursing and domestic care ranged was between 0.52 and 0.58 (Phi coefficient).

**Process evaluation**

The mixed-methods process evaluation is based on the MRC Guidance for Process Evaluation for Complex Interventions (Moore et al. 2015) and the Normalization Process Theory (May et al. 2007; May et al. 2009). On the one hand, the quantitative process evaluation records the concrete introduction and implementation (e.g., program fidelity, dose, adaptations, reach, and framework conditions) of the originally planned intervention as well as possible effects on the impact of the intervention. The qualitative process evaluation captures the perspective of patients in the intervention group as well as of other participants in the study and the "Karl-Heinz intervention" and explores factors that hinder and promote the implementation of the intervention. In addition, it explores the expectations of patients in the intervention and control groups, the experience of the cardiac intervention itself and the time after the intervention.

**Targets (quantitative)** (Moore et al. 2015):

- **Program fidelity**
- **Dose**
- **Adaptations**
- **Outreach**
- **Mechanisms of intervention implementation, participant responses, and adaptations**
- **Influence of basic conditions**

**Program fidelity** is measured by checking whether Karl-Heinz is offered in the facilities as originally planned, i.e. whether all treatments are available.

**Dose** measures the actual implementation, i.e. whether all treatments were then offered.

**Adaptations** mean changes in the implementation, e.g. with regard to the content or duration of the trreatments.

**Outreach** is mapped by recording the use of the treatments by the patients (participation rate).

**Mechanisms of the impact of the intervention:** The response of patients to the intervention and the patients' response to the intervention and the implementers' assessment of the actual implementation (unexpected pathways and consequences). In addition, views on the implementation of Karl-Heinz as well as the subjectively perceived implementation quality of Karl-Heinz will be recorded (patients, relatives, facility managers, physicians in the recruitment centers, multipliers and staff in the facilities as well as staff in the study center).

**Framework factors:** Here, factors are recorded that influence the intervention or are influenced by it. Also, mechanisms whose aim is to maintain the original state can act on the intervention from the outside.

Framework factors occur at different levels (micro, meso and macro) and in different contexts (health system, facility, staff, patient) (Threapleton et al. 2017). It can be e.g. laws, resources, organizational forms, qualifications, experience and many other aspects that influence the intervention from the outside. Framework factors are defined at the levels of management, multipliers, staff in the facilities and, of course, patients and their relatives. Also perceived facilitating and inhibiting factors are also asked about.

**Targets (qualitative)**

**In patients (intervention group and control group)**

• View of the upcoming cardiac procedure

• View of the time after cardiac procedure

• Wishes for the course of health

• Lifestyle changes (e.g., diet, exercise, smoking)

**Additionally in patients in the intervention group**

• Acceptance, implementation and evaluation of prehabilitation

• View of the implementation of prehabilitation as well as reactions and adaptations on the part of the participants

• Effects of prehabilitation on the experience of the procedure

• Satisfaction with prehabilitation as well as perceived benefits

• Identification of barriers and conducive factors in implementation (May et al. 2007).

**For close relatives (intervention group and control group)**

• View of the upcoming cardiac procedure own perspective

• View of the time after cardiac procedure, own perspective

• Wishes for the course of health

**In addition, in the case of related persons of the intervention group**

• Acceptance, implementation and evaluation of prehabilitation, own view

• Effects of prehabilitation on one's own well-being as well as for the patient

• Perception of the conversation with relatives during prehabilitation

• Satisfaction with prehabilitation

• Identification of barriers and conducive factors in prehabilitation (May et al. 2007)

**In the case of multipliers**

• View of the implementation of prehabilitation in the facility

• View of one's own role

• View of the patients receiving the prehabilitation

• Assessment of the usefulness of prehabilitation, evaluation

• Suggestions for improvements in prehabilitation? Wishes? Advice to the study team

**For employees**

• View of the implementation of prehabilitation in the facility

• View of one's own role

• View of the patients receiving the prehabilitation

• Assessment of the usefulness of prehabilitation, evaluation

• Suggestions for improvements in prehabilitation? Wishes? Advice to the study team

**In the case of doctors in recruitment centers**

• View of one's own role

• View of ambulance conversations

• View of interventions

• Perception of the patient's health history

• Assessment of the usefulness of prehabilitation, evaluation

• Suggestions for improvements in prehabilitation? Wishes? Advice to the study team

**For general practitioners of patients in the IG**

• View of the implementation of prehabilitation in the facility

• View of the patients receiving the prehabilitation

• Assessment of the usefulness of prehabilitation, evaluation

• Suggestions for improvements in prehabilitation? Wishes? Advice to the study team

# 6 Study Design and Participating Centers

**Study design**

PRECOVERY is a randomized, controlled, multicenter, two-arm, assessor-blinded study that evaluates the effectiveness of two-week inpatient or semi-inpatient prehabilitation prior to elective cardiac procedure as measured by EQ-5D-5L and mortality one year after cardiac procedure compared with the SMC. The patients in the SMC are cared for according to the usual standard in the phase before the planned operation.

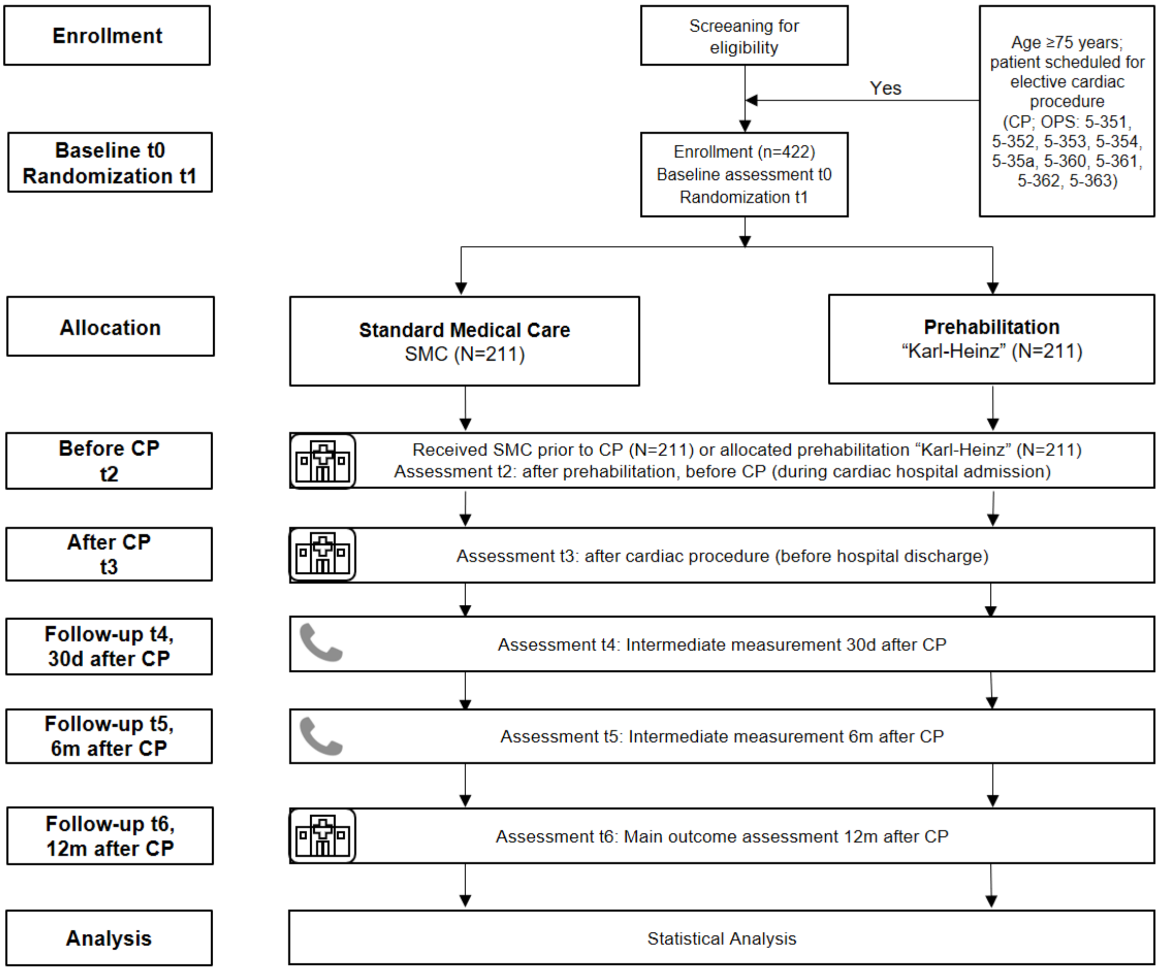


**Figure 2**. CONSORT diagram of the PRECOVERY randomized controlled trial.

In the recruitment centers, patients who are scheduled to undergo surgery in 4-6 weeks are screened according to the inclusion and exclusion criteria. Eligible patients will be recruited (t0) and randomized (t1).

In the study, a total of six assessment time points are planned for the patients. For the quantitative process evaluation, a further survey will only be carried out on patients in the intervention group after prehabilitation (between t0/t1 and t2):

| | |
|---|---|
| t0 /t1 | Baseline |
| post | Assessment after prehabilitation (intervention group only) |
| t2 | Assessment before cardiac procedure (CP), after prehabilitation |
| t3 | Assessment after CP, before hospital discharge |
| t4 | Assessment 30 days after CP |
| t5 | Assessment 6 month after CP |
| t6 | Final assessment 12 month after CP |

The collected data are entered into the eCRF of the database (secuTrial®) by the study physicians and study nurses. Assessments at t4 and t5 are conducted via phone.

In addition, the study will collect data to assess the health economic benefits of the intervention and evaluate the implementation process of the intervention.

**Participating Centers**

Recruiting centers:

- Department of Cardiovascular and Thoracic Surgery, University of Goettingen Medical Center, Goettingen, Germany
- Department of Cardiology and Pneumology, University of Goettingen Medical Center, Goettingen, Germany
- Department of Cardiothoracic, Transplantation and Vascular Surgery, Hannover Medical School, Hannover, Germany
- Department for Thoracic, Cardiac and Vascular Surgery, Ulm University Medical Center, Ulm, Germany
- Department of Cardiac, Thoracic and Vascular Surgery, Braunschweig Municipal Hospital, Braunschweig, Germany
- Department of Cardiac Surgery, Oldenburg Hospital, Oldenburg, Germany
- Immanuel Clinic Bernau, Brandenburg Heart Center, Bernau, Germany

Recruiting and Prehabilitation centers:

- Schüchtermann-Schiller'sche Clinic, Bad Rothenfelde, Germany
- Heart and Vascular Center Bad Bevensen, Bad Bevensen, Germany

Prehabilitation centers:

- Rehabilitation Center Oldenburg, Oldenburg, Germany
- Clinic and Rehabilitation Center Lippoldsberg, Wesertal, Germany

- Kirchberg Clinic, Bad Lauterberg, Bad Lauterberg, Germany
- ZAR Center for Outpatient Rehabilitation GmbH, Ulm, Germany
- Clinic Fallingbostel, Bad Fallingbostel, Germany
- Brandenburg Clinic, Bernau Waldsiedlung, Germany

# 7 Study population

**Inclusion and exclusion criteria**

**Inclusion Criteria:**

1. Patients aged ≥ 75 years who are scheduled to undergo one of the following elective cardiac procedures (see Appendix for further details):

    a) Replacement of heart valves with prostheses (5-351),

    b) Replacement of heart valve prostheses (5-352),

    c) Valvuloplasty (5-353),

    d) Other operations on heart valves (5-354),

    e) Minimally invasive surgery on heart valves (including catheter-based aortic clap implantation – TAVI and transvenous clip reconstruction of the mitral valve – MitraClip) (5-35a),

    f) Deobliteration (endarterectomy) of the coronary arteries (5-360),

    g) Application of an aortocoronary bypass (5-361),

    h) Application of an aortocoronary bypass by minimally invasive technique (5-362) or

    i) Other revascularization of the heart (5-363)

2. Sufficient independence and capacity for self-sufficiency and participation in prehabilitation
3. Insurance status of all patients from Lower Saxony with the AOK
4. Ability to give a consent
5. Willingness to voluntarily participate in the study after being informed with a signed declaration of consent
6. Sufficient knowledge of the German language to complete/answer the questionnaires

**Exclusion criteria:**

1. Lack of capacity to consent
2. Katz Index 0
3. Need for treatment in acute care hospitals
4. Severe dementia; severe mental disorders (acute psychosis, major depressive episode, acute suicidal tendencies), acute delirium
5. Diagnosis of acute alcohol or drug abuse

6. Unstable angina pectoris
7. Heart Failure, NYHA IV
8. Myocarditis, HOCM, main stem stenosis ≥ 80%
9. Severe therapy-refractory cardiac arrhythmias
10. Status after aortic dissection
11. PAVK Stadium ≥ III after Fontaine
12. Symptomatic carotid stenosis or carotid stenosis requiring treatment
13. Renal insufficiency requiring dialysis
14. Hepatic insufficiency, Child B and Child C
15. Advanced (metastatic) oncological disease
16. Neurological, orthopedic or rheumatic comorbidities that speak against physical training
17. Participation in another intervention study (participation in registry studies is permitted)

As part of this study, relatives or close persons are also interviewed. Relatives or close relatives are defined as persons aged ≥18 years who provide social support to the patients and are a direct contact person.

**Process evaluation: participant inclusion and exclusion criteria**

In addition to patients and people close to them (same inclusion and exclusion criteria of the main study), the following groups of people are included in the process evaluation:

- Managers of the participating rehabilitation facilities (no inclusion or exclusion criteria)
- Multipliers and their representatives in the rehabilitation facilities (no inclusion or exclusion criteria)
- Employees of the rehabilitation facilities that carry out Karl-Heinz units directly (therapists, counsellors, clinical social workers, etc.) or indirectly (physicians, nurses).

Inclusion Criteria:

1. Care of patients undergoing prehabilitation
2. Participation in a training course by the multiplier
3. Activity in the institution (in perspective) during the entire term

- Cardiac surgeons at the recruitment centers (no inclusion and exclusion criteria)
- General practitioners of patients in the intervention group (inclusion criterion: medical care of patients during the 12-month follow-up)
- Research assistants who provide training for the multipliers and their representatives or supervision during the study (no inclusion or exclusion criteria).

**Number of study participants**

A total of 422 patients (211 intervention group, 211 SMC) will be enrolled in the PRECOVERY study.

**Process evaluation: number of study participants**

<u>Quantitative Process Evaluation:</u>

• Patients: n=422 subjects parallel to the main study
• Related parties: max. 422 people (realistically much fewer)
• Heads of rehabilitation facilities: n=7
• Multipliers: n=7, trainings: n=7 plus representatives: n=7
• Employees in rehabilitation facilities: n=35 (5 per facility)
• Study team staff: n=2 (training)

<u>Qualitative process evaluation</u>

• Patients: intervention group: n=16, control group: n=8
• Related parties: intervention group: n=16, control group: n=8
• Multipliers: n=7, (one focus group)
• Employees in the rehabilitation facilities: n=16 (2 focus groups)
• General practitioners: n=16 (2 focus groups)
• Study physicians: n=9

**Recruitment**

The PRECOVERY recruitment will be integrated into the standard procedures of the participating heart centers. In these recruitment centers, patients who are scheduled to undergo a cardiac procedure in 4-6 weeks are screened, informed, recruited for the study (t0) and randomized (t1) according to the inclusion and exclusion criteria during a preoperative, outpatient appointment in cardiology or cardiac surgery departments. In order to support the recruitment process, all referring centers will be informed about the study in advance and additional flyers will be created to increase patient interest.

If the recruitment figures fall short of expectations, the evaluation concept is designed through the use of the FIMA questionnaire in such a way that an extension to the inclusion of insured persons from other health insurance companies on the legal basis §630a BGB is also conceivable in Lower Saxony. Due to the goal of highest-quality routine data analysis for as many participants as possible, this would only be seen as a fallback strategy.

**Process Evaluation: Recruitment**

**Patients and relatives**

For quantitative process evaluation, no recruitment of patients and close associates is required, as the few questions are included in the eCRF of the main study. For the qualitative process evaluation, patients and close persons are recruited as part of the approach to the main study (PEQUAL 1a V1, PEQUAL 1b V1). The aim is to recruit 16 patients and close relatives from the future intervention group. In addition, a total of 8 patients and 8 close persons from the control group will be recruited.

**Facility management, multipliers, employees**

For the quantitative process evaluation, the facility managers as well as the multipliers/representatives will be contacted by the study team of the process evaluation (team PE) (PEQUAN 1c V1.1), or via phone (facility management). The employees will be approached by the multipliers in the facilities and will be invited to participate anonymously.

As part of the training of multipliers/representatives and employees in the rehabilitation facilities, the participants are invited to voluntarily participate in the evaluation (PEQUAN 5c.1 V1.1, PEQUAN 5d V1). The training study staff/multipliers are asked to fill out a short protocol as part of their work (PEQUAN 5 V1, PEQUAN 5c.2 V1). Personnel changes are noted by the multiplier/representative (PEQUAN 5c.3 V1).

**Study physicians and general practitioners** (annexes in the amendment)

The clinicians who see the patients in the outpatient clinic and/or perform the operation are written to by the PE team, contacted via phone and invited to participate. The names of the primary care physicians of the participating patients will be recorded when they are included in the study. At the end of the study, the general practitioners will be contacted in writing and by telephone by the PE study team and invited to participate in the study.

# 8 Curriculum

**Procedure for clarifying and obtaining consent**

All patients who meet the inclusion and exclusion criteria will be approached by the study physician or study nurse when they visit the cardiology/cardiac surgery department to see if they are interested in the study. If they are interested, patients will receive detailed patient information and the declaration of consent to the clinical trial. In addition, insured persons of the AOK Lower Saxony receive the declaration of participation in the contract for special care as well as an information sheet on data protection and insured persons of the AOK Baden-Württemberg the treatment contract of the prehabilitation center to read. Accompanying relatives or accompanying close persons will be given the information on relatives and the corresponding declaration of consent. This is followed by a detailed study information by the study physician and after sufficient time to think about it, the written consent of the patients and the relative will be collected. The consent documents also include the explicit consent of the participant to the disclosure of statutory health insurance routine data (consent to data processing) from the corresponding health insurance company (AOK Lower Saxony or AOK Baden-Württemberg) to the health economic evaluation partner (UKE Hamburg, Prof. König) for evaluation purposes.

No study-specific examinations will be carried out without the prior consent of the patient to participate in the study. In the informed consent form, patients can decide whether residual amounts of blood samples taken for the study may be retained and used for further scientific investigations in the field of cardiac surgery/cardiology/ geriatrics.

**Process evaluation**

<u>Quantitative Process Evaluation</u>

• Patients: The information on quantitative process evaluation can be found in the written study information (Annex IIa) of the main study. In addition, it is stated there that in the event of assignment to the intervention group, personal data (real name, ID, contact details) will be passed on to Team PE. Furthermore, the completion of the patient diary by employees and by the patients themselves during prehabilitation are listed (PEQUAN 5a V1.1). Furthermore, after the prehabilitation, they are called by a member of the PE team and asked about the prehabilitation stay in a standardized

way. The permission to send the therapy plan of the prehabilitation to the PE team as well as the release of the family doctor from confidentiality in a group discussion are also listed.

The information is provided by the study physician at the recruitment center as part of the main study.

- Related parties: You will receive information about the process evaluation as part of the information of the main study (Annex III) and will be informed by doctors at the recruitment center.
- Heads of the institutions (consortium partners): The heads of the institutions will be informed by telephone about the sending of the survey form on the institution.
- Multipliers: The multipliers receive a letter (PEQUAN 1c V1.1) and study information (PEQUAN 2c V1) by post and are informed orally by a study employee of the PE team before signing the informed consent (PEQUAN 3c V1.1).
- Employees in the institutions: Here, the study information is integrated into the questionnaire (PEQUAN 4d.1 V1.1, PEQUAN 4d.2 V1.1, PEQUAN 4d.3 V1.1), the survey is anonymous.

<u>Qualitative process evaluation</u>

- Patients and close persons: When approached by study assistants or doctors, an invitation to participate in the study will be handed over (PEQUAL 1a V1, PEQUAL 1b V1). If they are interested, patients/loved ones will receive written information (PEQUAL 2a V1.1, PEQUAL 2b V1.1) and information documents (PEQUAL 3a, 3b V1.1). The oral explanation is carried out by the study doctor. Participation in the interviews is voluntary and can be done in addition to the main study, participation in the main study is also possible without participation in the interviews.
- Multipliers: You will receive letter by post (PEQUAL 1c V1), written information material (PEQUAL 2c V1.1) with the documents for the video implementation of the focus group (PEQUAL 6c, 6d, 6f V1, PEQUAL 6c.1, 6d, 6f V1) and educational documents (PEQUAL 3c, 3d V1) on the focus groups and, if interested, will be informed orally by telephone by a scientific employee of the PE team, before informed consent is given.
- Employees in the facilities: If they are interested, they will receive cover letters (PEQUAL 1d V1.1), written information material (PEQUAL 2d V1.1) with the

documents for the video implementation of the focus group (PEQUAL 6c, 6d, 6f V1, PEQUAL 6c.1, 6d, 6f V1) and the educational documents (PEQUAL 3c, 3d V1) by the multiplier and will be informed orally by telephone by a scientific employee of the PE team.

- General practitioners: General practitioners are contacted by post by cover letter (PEQUAL 1f V1.1) and by telephone and asked about their interest in participating in a focus group. In the positive case, they will be sent study information (PEQUAL 2f V1.1) with the documents for the video implementation of the focus group (PEQUAL 6c, 6d, 6f V1, PEQUAL 6c.1, 6d, 6f V1) and educational documents (PEQUAL 3f V1.1), and oral information will also be provided by telephone by a scientist from the PE team. In addition, you will receive an invoice form for an expense allowance of 100 euros (PEQUAL 6f V1.1).
- Study physicians: You will be contacted by letter by post (PEQUAL 1e V1.1) and by telephone by study staff of the PE team. If you are interested in participating, you will receive the study information (PEQUAL 2e V1.1), documents for video implementation (PEQUAL 6c, 6d, 6f V1, PEQUAL 6c.1, 6d, 6f V1) and educational documents (PEQUAL 3e V1.1). An oral explanation is provided by telephone by a research assistant of the PE team.

As a general rule, the declaration of consent to participate in the study is dated and signed by the study participant as well as the study physician or a research assistant of the PE team. In the case of on-site information, the signatures are usually given at the same time, if the participant still wishes time to think about it, he or she takes two unsigned copies of the consent with him or her and signs and first, only then does the study doctor/study employee sign. In the case of clarifications by telephone, the first step is the signature of the informed person, who sends both copies signed by him to the PE team. Before the start of the course, she will then receive an additional copy signed by the scientist by post.
Only general practitioners receive an expense allowance of 100 euros for participation in the focus group. All other participants in rehabilitation facilities and recruiting facilities conduct the interviews and focus groups during their working hours (consortium partners). Patients and loved ones do not receive any expense allowances.

**Randomization**

After baseline assessment, randomization is performed in a 1:1 ratio stratified by study site, patient age (<81 years vs. ≥81 years) and gender (block randomization with random block lengths). Randomization will be performed by the study physicians of the recruiting centers. The individual study nurses of the recruiting centers remain blinded. Patients in the control group (n=211) are provided with standard medical treatment (cardiac surgery without prehabilitation). In the event of randomization to the intervention group (n=211), insured persons of AOK Niedersachsen will receive the declaration of participation in the new form of care of AOK Niedersachsen or insured persons of AOK Baden-Württemberg will receive the treatment contract of the participating pre-rehabilitation center, and inpatient or semi-inpatient admission to the cooperating prehabilitation center will be planned. After two weeks of prehabilitation, the elective procedure must be planned within 2-4 weeks in the recruitment centers in order to maintain the treatment effect of an intensified prehabilitation measure.

**Study intervention**

Patients in the intervention group (n=211) receive prehabilitation as a two-week preventive, intensive and full-day measure in cardiac rehabilitation facilities ("prehabilitation centres") for targeted, holistic preparation for the procedure. Prehabilitation includes a multimodal, interdisciplinary therapeutic approach that is individually adapted to the patient to achieve the best possible physical and mental health with improved functional reserve (resource-oriented, intrinsic capacity) before the planned procedure. For the period of prehabilitation, patients are admitted to one of the participating rehabilitation clinics (prehabilitation centres). At the Centre for Outpatient Rehabilitation (ZAR) Ulm, the measure takes place in a day-care setting. As an intervention, an interactive treatment concept consisting of various modules of sports and exercise therapy as well as occupational therapy, informational-educational, behavioral psychology and psychosocial measures will be implemented in the prehabilitation centers (details in the appendix "Treatment manual"). From a defined pool of therapy modules, the attending physician of the prehabilitation center puts together the respective treatment plan for each patient.

**Table 5**. Example of a weekly schedule of prehabilitation.

| | Monday | Tuesday | Wednesday | Thursday | Friday |
|---|---|---|---|---|---|
| 07:30 | Laboratory | Diagnostics | Laboratory | Diagnostics | Laboratory |
| 08:00 | Breakfast | | | | |
| 08:30 | | | | | |
| 09:00 | Exercise Therapy | Occupational therapy | | Occupational therapy | Social Counselling |
| 09:30 | | | | | |
| 10:00 | Breathing Therapy | | Breathing Therapy | | Breathing Therapy |
| 10:30 | Visit | Visit | Visit | Visit | Visit |
| 11:00 | | Exercise Therapy | Exercise Therapy | | |
| 11:30 | Cognitive Therapy | | | Cognitive Therapy | |
| 12:00 -13:30 | Lunch | | | | |
| 14:00 | | | | | |
| 14:30 | Massage | Cognitive Therapy | | Exercise Therapy | Exercise Therapy |
| 15:00 | | | Occupational therapy | | |
| 15:30 | | | | | |
| 16:00 | Health education | Conversations with relatives | Psychosocial intervention | Psychosocial intervention | Health education |
| 16:30 | | | | | |
| 17:00 | Breathing Therapy | Breathing Therapy | Breathing Therapy | Breathing Therapy | Breathing Therapy |
| 17:30 | Dinner | | | | |

The individual modules can be summarized as follows:

The following modules are integrated into the "Karl-Heinz" intervention at PRECOVERY:

- Module 1 "Sports and Exercise Therapy" (aerobic endurance training, dynamic strength training, flexibility and coordination training as well as breathing therapy)
- Module 2 "Occupational Therapy" (everyday training)
- Module 3 "Cognitive Training" (Cognitive Therapy)
- Module 4 "Psychological support" (psychosocial intervention, preparation for intervention, reduction of anxiety, relaxation techniques)
- Module 5 "Disease-specific training and nutritional counseling" (sleep hygiene, health education, lifestyle, health literacy, nutritional counseling)
- Module 6 "Conversations with relatives" (legal aspects, e.g. power of attorney, living will, advice for families/relatives)
- Module 7 "Nutritional Therapy and Miscellaneous" (e.g.: "special hygiene training")

In order to standardize the prehabilitation program, a team of experts from all participating centers and AOKs (2-day workshop in May 2021) designed a treatment manual ("Karl-Heinz") (elaborated version is available). Based on this, a team training concept was created, which

was standardized in the form of a training manual. Initially, there will be a short training of the multipliers on areas of responsibility as a "multiplier" in the prehabilitation center. Subsequently, a team of multipliers will receive virtual training on the individual modules using the intervention manual. The multipliers will then conduct on-site training sessions for the intervention staff of the respective prehabilitation centers ("train the trainer"). The individual elements and programs for prehabilitation are not new, but the embedding of a heart-specific program in a structured trans-sectoral concept represents a previously unaddressed need in the care of elderly patients.

**Recording of the target variables**

For a high level of study adherence (minimization of drop-outs/lost to follow-up), the study visits are coordinated with routine rounds as far as possible or the participants are reimbursed for travel expenses and parking fees and, if necessary, home visits are carried out for follow-up appointments. A "minimal data set" is defined, which can also be collected by telephone if necessary. A validated telephone questionnaire is available for the primary endpoint EQ-5D-5L. An external assessment of the patient's cognitive abilities by a close relative (IQCODE) and a survey on the subjective burden of the relative (G-ZBI) are carried out at the beginning of the study (t0), after six (t5) and twelve months (t6). A detailed list of the targets and data collection time points can be found in the overview table (see Table 1).

The health economic evaluation will be carried out by the Institute for Health Economics and Health Services Research, UKE (headed by Prof. H.-H. King). The following data will be collected for the health economic analysis:

(1) The personnel and material costs of prehabilitation are calculated according to the principles of business cost calculation using the performance documentation of the rehabilitation facilities.

(2) In order to determine the use and costs of health services in the follow-up period, the FIMA questionnaire ('Questionnaire for the collection of health services in old age') adapted to the project and standardized monetary assessment rates will be used. In addition, the above-mentioned service utilization and cost data will also be collected using routine health insurance data from the AOK Lower Saxony and AOK Baden-Württemberg. Since routine health insurance data is expected to be available for a maximum of 85% of the subjects, the analysis of this subsample will be carried out as a sensitivity analysis. The higher reliability of the routine health insurance data thus enables an additional classification of the primary data analysis. If, in an interim analysis after 25% recruitment, it is foreseeable that the collection of routine health insurance data will be available in 80% of PRECOVERY patients, the FIMA collection will be dispensed with in the further course. Not only does the routine health insurance data have a higher data quality, it seems plausible that the

completeness of the remaining primary data may be increased as a result of discontinuing of the FIMA. The participants will have fewer questions to answer and therefore, they will likely become less tired while participating in the data collection process. The health economic analyses will then carried out solely on the basis of higher-quality routine health insurance data. All costs are collected in the period of the last one to six months at the survey times t1, t2, (t4), t5 and t6 and compared between the intervention group (IG) and the control group (CG).

(3) Determination of utility values (quality-adjusted life years – QALYs): QALYs are calculated on the basis of the EQ-5D quality of life questionnaire at the survey times t1 to t6.

(4) Determination of cost-effectiveness: For the analysis of cost-effectiveness after 12 months, the differences in costs and QALYs between the IG and the CG are determined in the follow-up and related in the form of incremental cost-benefit ratios. The non-parametric bootstrap method will be used for uncertainty analysis. This allows the skewed distribution of cost data and the covariance of costs and QALYs to be taken into account. In addition, the method of net benefit regression and, based on this, the construction of cost-effectiveness acceptance curves are used to visualize the statistical uncertainty of the cost-benefit ratio. Figure 3 provides an overview of the duration of the study for the individual patient, including the data collection time points (t0 to t6). The duration of study per patient is 14 months.

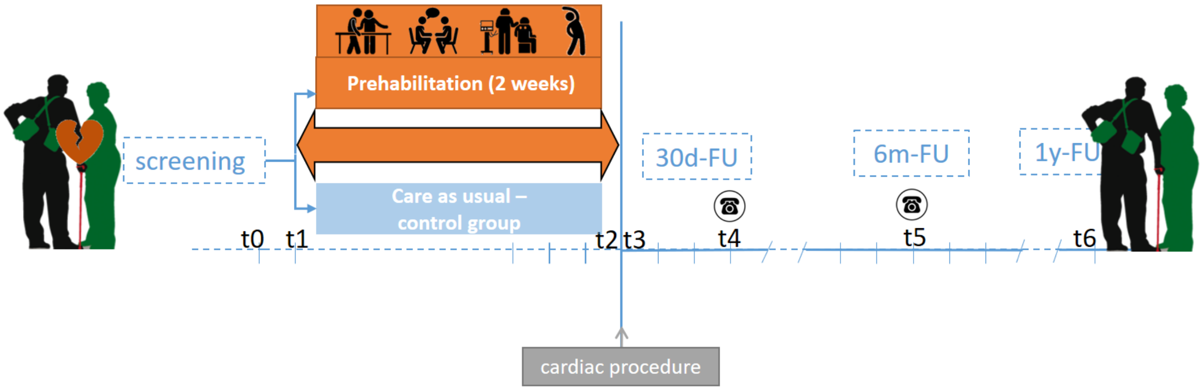


**Figure 3**. PRECOVERY study design.

## Process evaluation

Quantitative process evaluation: Targets and data collection

The data collection includes the following data sources (Table 1) in the intervention and control groups: standardized questionnaires for:

- All patients at inclusion (t0/t1), after surgery (t3), after 30 days (t4), after 6 months (t5) and after 12 months (t6) (PEQUAN 4a.1 V1). The data will be collected in a standardized interview by the study assistant at the <u>recruitment center</u>.

- All close persons at study begin (t0/t1) and after 6 and 12 months (t5/t6) after the cardiac procedure (PEQUAN 4b V1.1). The data will be collected in a standardized interview by the study assistant at the recruitment center.

Additionally in the intervention group:

- Standardized survey of patients shortly after prehabilitation (before hospital stay) (PEQUAN 4a.2 V1.1). The data will be collected in a standardized telephone interview by a member of the PE team. Here, patients who participate in the qualitative process evaluation will not interviewed in order to avoid increasing the number of surveys.
- Diary for Karl-Heinz intervention: During prehabilitation, practitioners and patients fill out the patient's diary (PEQUAN 5a V1.1), which consists of two parts, after each Karl-Heinz unit. The patients receive the diary from the multiplier at the beginning of their stay in the prehabilitation center and return it at the end (in a sealed envelope).
- There are two booklets: In Part A of the practitioners' booklet, the implementation, duration and, if necessary, adjustments to each unit will be recorded. In Part B, more detailed data on implementation, abnormalities in implementation, and a small amount of personal clinical data will be collected by the respective practitioner as part of a final documentation at the end of each module. In the patient's part of the booklet, patients assess each individual unit. They indicate whether the session was strenuous. In addition, once a day during their stay, they provide information on how they felt on that day. The diary is bound into two separate notebooks so that the practitioners cannot see the patient's entries. The two-part diary is sent by the multiplier to the PE team.
- The therapy plan is also used as a data source. It is sent by the multiplier to the PE team. The practitioners' real names are blacked out or removed.

Managers of the prehabilitation centers, multipliers and employees of the facility fill out questionnaires themselves before admitting the first patient (PEQUAN 4c.1 V1, PEQUAN 4d.1 V1.1, PEQUAN 4g.1 V1.1) and after discharge of the last patient from the facility (PEQUAN 4c.3 V1, PEQUAN 4d.3 V1.1, PEQUAN 4g.2 V1.1). The multipliers and employees of the institutions also fill out a questionnaire after 6 months (PEQUAN 4c.2 V1, PEQUAN 4d.2 V1.1). The questionnaire for the facility management refers to data about the facility. The surveys of multipliers and employees include questions on the implementation of prehabilitation and framework conditions. In addition, a document (PEQUAN 5c.3 V1) records changes and training data of multipliers and employees in the rehabilitation facilities by the respective multiplier. The employees in the facilities receive the questionnaires from the multipliers. The forms are handed over with an envelope and must be filled out anonymously. The participants

first pass on the sealed envelope with the completed questionnaire to the multiplier, who then sends it to the PE study team.

During the multiplier training courses at the beginning of the study, multipliers and their representatives fill out evaluation forms (PEQUAN 5c.1 V1.1) themselves, and the schoolers keep a short protocol (PEQUAN 5 V1). Later, employees of the institutions that are trained to deliver the Karl-Heinz intervention fill out an evaluation themselves (PEQUAN 5d V1) after the training by the multiplier. The multiplier briefly records the course (PEQUAN 5c.2 V1). Follow-up training, e.g. following a change in multiplier or employees, is evaluated in the same way as the training of employees. The questionnaires are handed out by the trainers at the end of the training. The collection of the anonymously completed forms takes place in a box with a slot, which is kept closed and later sent to the PE team.

**Qualitative Process Evaluation: Contents and Data Collection**

The data for the qualitative process evaluation will be collected in interviews and focus groups with study participants. The interviews and focus groups are conducted by research assistants, who are members of the PE team. They will be trained in detail on the techniques in advance. The interviews follow guidelines that were prepared in advance after Helfferich (Helfferich 2022). There are currently no guidelines in the appendix, but the topics for the respective surveys. The interviews are recorded with two recording devices (for safety's sake). In addition, the interviewer takes field notes. At the end of the interview, an appointment may be made for a follow-up interview.

**Interviews**

Patients and related persons: Patients and close persons will be contacted by telephone (PEQUAL 6a, 6b V1) to make an appointment for the 1st interview. The scientific employee of the PE team will answer any questions that may arise. The scientific employee of the PE team will conduct guideline-based, semi-structured individual interviews with patients/persons close to them by telephone on the agreed date. Interviews with patients from the intervention and control groups are conducted after inclusion (but before prerehabilitation in patients in the intervention group, at best on day 2-5 after inclusion) by telephone (t0/t1), as well as 30 days (t4) and 12 months (t6) after the procedure. In addition, patients in the intervention group will be interviewed after prehabilitation (before the procedure) (all guidelines: PEQUAL 4a V1). Interviews with close persons of both groups will be conducted after t4 (30 days to 60 days after the procedure) and after 12 months (PEQUAL 4b V1). The interviews differ in their desired length (30 to 40 minutes). Study physicians: The approx. 30-minute interviews with the treating/operating hospital physicians take place when the last patient with surgery in the

respective clinic has completed the quantitative follow-up survey after 12 months (t6) (PEQUAL 4e V1.1). During the interviews, socio-demographic data will be collected from the participants (PEQUAL 5a V1, PEQUAL 5b V1, PEQUAL 5e V1.1).

**Focus Groups**

The 90-minute focus groups of multipliers (PEQUAL 4c V1), staff (PEQUAL 4d V1) and general practitioners (PEQUAL 4f V1.1) will be led by two facilitators on the basis of guidelines. The focus groups will be conducted via video conference. The focus groups will be recorded on two recording devices. After consent to the study, the participants will be sent a short socio-demographic questionnaire in advance, which will be filled out and returned to the PE team (PEQUAL 5c V1.1, PEQUAL 5d V1.1, PEQUAL 5f V1.1).

**Process Evaluation: Data Entry**

Quantitative Process Evaluation: The data entry of the patient surveys and the interviews of related persons will be carried out by the study assistant at the recruitment center together with the data of the main study. The data of the standardized interview of the patients from the intervention group immediately after prehabititation will be entered by members of the PE team. The paper-based patient diary and therapy plan will also be entered by a study employee from the PE team.

All paper-based questionnaires of the surveys of facility management, multipliers, employees of the institutions and employees of the UMG study team will be entered into the database by study staff of the PE team. All data entries of paper-based questionnaires are carried out according to the four-eyes principle: After the first data entry, a review (Review A) is carried out by another study employee.

Qualitative process evaluation: The audio files of interviews and focus groups will be sent to a professional writing office for transcription. Subsequently, the pseudonymization is carried out by employees of the PE team.

**Total duration of the study**

The PRECOVERY study is funded by the Innovation Fund of the Federal Joint Committee in the period from 01.11.2022 to 31.10.2026. A timetable with the most important key data can be found in the table below (details in the "GANTT Chart", Appendix I).

**Table 6**. PRECOVERY schedule overview

| | |
|---|---|
| Recruitment of the first patient (FPFV) | 2. Quarter 2023 |
| Recruitment of the last patient | 2. Quarter 2024 |
| Last patient out | 3. Quarter 2025 |
| Final statistical analysis | 1. Quarter 2026 |
| Study duration per patient | 14 Months |
| Planned interim analysis(s) | According to 25% of recruited patients |

# 9 Risk-benefit assessment

Patients are expected to benefit from prehabilitation because they receive intensified, multi-professional, preoperative preparation before elective cardiac procedures in experienced rehabilitation centers, feel less anxiety and distress, and achieve an improvement in functional capacity. Furthermore, an improvement in preoperative expectations and better education of patients and their families is expected. Individualized and optimized preoperative medical care through sports and exercise therapy as well as occupational therapy measures, supportive discussions, training courses and discussions with relatives can presumably optimize the preparation of patients for cardiac procedures, reduce the rate of perioperative complications and improve the quality of life in the postoperative course. In addition, patients have personal contact persons through daily contact with the prehabilitation team.

Participation in the study does not pose any discernible risk to patients. The 2-week, inpatient or semi-inpatient prehabilitation takes place during the period in which the patients (sometimes full of anxiety and without direct medical support) have to wait for several weeks in the home environment for the elective cardiac procedure. The sports and exercise therapy measures are individually adapted to the patient's performance. Each patient receives a stress ECG on the day of admission or the following day at the prehabilitation center. On the basis of this ECG, the individual training load, which is free of symptoms and findings, is determined. Studies demonstrate the feasibility and safety of a preoperative sports and exercise program with mild to moderate intensity in patients prior to elective cardiac surgery (Steinmetz et al. 2022; Arthur et al. 2000; Sawatzky et al. 2014; Stammers et al. 2015). In addition, the S-3 guideline on cardiac rehabilitation is applied in German-speaking Europe (S-3 guideline, LL-KardReha, 2020). ECG monitoring during aerobic endurance training, for example, also provides additional security. The precautions and contraindications of the individual forms of training are described in detail in the treatment manual (see appendix). If, despite all safety incidents, complaints such as pectanginous symptoms, cardiac arrhythmias, dizziness, malaise, fatigue or muscular problems occur, the first thing to do is to reduce the training intensity and stop training if the symptoms remain the same. Appropriate documentation and subsequent examination by the study physician will be ordered. The risk of falls in the context of training interventions is counteracted by a close patient-therapist connection (sometimes 1:1 care). The specialists also have in-depth knowledge of fall prevention. We do not expect any serious side effects from the training intervention, such as fatigue or an increased risk of mortality. For example, Nashimoto et al. (Nashimoto et al. 2022) investigated the safety of exercise loads in elderly patients with severe aortic stenosis in their review. Seven studies that included data from exercise ECGs and 16 other studies that included preoperative physical examinations prior to TAVI intervention were evaluated. Authors concluded that adequate physical exertion

is unlikely to produce hemodynamic changes that can lead to death (Nashimoto et al. 2022). Unfavourable side effects of supportive conversations, discussions with relatives, training or occupational therapy have not yet been described. The psychological interviews are conducted by psychologists who have completed bachelor's, diploma and master's degrees or licensed physicians. Any psychological stress that may occur before the elective cardiac procedure can be adequately absorbed. The possible occurrence of severe depression or suicidality in the course of the disease will be recorded by an appropriate SOP and addressed therapeutically as needed. In the pre-hospital assessments, it is to be expected that up to 40% of older people in the hospital may have mild or more severe cognitive disorders in this patient group. Patients must be informed that dementia could be suspected during the examinations and that this can have consequences for the self-image of the person concerned. If a mild cognitive disorder or incipient dementia is revealed in screening, there is not yet a restriction of the ability to consent and legal capacity. According to Vollmann, this only exists during the transition to more severe dementia (MMST < 15 p., MoCA < 8 p.). Patients with severe dementia will not be included in the study. In the presence of cognitive disorders, patients and their relatives offered options for further medical clarification and, if necessary, further appointments will be arranged. Randomization does not result in any health disadvantages, as patients in the control group receive the usual standard treatment. With regard to the psychometric survey as well as the interventions, the consent of the patients will be obtained. The preoperative interview and anamnestic survey requires about 1.5 to 2.5 hours. Since the planned psychometric instruments of this study design are common examination procedures in everyday clinical practice, we assume a low level of burden and risk for the study participants. Only generally accepted questionnaire procedures established in everyday clinical practice are used and great importance has been placed upon data protection and pseudonymized storage of personal data. Blood sampling should be taken before and after the cardiac procedure (t0, t2, t3, t6) primarily as part of the routinely performed basic diagnostics, so that unnecessary punctures and a large loss of time can be minimized. Blood sampling to obtain cellular and non-cellular blood components such as serum, plasma or mononuclear cells from whole blood is now considered a routine procedure in medicine. The risk of infections (thrombophlebitis), major bleeding or nerve injuries can be classified as extremely low if carried out properly. The most common, but harmless and temporary side effect is a hematoma above the puncture site due to postoperative bleeding or injury to smaller vessels. This has no functional consequences and is perceived as aesthetically disturbing at best.

**Abandonment criteria**

Any study patient can terminate his/her participation in the study at any time without giving reasons or revoke his/her consent to participate in the study. The responsible study director

(Prof. Dr. med. Christine von Arnim) and the investigators may also terminate participation in the study prematurely if adverse events preclude further participation in the study/implementation of the intervention (e.g. if the risks outweigh the benefits, or in the event of a violation of the study protocol). In the event of premature termination of study participation, the responsible investigator must inform the clinical coordinator of the coordinating study center (Göttingen) of the premature termination of the patient within one week by e-mail. Furthermore, both the investigator and the sponsor can terminate the study at a study site if unforeseen circumstances occur. In the event of termination or premature termination, the health insurance company concerned (AOK Lower Saxony or AOK Baden-Württemberg) will be informed immediately in order to terminate the routine health insurance data delivery. It will not be possible to carry out the research project if the funds for continuation are no longer available, if patient recruitment is inadequate or if serious problems arise with regard to the quality of the data collected.

# 10 Adverse Events (AE) and Serious Adverse Events (SAE)

**Definitions**

An adverse event (AE) is an adverse medical event (abnormal laboratory findings, symptom, disease) in a study patient that occurs during the course of the study but is not necessarily causally related to the study intervention. A serious adverse event (SAE) is any adverse event that results in any of the following:

- Death
- life-threatening situation (patient is in imminent danger of death),
- inpatient hospitalization in an acute hospital or extension of an existing hospital stay,
- persistent or significant disability,
- other, medically important condition: conditions that, in the opinion of the study doctor, may not be immediately life-threatening or lead to a hospital stay, but threaten the safety of the patient or require intervention to prevent any of the events mentioned.

**Capturing AEs and SAEs**

No drug or medical device will be tested as part of the PRECOVERY study. There is no legal obligation to report adverse events to the competent authority. A selection of adverse events will be recorded to monitor specific safety aspects of the study intervention and to evaluate the study outcome. After randomization, the following selection of undesirable events will be documented in the eCRF:

- Any adverse cardiovascular events
- Revascularization procedures or other invasive cardiac procedures
- All SAEs (cardiac and non-cardiac reasons)

The Data Safety Monitoring Board receives quarterly reports on the recorded selection of AEs and on the recorded SAEs (line listings).

# 11 Data Safety Monitoring Board (DSMB)

An independent Data Safety Monitoring Board will be set up prior to the inclusion of the first patient. The DSMB reviews the course of the study on a quarterly basis and determines whether the safety of the patients continues to be guaranteed and whether the unchanged continuation of the study is justifiable, or (if necessary) makes recommendations for discontinuation or modifications of the study. In order to fulfil this task, the DSMB receives information on protocol deviations, the status of patient recruitment and the observed SAEs (line listings).

# 12 Monitoring

A central study management team from the Clinical Trials Unit of the University Medical Center Göttingen (Head of Clinical Trials Unit: Ralf Tostmann) will accompany the implementation of the study. The study site operates according to ICH/GCP guidelines and the corresponding internal SOPs of the sponsor. Study sites and study documentation are reviewed during the course of the study by the sponsor or a representative designated by the sponsor (monitor, auditor) as a quality-assuring measure. In accordance with current GCP and ICH guidelines and sponsor SOPs, the monitor periodically reviews the source documents to ensure that the data captured in the CRF is accurate and reliable. The investigator shall provide the monitor and the sponsor's internal auditors with direct access to all source documents (study-related documents and relevant hospital or medical records) to confirm the data contained in the CRF. Source documents must be kept in accordance with the requirements of the ICH/GCP Directive. The monitor also checks compliance with the study protocol as well as the conduct of the study in accordance with ICH-GCP and the applicable regulatory requirements. Monitoring is carried out according to a risk-based approach and is defined in a study-specific monitoring manual, which also regulates other tasks and responsibilities. At least one initiation visit, one annual interim visit and one close-out visit are planned for each center. After monitoring, study centers will be informed about the results, possible deviations from the protocol, GCP or other requirements and, if necessary, are asked to take necessary measures. At the sponsor and at the study sites, the personnel involved will be trained prior to the start of the study on the indication to be treated, the work instructions to be used in this clinical trial, the protocol and all study-specific procedures before study-specific tasks are assumed and performed. For this purpose, a training protocol is kept in each study center.

# 13 Biometrics/Analysis

**Sample size estimation based on the primary outcome and relevant effect size**

A total of 422 patients are planned for the clinical trial. The sample size planning was confirmatory in order to be able to answer the primary questions with a power of 80%. A total of 338 patients (169 per treatment group) are sufficient to show a clinically relevant difference of 0.045 points in EQ-5D-5L to t6 with 80% power to the generally accepted two-sided significance level of 5%. It is assumed that the common standard deviation of the EQ-5D-5L is 0.17 and the observations on the covariables have an R-squared of 0.25. Since this is a longitudinal study, a dropout rate of up to 20% is assumed, so that a total of 422 patients are to be recruited. The sample size calculation was performed in nQuery version 8.

The determination of a clinically relevant difference to 0.045 was based on the publication by McClure et al. (2017), which limited a minimum clinically relevant difference to values between 0.037 and 0.069. Since the publication did not contain any results on the German population, a value was chosen that was about 10% lower than the average of the countries considered in the publication. The assumption of a standard deviation of 0.17 is based on observations by (McClure et al. 2018), who observed a standard deviation of 0.171 at baseline in a sample of 1927 diabetes-2 patients and a standard deviation of 0.170 in the 12-month follow-up.

**Process Evaluation**

The quantitative process evaluation will be carried out in all patients and related persons of the main study. In addition, the facility managers of all participating centers (n= 8) and the respective multipliers (n= 8). For the employee survey, a sample of 5 employees per facility is considered sufficient to obtain an indicative overview of the implementation of prehabilitation. During the training courses, all participants and the person providing the training will be interviewed.

In the qualitative process evaluation, interviews are conducted with 24 patients and 24 related persons (16 control group and 8 intervention group each). This number is usually sufficient to maintain a saturation of content.

If necessary, a subsequent recruitment would take place. In addition, one study physician is interviewed per recruiting center (n=9), this number seems sufficient for an overview of the assessment of the effects of prehabiliation, and it is also not foreseeable how many physicians per center will act as study physicians.

In addition, a focus group with all multipliers (n= 8) and two focus groups with approx. 8 employees of the prehabilitation centers each will be conducted. Two focus groups, each with 8 general practitioners, are also planned at the end of the study. The implementation of these

5 focus groups for the interviews provides a broad base of data to adequately answer the questions of the qualitative process evaluation.

**Planned statistical test procedures**

The primary analysis follows the intention-to-treat principle. Patients are evaluated in the group they were randomized to. A sensitivity analysis is based on the per-protocol population, which includes all patients without serious protocol deviations. For the evaluation of the main outcome (1-year mortality and EQ-5D-5L), a joint model for longitudinal data with the covariates age, sex, participation in the disease management program and center is taken into account (Król et al. 2016). Two-sided p-values less than 0.05 are considered statistically significant. Secondary outcomes are evaluated using generalized mixed linear models (with corresponding link function) and the covariates age, gender, baseline result, and center. The 30-day mortality is analyzed using Cox regression with the additional influencing factors sex, age and center. Treatment effects are always reported with a 95% confidence interval.

In order to minimize a possible reporting bias and to maintain the GCP conformity of the study, all planned statistical analyses (confirmatory and exploratory) are recorded in a statistical analysis plan (SAP) before the start of the evaluation. Furthermore, to ensure high data quality, a blinded data review is carried out before the database closes. This examines the feasibility of the analyses mentioned in SAP on the basis of the data collected.

**Process evaluation**

Quantitative process evaluation:

Exploratory sensitivity analyses will be performed to assess how program adherence, dose and framework conditions affect the effectiveness (primary endpoint) of the intervention. For this purpose, interaction terms between the treatment effect and the respective target of the quantitative process evaluation are included in the generalized mixed linear models described. In addition to the intention-to-treat analysis, an analysis population is defined that has started the intervention as intended and is also adherent (Starting and Adhering Estimate).

Qualitative process evaluation:

The evaluation will be carried out according to the structuring qualitative content analysis developed by Kuckartz (2016) with the help of the MAXQDA software. The analysis is carried out in parts deductively as well as inductively by 1-2 employees. Regular weekly meetings on progress will be held, at which content-related issues are also discussed, such as the creation of the code trees, the naming of the codes and the assignment of the text passages.

# 14 Data management and data protection

Details of data management (procedures, responsibilities, data corrections) have been detailed in a data management plan. The data will be entered remotely via an electronic case report form (eCRF) and stored pseudonymously in a provided secuTrial® database, an established GCP-compliant web solution. The data for the evaluation, which is stored in the secuTrial® database, is made available to the evaluating institutions via an encrypted e-mail program (Cryptshare). In addition to the data of the eCRF, pseudonymized routine health insurance data of the AOK will also be made available for health economic evaluation. This data will be transmitted to the evaluating institution via a secure connection. The identification of the study participants with the health insurance companies is carried out via the insurance number, which is collected when the patients are admitted to the study and transmitted to the respective AOK together with the study ID (Figure 4).

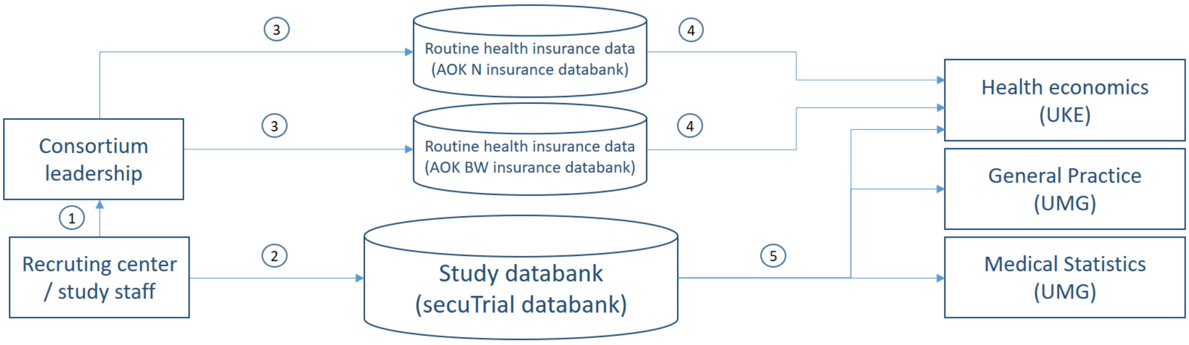


1. Transmission of a list of participants including study ID, health insurance number, data of participation, health insurance company. The data will be saved as a document on the restricted-access PRECOVERY Sharepoint (data cloud)
2. Transmission of study data with the study ID as an identifier in the secuTrial databank using Web-Access (HTTPS)
3. Transmission of a list of participants including study ID, health insurance number, data of participation from the consortium leadership to the AOK N or AOK BW using Cryptshare
4. Transmission of the AOK routine health insurance data using PRECOVERY study ID as an identifier. Transmission of data from the health insurance company to the Department of Health Economics (UKE) using Cryptshare (AOK N) and MOVEit (AOK BW)
5. Transmission of trial data stored in the secuTrial databank using the PRECOVERY study ID as an identifier to all of the evaluating organisations. The data will be made available via the restricted-access PRECOVERY Sharepoint (data cloud)

**Figure 4**. Data flow between recruiting centers and the PRECOVERY evaluation

Offsite checks of the data for plausibility and missing data will be carried out throughout the course of the study to ensure high data quality and to monitor recruitment. Inconsistent data will lead to queries and/or planned visits to verify source data (SDV) according to SOPs of the UMG study center. After publication of the primary results, access to the anonymous data for legitimate research purposes is guaranteed via a Data Access Committee in order to meet the FAIR criteria.

As far as possible, blood samples will be taken from all patients. The blood samples are not funded by the main sponsor (GBA). For this reason, this is covered by other public funding opportunities or by means of the participating clinics. Biomaterial extraction and processing takes place in accordance with the SOPs of the biomaterial bank of the Department of Geriatrics of the UMG. This corresponds to the SOPs of the German Center for Neurodegenerative Diseases and is largely congruent with other national and international collection strategies. Any deviation from the SOPs will be documented. There is no time limit for the storage of biomaterials. In order to ensure traceability, all steps carried out must be signed off on the sample and subject protocol and changes must be noted. These are noted with the same pseudonymization code and kept under lock and key. Deviations are also included in the electronic database. The data from evaluations of blood samples will be entered into the central database.

**Collection, storage (type, place, duration) and disclosure of data, ensuring data security, revocation and deletion**

The paper-based completed original questionnaires will be kept securely and destroyed after 10 years in accordance with the legal conditions of data protection. The password-protected PCs on which the data is processed and backed up and locked in the Clinic for Cardiothoracic and Vascular Surgery and the Clinic for Cardiology of the UMG. The database with the study data is located on local servers of UMG, which run on a RAID 10 system and are backed up daily. Daily backups are stored for 20 days according to SOPs of the Medical Informatics UMG. After deactivation of the database, a copy of the database is password-encrypted and archived within the UMG for 10 years. Patients are informed of their right to revoke their consent to the use of their data at any time and to request the deletion of personal data.

**Process evaluation**

The process evaluation will also be carried out in accordance with the current version of the Declaration of Helsinki. Here, too, the following apply:

The participation of the subjects in the study is voluntary. The consent can be withdrawn at any time, without giving reasons and with no disadvantage for the subject’s further medical care.

The names of the test persons and all other confidential information are subject to (medical) confidentiality and the provisions of the Federal Data Protection Act. Subject data will only be passed on in pseudonymised form. Third parties do not have access to the original documents.

Prior to the start of the study, participants will be informed in writing and orally about the nature and scope of the planned study, in particular about the possible benefits for their health and

possible risks. Their consent will be documented by signature on the consent form. In some cases, data collection is anonymous, and no signature on a declaration of consent is required. Pursuant to Art. 6 para. 1 lit. A of the GDPR, the collection and processing of personal data requires informed consent. Each study participant must give written consent before being included in the project. Prior to this, they will be informed by a member of the study team (study physician, outpatient clinic, research assistant, PE team) about the nature and scope of the project as well as data collection, data evaluation and data protection regulations in oral and written form. The information will be given in a form that is understandable for the respective study participants. These include, among other things, the contact details of the scientists, the purpose of the data collected, the recipients of the personal data, the criteria for the storage period of the data and the right to revoke consent.

The declaration of consent to participate in the study is dated and signed by the study participant and a member of the study team. In the case of on-site reconnaissance, the signatures are given at the same time. In the case of information by telephone, the signature of the informed person is first given, who sends both copies to the respective study center, and then receives an additional copy signed by the study employee by post before the start of the study. The other copy is stored in a locked cabinet at the respective study center.

The data collected will be stored and evaluated exclusively for the purpose of conducting the study. The audio files will be deleted after the end of the study. The contact details of the test persons will be destroyed after completion of the data analysis.

In the case of data collection by means of interviews, the guidelines are designed in such a way that only the necessary information is collected to answer the research questions. The risk of exposing the study participants to psychological damage as a result of the interviews is minimized by the principle of "usual mood swings in everyday life". Accordingly, it is justifiable from a research ethics point of view to ask the study participants about difficult topics that can be assumed that they would also find a topic in their everyday lives.

The collected data is recorded on paper case sheets or electronic data storage devices, treated as strictly confidential and only encrypted without naming (pseudonymized) and transmitted with a password. Access to the original documents will be denied to third parties. All data collected from participants is pseudonymized using numerical and letter codes. A retrospective assignment to a person is possible only with the help of a "key", which is kept in the study center. This "key" is a list that is kept in a locked cabinet and is only accessible to the project managers or their representatives. The pseudonymized data is only stored in encrypted form on the server of the participating study centers.

Personal data (real name, ID and contact details) of the patients who agree to be interviewed will be sent by phone, fax or encrypted e-mail from the recruiting center to a member of the PE team. Patients are informed about this. In the same way, the employee of the PE team receives

the contact details, name and ID of all patients in the intervention group so that the standardized interview can be carried out by a member of the PE team after the prehabiliation. This personal data is also stored in a locked cabinet (separate cabinet from the one in which the survey data is kept), the key is with the project manager or a representative. The original recordings of the interviews are stored only on an encrypted and password-protected data storage medium, which is locked in a separate cabinet and not on a server or a computer. The transcription is carried out directly from the data storage medium by employees of the institutes on site. The audio files can also be passed on to a specialist company for writing. The transmission of the audio files as well as their writing themselves will be carried out in accordance with the strict data protection guidelines pursuant to Art. 32 GDPR, which are contractually stipulated (cf. data protection concept). The audio files will then be destroyed by the specialist company.

The audio recordings, contact details and pseudonymization lists will be deleted after the end of the study. All other data (transcripts, protocols, demographic sheets, etc.) will be stored for 10 years and then destroyed. The deletion of all documents will be carried out on the basis of a deletion protocol.

**Quality assurance**

GDPR-compliant conduct of the study is ensured by the supervision of the data protection officer of the University Medical Center Göttingen (Robert-Koch-Straße 40, 37075 Göttingen, datenschutz@med.uni-goettingen.de).

MAXQDA is a licensed software for qualitative and mixed methods research used in qualitative process evaluation. The use of the software ensures that the quality of the various content analysis evaluation steps will be increased by the privacy-secure possibility of digitally encoding pseudonymized data in the study team and visualizing it with diagrams. The digital participation of several analysts in the analysis of a data set also increases the quality of the findings in a qualitative study.

# 15 Ethical concerns, legal and administrative regulations

The study will be conducted in accordance with the ethical principles laid down in the Declaration of Helsinki (May 2003) (World Medical Association 2013).
The study protocol will be submitted with the necessary further documents to the responsible ethics committee of the University Medical Centre Göttingen for review and evaluation. The study does not begin until the ethics committee has approved it.
After receiving the positive vote of the Ethics Committee of the University Medical Center Göttingen, the project will be reported to the ethics committees responsible for the other recruitment centers or approval evaluations of these ethics committees will be requested.
Changes or additions to the study protocol can only be initiated and authorized by the consultant management. The Ethics Committee of the University Medical Center Göttingen will be informed about changes to the study protocol. If necessary, an affirmative assessment will be requested again. Substantial changes may not be implemented before the decision of the ethics committee.
The planned study is not subject to any legal specifics outside the usual legal framework. In particular, it is not subject to the regulations of pharmaceutical or medical device law.

# 16 Handling of biosamples

Within the scope of this study, up to a maximum of 50ml of blood will be collected per study visit (t0, t2, t3, t6) and their cellular and non-cellular components will be processed and stored. The study has been initially designed as a prospective single-sample survey and longitudinal data collection. In addition to neurodegenerative, inflammatory and cardiac markers, genomic and epigenetic markers (e.g. of the ApoE genotype) are also investigated. In addition, samples in the COVID-19 Research Network Lower Saxony (COFONI) will be examined to determine the extent to which vaccination or disease status and immune response are associated with clinical data and course (long COVID).

The processing and storage of the samples (serum, plasma, RNA from PAXgene tubes) are carried out according to the locally established standard operating procedures in the Department of Geriatrics of the University Medical Center Göttingen. Serum and plasma are stored briefly at 4°C. The serum is stored for 45 minutes for coagulation. Serum and EDTA plasma are centrifuged at 2,000 x g for 10 minutes and stored as 300 µL aliquots at -80 °C. The cell pellets from the EDTA tubes are reconstituted in PBS and stored at -80 °C for DNA extraction. The PAXgene tube is stored at room temperature for 120 minutes, followed by 24 hours at -20 °C and long-term storage at -80 °C until use. With the help of various measurement methods, biomarkers for the assessment of preoperative/interventional risk as well as peri- and postoperative/interventional complications will be identified and validated. The test persons are explicitly informed that, if necessary, a genetic examination of the donated samples will be carried out – in compliance with pseudonymization or anonymization. In order to protect the subjects, no feedback is given on individual markers, especially predictive markers.

It has been planned to determine the following parameters in the blood:
Neurofilament light chain (NfL), pospho-tau181/217, total tau, Aβ-peptides, NSE, s100 protein, ApoE genotype, GDF-15, hsTNT, NTproBNP, ferritin, vitamin B12, folic acid, vitamin D, ACTH, HIF1-alpha, cytokines and chemokines (e.g. IL1RA, IL-10, IL-1β, IL-4, IL-6, ICAM, VCAM, TNF-alpha), neural and paraneoplastic autoantibodies, normetanephrine, metanephrine. Epigenetic analyses (miR-132 RNA, miR-192 RNA, miR-181 RNA, etc., associated with MCI and Alzheimer's disease), SARS-Cov-2 antibodies.

The determination of further parameters can be carried out after successful technical validation of the methodology and existing funding. Applications for analysis can be submitted by study

participants or external cooperation partners. An assessment is carried out by a Use and Access Committee with representatives from the centers participating in the sample collection. Patients can also decide whether the collected biosamples should also be available for evaluation with other cooperation partners.

Since the blood-based biomarkers are valuable data and samples that will be of great value for research, no destruction of the samples in the biobank per se has been planned. If a patient revokes participation in the study, he or she can decide for himself whether biomaterial samples that have already been stored may either be used anonymously or must be deleted/destroyed. Research results that have already been achieved will not be destroyed, provided that the data are available in aggregated form or have already been published in aggregated form.

# 17 Subjects' insurance, commuting accident insurance

PRECOVERY does not provide for participant insurance or commuting accident insurance.

# 18 Publication rules

After approval of the ethics application by the lead ethics committee in Göttingen, the study protocol will be submitted for publication in an international peer-reviewed journal. Reporting guidelines (see www.equator-network.org) such as the CONSORT instruction and its adaptation for non-pharmacological and psychosocial intervention studies will be taken into account when preparing publications on the results of this study.

A publication charter was developed by the consortium management and the Steering Committee and takes into account the rights of each other. Obligations are laid down in the consortium agreements. In the publication strategy, the roles of the authors and the requirements for authorship have been defined in accordance with good scientific practice and quality. Planned publications have also been defined there and the procedure for further publication applications has been set out.

# 19 Literaturverzeichnis

Afilalo, Jonathan; Eisenberg, Mark J.; Morin, Jean-François; Bergman, Howard; Monette, Johanne; Noiseux, Nicolas et al. (2010): Gait speed as an incremental predictor of mortality and major morbidity in elderly patients undergoing cardiac surgery. In: *Journal of the American College of Cardiology* 56 (20), S. 1668–1676. DOI: 10.1016/j.jacc.2010.06.039.

Alberts, Jannis; Löwe, Bernd; Glahn, Maja Alicia; Petrie, Keith; Laferton, Johannes; Nestoriuc, Yvonne; Shedden-Mora, Meike (2020): Development of the generic, multidimensional Treatment Expectation Questionnaire (TEX-Q) through systematic literature review, expert surveys and qualitative interviews. In: *BMJ open* 10 (8), e036169. DOI: 10.1136/bmjopen-2019-036169.

Alifier, Marek; Olsson, Bob; Andreasson, Ulf; Cullen, Nicholas C.; Czyżewska, Jolanta; Jakubów, Piotr et al. (2020): Cardiac Surgery is Associated with Biomarker Evidence of Neuronal Damage. In: *Journal of Alzheimer's disease : JAD* 74 (4), S. 1211–1220. DOI: 10.3233/JAD-191165.

Arai, Y.; Kudo, K.; Hosokawa, T.; Washio, M.; Miura, H.; Hisamichi, S. (1997): Reliability and validity of the Japanese version of the Zarit Caregiver Burden interview. In: *Psychiatry and Clinical Neurosciences* 51 (5), S. 281–287. DOI: 10.1111/j.1440-1819.1997.tb03199.x.

Arthur et al. (2000): Endoglin, an Ancillary TGFb Receptor, Is Required for Extraembryonic Angiogenesis and Plays a Key Role in Heart Development. In: *Developmental Biology* (217), S. 42–53. Online verfügbar unter 10.1006, zuletzt geprüft am 01.12.2022.

ATS Committee (2002): ATS statement: guidelines for the six-minute walk test. In: *American journal of respiratory and critical care medicine* 166 (1), S. 111–117. DOI: 10.1164/ajrccm.166.1.at1102.

Auer, Charlotte J.; Glombiewski, Julia A.; Doering, Bettina K.; Winkler, Alexander; Laferton, Johannes A. C.; Broadbent, Elizabeth; Rief, Winfried (2016): Patients' Expectations Predict Surgery Outcomes: A Meta-Analysis. In: *International journal of behavioral medicine* 23 (1), S. 49–62. DOI: 10.1007/s12529-015-9500-4.

Auer, Charlotte J.; Laferton, Johannes A. C.; Shedden-Mora, Meike C.; Salzmann, Stefan; Moosdorf, Rainer; Rief, Winfried (2017): Optimizing preoperative expectations leads to a shorter length of hospital stay in CABG patients: Further results of the randomized controlled PSY-HEART trial. In: *Journal of Psychosomatic Research* 97, S. 82–89. DOI: 10.1016/j.jpsychores.2017.04.008.

Balog, Piroska; Konkolÿ Thege, Barna (2019): The role of vital exhaustion in predicting the recurrence of vascular events: A longitudinal study. In: *International journal of clinical and health psychology : IJCHP* 19 (1), S. 75–79. DOI: 10.1016/j.ijchp.2018.11.004.

Bandura, Albert (Hg.) (1979): Sozial-kognitive Lerntheorie. 1. Aufl. Stuttgart: Klett-Cotta (Konzepte der Humanwissenschaften).

Barefoot, John C.; Brummett, Beverly H.; Williams, Redford B.; Siegler, Ilene C.; Helms, Michael J.; Boyle, Stephen H. et al. (2011): Recovery expectations and long-term prognosis of patients with coronary heart disease. In: *Archives of internal medicine* 171 (10), S. 929–935. DOI: 10.1001/archinternmed.2011.41.

Beatty, Alexis L.; Schiller, Nelson B.; Whooley, Mary A. (2012): Six-minute walk test as a prognostic tool in stable coronary heart disease: data from the heart and soul study. In: *Archives of internal medicine* 172 (14), S. 1096–1102. DOI: 10.1001/archinternmed.2012.2198.

Billmann, A. I. (2009): Prädiktive Bedeutung somatischer und psychosozialer Risikofaktoren hinsichtlich der Lebensqualität zwei Monate nach Myokardinfarkt. Universität zu Lübeck. Online verfügbar unter https://www.zhb.uni-luebeck.de/epubs/ediss732.pdf, zuletzt geprüft am 01.12.2022.

Bjarnason-Wehrens, Birna; Held, Klaus; Hoberg, Eike; Karoff, Marthin; Rauch, Bernhard (2007): Deutsche Leitlinie zur Rehabilitation von Patienten mit Herz-Kreislauferkrankungen (DLL-KardReha). In: *Clin Res Cardiol Suppl* 2 (3), S. 1–54. DOI: 10.1007/s11789-007-0001-0.

Bjelland, Ingvar; Dahl, Alv A.; Haug, Tone Tangen; Neckelmann, Dag (2002): The validity of the Hospital Anxiety and Depression Scale. In: *Journal of Psychosomatic Research* 52 (2), S. 69–77. DOI: 10.1016/S0022-3999(01)00296-3.

Blumenstein, J.; Möllmann, H.; Bleiziffer, S.; Bauer, T.; Ensminger, S.; Bekeredjian, R. et al. (2020): Transcatheter aortic valve implantation in nonagenarians: insights from the German Aortic Valve Registry (GARY). In: *Clinical research in cardiology : official journal of the German Cardiac Society* 109 (9), S. 1099–1106. DOI: 10.1007/s00392-020-01601-4.

Braun, M.; Scholz, U.; Hornung, R.; Martin, M. (2010): Die subjektive Belastung pflegender Ehepartner von Demenzkranken. Hinweise zur Validität der deutschen Version des Zarit Burden Interviews. In: *Z Gerontol Geriat* 43 (2), S. 111–119. DOI: 10.1007/s00391-010-0097-6.

Broadbent, Elizabeth; Petrie, Keith J.; Main, Jodie; Weinman, John (2006): The brief illness perception questionnaire. In: *Journal of Psychosomatic Research* 60 (6), S. 631–637. DOI: 10.1016/j.jpsychores.2005.10.020.

Busch, John C.; Lillou, Dorothea; Wittig, Godehard; Bartsch, Petra; Willemsen, Detlev; Oldridge, Neil; Bjarnason-Wehrens, Birna (2012): Resistance and balance training improves functional capacity in very old participants attending cardiac rehabilitation after coronary bypass surgery. In: *Journal of the American Geriatrics Society* 60 (12), S. 2270–2276. DOI: 10.1111/jgs.12030.

Büsching, Gilbert (2015): Short Physical Performance Battery Test – Ein Muss in der Geriatrie. In: *physiopraxis* 13 (01), S. 42–43. DOI: 10.1055/s-0034-1399816.

Buysse, D. J.; Reynolds, C. F.; Monk, T. H.; Berman, S. R.; Kupfer, D. J. (1989): The Pittsburgh Sleep Quality Index: a new instrument for psychiatric practice and research. In: *Psychiatry research* 28 (2), S. 193–213. DOI: 10.1016/0165-1781(89)90047-4.

Cabilan, C. J.; Hines, Sonia; Munday, Judy (2016): The Impact of Prehabilitation on Postoperative Functional Status, Healthcare Utilization, Pain, and Quality of Life: A Systematic Review. In: *Orthopedic nursing* 35 (4), S. 224–237. DOI: 10.1097/NOR.0000000000000264.

Chan, Carol K.; Song, Yiqing; Greene, Ryan; Lindroth, Heidi; Khan, Sikandar; Rios, Gabriel et al. (2021): Meta-analysis of ICU Delirium Biomarkers and Their Alignment With the NIA-AA Research Framework. In: *American journal of critical care : an official publication, American Association of Critical-Care Nurses* 30 (4), S. 312–319. DOI: 10.4037/ajcc2021771.

Chan, T. S. F.; Lam L C W; Chiu H F K (2005): Validation of the Chinese version of the Zarit Burden Interview. In: *Hong Kong Journal of Psychiatry* (15), S. 9–13.

Chattat, Rabih; Cortesi, Valeria; Izzicupo, Fabio; Del Re, Maria Letizia; Sgarbi, Cinzia; Fabbo, Andrea; Bergonzini, Elisa (2011): The Italian version of the Zarit Burden Interview: a validation study. In: *Int. Psychogeriatr.* 23 (5), S. 797–805. DOI: 10.1017/s1041610210002218.

Church, Sophie; Rogers, Emily; Rockwood, Kenneth; Theou, Olga (2020): A scoping review of the Clinical Frailty Scale. In: *BMC Geriatr* 20 (1), S. 393. DOI: 10.1186/s12877-020-01801-7.

Cruz-Jentoft, Alfonso J.; Bahat, Gülistan; Bauer, Jürgen; Boirie, Yves; Bruyère, Olivier; Cederholm, Tommy et al. (2019): Sarcopenia: revised European consensus on definition and diagnosis. In: *Age Ageing* 48 (1), S. 16–31. DOI: 10.1093/ageing/afy169.

Deiner, Stacie; Baxter, Mark G.; Mincer, Joshua S.; Sano, Mary; Hall, James; Mohammed, Ismail et al. (2020): Human plasma biomarker responses to inhalational

general anaesthesia without surgery. In: *British journal of anaesthesia* 125 (3), S. 282–290. DOI: 10.1016/j.bja.2020.04.085.

Desrosiers, J.; Bravo, G.; Hébert, R.; Dutil, E. (1995): Normative data for grip strength of elderly men and women. In: *The American journal of occupational therapy : official publication of the American Occupational Therapy Association* 49 (7), S. 637–644. DOI: 10.5014/ajot.49.7.637.

Deutsche Gesellschaft für Geriatrie e.V. (2020): Deutsche Übersetzung der Klinische Frailty Scale. Online verfügbar unter https://www.dggeriatrie.de/images/Bilder/PosterDownload/200331_DGG_Plakat_A4_Clinical_Frailty_Scale_CFS.pdf.

Deutsche Herzstiftung (2022): Herzbericht 2021. Online verfügbar unter https://www.herzstiftung.de/system/files/2022-09/DHB21-Herzbericht-2021.pdf.

DiMeglio, Matthew; Furey, William; Hajj, Jihane; Lindekens, Jordan; Patel, Saumil; Acker, Michael et al. (2019): Observational study of long-term persistent elevation of neurodegeneration markers after cardiac surgery. In: *Scientific reports* 9 (1), S. 7177. DOI: 10.1038/s41598-019-42351-2.

Doering, Lynn V.; Chen, Belinda; McGuire, Anthony; Bodán, Rebecca Cross; Irwin, Michael R. (2014): Persistent depressive symptoms and pain after cardiac surgery. In: *Psychosomatic medicine* 76 (6), S. 437–444. DOI: 10.1097/PSY.0000000000000074.

Doi, Y.; Minowa, M.; Uchiyama, M.; Okawa, M.; Kim, K.; Shibui, K.; Kamei, Y. (2000): Psychometric assessment of subjective sleep quality using the Japanese version of the Pittsburgh Sleep Quality Index (PSQI-J) in psychiatric disordered and control subjects. In: *Psychiatry research* 97 (2-3), S. 165–172. DOI: 10.1016/s0165-1781(00)00232-8.

Döring, N.; Bortz, J. (1993): Psychometrische Einsamkeitsforschung: Deutsche Neukonstruktion der UCLA Loneliness Scale. In: *Diagnostica* (3), S. 224–239. Online verfügbar unter https://psycnet.apa.org/record/1994-85522-001.

Du, Huiyun; Newton, Phillip J.; Salamonson, Yenna; Carrieri-Kohlman, Virginia L.; Davidson, Patricia M. (2009): A review of the six-minute walk test: its implication as a self-administered assessment tool. In: *European journal of cardiovascular nursing* 8 (1), S. 2–8. DOI: 10.1016/j.ejcnurse.2008.07.001.

Eder, B.; Hofmann, P.; Hödl, R.; Wonisch, M.; Schmid, P.; Pokan R. (2013): Ist die kardiale Rehabilitation beim alten Menschen noch effektiv? In: *Journal für Kardiologie* (20), S. 262–268. Online verfügbar unter https://scholar.archive.org/work/72qt4f5ckvcrlmfdua36y56434/access/wayback/http://www.kup.at/kup/pdf/11829.pdf.

Ehrensperger, Michael M.; Berres, Manfred; Taylor, Kirsten I.; Monsch, Andreas U. (2010): Screening properties of the German IQCODE with a two-year time frame in MCI and early Alzheimer's disease. In: *Int. Psychogeriatr.* 22 (1), S. 91–100. DOI: 10.1017/S1041610209990962.

Ellis, Jeffrey J.; Eagle, Kim A.; Kline-Rogers, Eva M.; Erickson, Steven R. (2005): Validation of the EQ-5D in patients with a history of acute coronary syndrome. In: *Current Medical Research and Opinion* 21 (8), S. 1209–1216. DOI: 10.1185/030079905X56349.

Enright, P. L.; Sherrill, D. L. (1998): Reference equations for the six-minute walk in healthy adults. In: *American journal of respiratory and critical care medicine* 158 (5 Pt 1), S. 1384–1387. DOI: 10.1164/ajrccm.158.5.9710086.

Evered, Lisbeth; Silbert, Brendan; Scott, David A.; Zetterberg, Henrik; Blennow, Kaj (2018): Association of Changes in Plasma Neurofilament Light and Tau Levels With Anesthesia and Surgery: Results From the CAPACITY and ARCADIAN Studies. In: *JAMA neurology* 75 (5), S. 542–547. DOI: 10.1001/jamaneurol.2017.4913.

Fiorina, Claudia; Vizzardi, Enrico; Lorusso, Roberto; Maggio, Marcello; Cicco, Giuseppe de; Nodari, Savina et al. (2007): The 6-min walking test early after cardiac surgery. Reference values and the effects of rehabilitation programme. In: *European journal of cardio-thoracic surgery : official journal of the European Association for Cardio-thoracic Surgery* 32 (5), S. 724–729. DOI: 10.1016/j.ejcts.2007.08.013.

Fong, Tamara G.; Davis, Daniel; Growdon, Matthew E.; Albuquerque, Asha; Inouye, Sharon K. (2015): The interface between delirium and dementia in elderly adults. In: *The Lancet. Neurology* 14 (8), S. 823–832. DOI: 10.1016/S1474-4422(15)00101-5.

Fried, L. P.; Tangen, C. M.; Walston, J.; Newman, A. B.; Hirsch, C.; Gottdiener, J. et al. (2001): Frailty in older adults: evidence for a phenotype. In: *The journals of gerontology. Series A, Biological sciences and medical sciences* 56 (3), M146-56. DOI: 10.1093/gerona/56.3.m146.

Fuchs, Simon; Bode, Leonie; Ernst, Jutta; Marquetand, Justus; Känel, Roland von; Böttger, Sönke (2020): Delirium in elderly patients: Prospective prevalence across hospital services. In: *General hospital psychiatry* 67, S. 19–25. DOI: 10.1016/j.genhosppsych.2020.08.010.

Geerlings, M. I.; Jonker, C.; Bouter, L. M.; Adèr, H. J.; Schmand, B. (1999): Association between memory complaints and incident Alzheimer's disease in elderly people with normal baseline cognition. In: *The American journal of psychiatry* 156 (4), S. 531–537. DOI: 10.1176/ajp.156.4.531.

Gentili, A.; Weiner, D. K.; Kuchibhatla, M.; Edinger, J. D. (1995): Test-retest reliability of the Pittsburgh sleep quality index in nursing home residents. In: *Journal of the American Geriatrics Society* 43 (11), S. 1317–1318. DOI: 10.1111/j.1532-5415.1995.tb07415.x.

Glaesmer, Heide; Hoyer, Jürgen; Klotsche, Jens; Herzberg, Philipp Yorck (2008): Die deutsche Version des Life-Orientation-Tests (LOT-R) zum dispositionellen Optimismus und Pessimismus. In: *Zeitschrift für Gesundheitspsychologie* 16 (1), S. 26–31. DOI: 10.1026/0943-8149.16.1.26.

Guigoz, Yves; Lauque, Sylvie; Vellas, Bruno J. (2002): Identifying the elderly at risk for malnutrition. The Mini Nutritional Assessment. In: *Clinics in geriatric medicine* 18 (4), S. 737–757. DOI: 10.1016/s0749-0690(02)00059-9.

Guralnik, J. M.; Simonsick, E. M.; Ferrucci, L.; Glynn, R. J.; Berkman, L. F.; Blazer, D. G. et al. (1994): A short physical performance battery assessing lower extremity function: association with self-reported disability and prediction of mortality and nursing home admission. In: *Journal of gerontology* 49 (2), M85-94. DOI: 10.1093/geronj/49.2.m85.

Hall, Daniel E.; Arya, Shipra; Schmid, Kendra K.; Carlson, Mark A.; Lavedan, Pierre; Bailey, Travis L. et al. (2017): Association of a Frailty Screening Initiative With Postoperative Survival at 30, 180, and 365 Days. In: *JAMA surgery* 152 (3), S. 233–240. DOI: 10.1001/jamasurg.2016.4219.

Helfferich, Cornelia (2022): Leitfaden- und Experteninterviews. In: Nina Baur und Jörg Blasius (Hg.): Handbuch Methoden der empirischen Sozialforschung. Wiesbaden: Springer Fachmedien Wiesbaden, S. 875–892.

Herrmann, Christoph (1997): International experiences with the Hospital Anxiety and Depression Scale-A review of validation data and clinical results. In: *Journal of Psychosomatic Research* 42 (1), S. 17–41. DOI: 10.1016/S0022-3999(96)00216-4.

Howard Leventhal; David R. Nerenz; David J. Steele (2020): Illness Representations and Coping With Health Threats. In: Andrew Baum, Shelley E. Taylor und Jerome E. Singer (Hg.): Handbook of psychology and health. (Volume IV) : social psychological aspects of health. London, New York: Routledge Taylor & Francis Group (Psychology revivals), S. 219–252. Online verfügbar unter https://www.taylorfrancis.com/chapters/edit/10.4324/9781003044307-9/illness-representations-coping-health-threats-howard-leventhal-david-nerenz-david-steele.

Hoyer, Jürgen; Eifert, Georg H.; Einsle, Franziska; Zimmermann, Katrin; Krauss, Stefanie; Knaut, Michael et al. (2008): Heart-focused anxiety before and after cardiac surgery. In: *Journal of Psychosomatic Research* 64 (3), S. 291–297. DOI: 10.1016/j.jpsychores.2007.09.009.

Hulzebos, E. H. J.; van Meeteren, N. L. U. (2016): Making the elderly fit for surgery. In: *The British journal of surgery* 103 (4), S. 463. DOI: 10.1002/bjs.10134.

Hurst, N. P.; Kind, P.; Ruta, D.; Hunter, M.; Stubbings, A. (1997): Measuring health-related quality of life in rheumatoid arthritis: validity, responsiveness and reliability of EuroQol (EQ-5D). In: *British journal of rheumatology* 36 (5), S. 551–559. DOI: 10.1093/rheumatology/36.5.551.

Jerusalem, M.; Schwarzer, R. (2003): SWE - Skala zur Allgemeinen Selbstwirksamkeitserwartung. Unter Mitarbeit von Leibniz Institut für Psychologie (ZPID).

Jessen, Frank; Wiese, Birgitt; Bachmann, Cadja; Eifflaender-Gorfer, Sandra; Haller, Franziska; Kölsch, Heike et al. (2010): Prediction of dementia by subjective memory impairment: effects of severity and temporal association with cognitive impairment. In: *Archives of general psychiatry* 67 (4), S. 414–422. DOI: 10.1001/archgenpsychiatry.2010.30.

Jorm, Anthony F. (2004): The Informant Questionnaire on cognitive decline in the elderly (IQCODE): a review. In: *Int. Psychogeriatr.* 16 (3), S. 275–293. DOI: 10.1017/s1041610204000390.

Jorm, Anthony F.; Scott, Ruth; Jacomb, Patricia A. (1989): Assessment of cognitive decline in dementia by informant questionnaire. In: *Int. J. Geriat. Psychiatry* 4 (1), S. 35–39. DOI: 10.1002/gps.930040109.

Juergens, Meike C.; Seekatz, Bettina; Moosdorf, Rainer G.; Petrie, Keith J.; Rief, Winfried (2010): Illness beliefs before cardiac surgery predict disability, quality of life, and depression 3 months later. In: *Journal of Psychosomatic Research* 68 (6), S. 553–560. DOI: 10.1016/j.jpsychores.2009.10.004.

Kaiser, M. J.; Bauer, J. M.; Ramsch, C.; Uter, W.; Guigoz, Y.; Cederholm, T. et al. (2009): Validation of the Mini Nutritional Assessment short-form (MNA-SF): a practical tool for identification of nutritional status. In: *The journal of nutrition, health & aging* 13 (9), S. 782–788. DOI: 10.1007/s12603-009-0214-7.

KATZ, S.; FORD, A. B.; MOSKOWITZ, R. W.; JACKSON, B. A.; JAFFE, M. W. (1963): Studies of illness in the aged. The index of ADL: A standardized measure of Biological and psychosocial function. In: *JAMA* 185, S. 914–919. DOI: 10.1001/jama.1963.03060120024016.

Kondrup, Jens; Rasmussen, Henrik Højgaard; Hamberg, Ole; Stanga, Zeno (2003): Nutritional risk screening (NRS 2002): a new method based on an analysis of controlled clinical trials. In: *Clinical Nutrition* 22 (3), S. 321–336. DOI: 10.1016/s0261-5614(02)00214-5.

König, H. H.; Bernert, S.; Angermeyer, M. C. (2005): Gesundheitszustand der deutschen Bevölkerung: Ergebnisse einer repräsentativen Befragung mit dem EuroQol-Instrument. In: *Gesundheitswesen* 67 (3), S. 173–182. DOI: 10.1055/s-2005-857991.

Król, Agnieszka; Ferrer, Loïc; Pignon, Jean-Pierre; Proust-Lima, Cécile; Ducreux, Michel; Bouché, Olivier et al. (2016): Joint model for left-censored longitudinal data, recurrent events and terminal event: Predictive abilities of tumor burden for cancer evolution with application to the FFCD 2000-05 trial. In: *Biometrics* 72 (3), S. 907–916. DOI: 10.1111/biom.12490.

Kuckartz, Udo (2016): Qualitative Inhaltsanalyse. Methoden, Praxis, Computerunterstützung. 3. Aufl. Weinheim: Beltz.

Kundi, Harun; Popma, Jeffrey J.; Reynolds, Matthew R.; Strom, Jordan B.; Pinto, Duane S.; Valsdottir, Linda R. et al. (2019): Frailty and related outcomes in patients undergoing transcatheter valve therapies in a nationwide cohort. In: *European heart journal* 40 (27), S. 2231–2239. DOI: 10.1093/eurheartj/ehz187.

Kyle, U.G; Genton, Laurence; Hans, D.; Pichard, C. (2003): Validation of a bioelectrical impedance analysis equation to predict appendicular skeletal muscle mass (ASMM). In: *Clinical Nutrition* 22 (6), S. 537–543. DOI: 10.1016/S0261-5614(03)00048-7.

Lecher, Silke; Klapper, Bernadette; Koch, Uwe (2002): Hamburger Fragebogen zum Krankenhausaufenthalt (HFK) Ein Instrument zur Defizitanalyse aus Patientensicht. In: *Zeitschrift für Medizinische Psychologie* 11 (3), S. 129–138. Online verfügbar unter https://content.iospress.com/articles/zeitschrift-fur-medizinische-psychologie/zmp11-3-08.

Leventhal, H., Nerenz, D.R. and Steele, D.J. (1984): Illness Representations and Coping with Health Threats. Social Psychological Aspects of Health. In: *Handbook of Psychology and Health* (IV), S. 219–252, zuletzt geprüft am 01.12.2022.

Ludwig, Kristina; Schulenburg, J-Matthias Graf von der; Greiner, Wolfgang (2017): Valuation of the EQ-5D-5L with composite time trade-off for the German population - an exploratory study. In: *Health and quality of life outcomes* 15 (1), S. 39. DOI: 10.1186/s12955-017-0617-9.

Luis, Cheryl A.; Keegan, Andrew P.; Mullan, Michael (2009): Cross validation of the Montreal Cognitive Assessment in community dwelling older adults residing in the Southeastern US. In: *International Journal of Geriatric Psychiatry* 24 (2), S. 197–201. DOI: 10.1002/gps.2101.

Mai, Ralf (2003): Die Alten der Zukunft. Eine Bevölkerungsstatistische Analyse. Wiesbaden: VS Verlag fur Sozialwissenschaften GmbH (Schriftenreihe des

Bundesinstituts Für Bevölkerungsforschung BIB Ser, v.32). Online verfügbar unter https://ebookcentral.proquest.com/lib/kxp/detail.action?docID=6301099.

Marmelo, Filipe; Rocha, Vânia; Moreira-Gonçalves, Daniel (2018): The impact of prehabilitation on post-surgical complications in patients undergoing non-urgent cardiovascular surgical intervention: Systematic review and meta-analysis. In: *Eur. J. Prev. Cardiol.* 25 (4), S. 404–417. DOI: 10.1177/2047487317752373.

May, Carl R.; Mair, Frances; Finch, Tracy; MacFarlane, Anne; Dowrick, Christopher; Treweek, Shaun et al. (2009): Development of a theory of implementation and integration: Normalization Process Theory. In: *Implementation science : IS* 4, S. 29. DOI: 10.1186/1748-5908-4-29.

May, Carl R.; Mair, Frances S.; Dowrick, Christopher F.; Finch, Tracy L. (2007): Process evaluation for complex interventions in primary care: understanding trials using the normalization process model. In: *BMC Fam Pract* 8 (1). DOI: 10.1186/1471-2296-8-42.

McCann, Michael; Stamp, Nikki; Ngui, Ann; Litton, Edward (2019): Cardiac Prehabilitation. In: *Journal of cardiothoracic and vascular anesthesia* 33 (8), S. 2255–2265. DOI: 10.1053/j.jvca.2019.01.023.

McClure, Nathan S.; Sayah, Fatima Al; Ohinmaa, Arto; Johnson, Jeffrey A. (2018): Minimally Important Difference of the EQ-5D-5L Index Score in Adults with Type 2 Diabetes. In: *Value in health : the journal of the International Society for Pharmacoeconomics and Outcomes Research* 21 (9), S. 1090–1097. DOI: 10.1016/j.jval.2018.02.007.

McClure, Nathan S.; Sayah, Fatima Al; Xie, Feng; Luo, Nan; Johnson, Jeffrey A. (2017): Instrument-Defined Estimates of the Minimally Important Difference for EQ-5D-5L Index Scores. In: *Value in health : the journal of the International Society for Pharmacoeconomics and Outcomes Research* 20 (4), S. 644–650. DOI: 10.1016/j.jval.2016.11.015.

McIsaac, Daniel I.; Bryson, Gregory L.; van Walraven, Carl (2016): Association of Frailty and 1-Year Postoperative Mortality Following Major Elective Noncardiac Surgery: A Population-Based Cohort Study. In: *JAMA surgery* 151 (6), S. 538–545. DOI: 10.1001/jamasurg.2015.5085.

Moore, Graham F.; Audrey, Suzanne; Barker, Mary; Bond, Lyndal; Bonell, Chris; Hardeman, Wendy et al. (2015): Process evaluation of complex interventions: Medical Research Council guidance. In: *BMJ (Clinical research ed.)* 350, h1258. DOI: 10.1136/bmj.h1258.

Nashimoto, Satoshi; Inoue, Tatsuro; Hotta, Kazuki; Sugito, Yuichi; Iida, Susumu; Tsubaki, Atsuhiro (2022): The safety of exercise for older patients with severe aortic stenosis undergoing conservative management: A narrative review. In: *Physiological reports* 10 (8), e15272. DOI: 10.14814/phy2.15272.

Nasreddine, Ziad S.; Phillips, Natalie A.; Bédirian, Valérie; Charbonneau, Simon; Whitehead, Victor; Collin, Isabelle et al. (2005): The Montreal Cognitive Assessment, MoCA: a brief screening tool for mild cognitive impairment. In: *Journal of the American Geriatrics Society* 53 (4), S. 695–699. DOI: 10.1111/j.1532-5415.2005.53221.x.

Neumann, Franz-Josef; Sousa-Uva, Miguel; Ahlsson, Anders; Alfonso, Fernando; Banning, Adrian P.; Benedetto, Umberto et al. (2019): 2018 ESC/EACTS Guidelines on myocardial revascularization. In: *European heart journal* 40 (2), S. 87–165. DOI: 10.1093/eurheartj/ehy394.

Oldridge, Neil; Höfer, Stefan; McGee, Hannah; Conroy, Ronan; Doyle, Frank; Saner, Hugo (2014): The HeartQoL: part II. Validation of a new core health-related quality of life questionnaire for patients with ischemic heart disease. In: *Eur. J. Prev. Cardiol.* 21 (1), S. 98–106. DOI: 10.1177/2047487312450545.

Perera, Subashan; Mody, Samir H.; Woodman, Richard C.; Studenski, Stephanie A. (2006): Meaningful change and responsiveness in common physical performance measures in older adults. In: *Journal of the American Geriatrics Society* 54 (5), S. 743–749. DOI: 10.1111/j.1532-5415.2006.00701.x.

Petermann, Franz (2011): Hospital Anxiety and Depression Scale, Deutsche Version (HADS-D). In: *Zeitschrift für Psychiatrie, Psychologie und Psychotherapie* 59 (3), S. 251–253. DOI: 10.1024/1661-4747/a000077.

Puls, Miriam; Sobisiak, Bettina; Bleckmann, Annalen; Jacobshagen, Claudius; Danner, Bernhard C.; Hünlich, Mark et al. (2014): Impact of frailty on short- and long-term morbidity and mortality after transcatheter aortic valve implantation: risk assessment by Katz Index of activities of daily living. In: *EuroIntervention : journal of EuroPCR in collaboration with the Working Group on Interventional Cardiology of the European Society of Cardiology* 10 (5), S. 609–619. DOI: 10.4244/EIJY14M08_03.

Punt, Ilona M.; van der Most, Roel; Bongers, Bart C.; Didden, Anouk; Hulzebos, Erik H. J.; Dronkers, Jaap J.; van Meeteren, Nico L. U. (2017): Verbesserung des prä- und postoperativen Behandlungskonzepts : Große elektiv-chirurgische Eingriffe. In: *Bundesgesundheitsblatt, Gesundheitsforschung, Gesundheitsschutz* 60 (4), S. 410–418. DOI: 10.1007/s00103-017-2521-1.

Rasmussen, Henrik H.; Holst, Mette; Kondrup, Jens (2010): Measuring nutritional risk in hospitals. In: *Clinical epidemiology* 2, S. 209–216. DOI: 10.2147/CLEP.S11265.

Reiss, Jens; Iglseder, Bernhard; Kreutzer, Martina; Weilbuchner, Ingrid; Treschnitzer, Wolfgang; Kässmann, Helmut et al. (2016): Case finding for sarcopenia in geriatric inpatients: performance of bioimpedance analysis in comparison to dual X-ray absorptiometry. In: *BMC Geriatr* 16 (1), S. 52. DOI: 10.1186/s12877-016-0228-z.

Rief, Winfried; Shedden-Mora, Meike C.; Laferton, Johannes A. C.; Auer, Charlotte; Petrie, Keith J.; Salzmann, Stefan et al. (2017): Preoperative optimization of patient expectations improves long-term outcome in heart surgery patients: results of the randomized controlled PSY-HEART trial. In: *BMC medicine* 15 (1), S. 4. DOI: 10.1186/s12916-016-0767-3.

Rimington, H.; Weinman, J.; Chambers, J. B. (2010): Predicting outcome after valve replacement. In: *Heart (British Cardiac Society)* 96 (2), S. 118–123. DOI: 10.1136/hrt.2008.160010.

Rockwood, K.; Wolfson, C.; McDowell, I. (2001): The Canadian Study of Health and Aging: organizational lessons from a national, multicenter, epidemiologic study. In: *Int. Psychogeriatr.* 13 Supp 1 (S1), S. 233–237. DOI: 10.1017/s1041610202008177.

Rockwood, Kenneth; Song, Xiaowei; MacKnight, Chris; Bergman, Howard; Hogan, David B.; McDowell, Ian; Mitnitski, Arnold (2005): A global clinical measure of fitness and frailty in elderly people. In: *CMAJ* 173 (5), S. 489–495. DOI: 10.1503/cmaj.050051.

Rubenstein, L. Z.; Harker, J. O.; Salvà, A.; Guigoz, Y.; Vellas, B. (2001): Screening for undernutrition in geriatric practice: developing the short-form mini-nutritional assessment (MNA-SF). In: *The journals of gerontology. Series A, Biological sciences and medical sciences* 56 (6), M366-72. DOI: 10.1093/gerona/56.6.m366.

Sadlonova, Monika; Nagel, Jonas; Becker, Svenja; Neumann, Sophie; Staab, Julia; Meyer, Thomas et al. (2022): Feasibility and preliminary efficacy of perioperative interventions in patients undergoing coronary artery bypass grafting: the randomized controlled I-COPE trial. In: *European journal of cardio-thoracic surgery : official journal of the European Association for Cardio-thoracic Surgery* 62 (1). DOI: 10.1093/ejcts/ezac041.

Salzmann, Stefan; Salzmann-Djufri, Miriam; Wilhelm, Marcel; Euteneuer, Frank (2020): Psychological Preparation for Cardiac Surgery. In: *Current cardiology reports* 22 (12), S. 172. DOI: 10.1007/s11886-020-01424-9.

Sampalis, J.; Boukas, S.; Liberman, M.; Reid, T.; Dupuis, G. (2001): Impact of waiting time on the quality of life of patients awaiting coronary artery bypass grafting. In: *CMAJ* 165 (4), S. 429–433.

Sawatzky, Jo-Ann V.; Kehler, D. Scott; Ready, A. Elizabeth; Lerner, Neal; Boreskie, Sue; Lamont, Darlene et al. (2014): Prehabilitation program for elective coronary artery

bypass graft surgery patients: a pilot randomized controlled study. In: *Clinical rehabilitation* 28 (7), S. 648–657. DOI: 10.1177/0269215513516475.

Schnorpfeil, Pia; Noll, Alexander; Wirtz, Petra; Schulze, Renate; Ehlert, Ulrike; Frey, Karl; Fischer, Joachim E. (2002): Assessment of exhaustion and related risk factors in employees in the manufacturing industry--a cross-sectional study. In: *International archives of occupational and environmental health* 75 (8), S. 535–540. DOI: 10.1007/s00420-002-0369-6.

Scrutinio, Domenico; Giannuzzi, Pantaleo (2008): Comorbidity in patients undergoing coronary artery bypass graft surgery: impact on outcome and implications for cardiac rehabilitation. In: *European journal of cardiovascular prevention and rehabilitation : official journal of the European Society of Cardiology, Working Groups on Epidemiology & Prevention and Cardiac Rehabilitation and Exercise Physiology* 15 (4), S. 379–385. DOI: 10.1097/HJR.0b013e3282fd5c6f.

Seidl, H.; Bowles, D.; Bock, J-O; Brettschneider, C.; Greiner, W.; König, H-H; Holle, R. (2015): FIMA--Fragebogen zur Erhebung von Gesundheitsleistungen im Alter: Entwicklung und Pilotstudie. In: *Gesundheitswesen* 77 (1), S. 46–52. DOI: 10.1055/s-0034-1372618.

Sepehri, Aresh; Beggs, Thomas; Hassan, Ansar; Rigatto, Claudio; Shaw-Daigle, Christine; Tangri, Navdeep; Arora, Rakesh C. (2014): The impact of frailty on outcomes after cardiac surgery: a systematic review. In: *The Journal of thoracic and cardiovascular surgery* 148 (6), S. 3110–3117. DOI: 10.1016/j.jtcvs.2014.07.087.

Singler K, Gosch M, Antwerpen L (2020): Clinical frailty scale. Online verfügbar unter https://www.dggeriatrie.de/images/Bilder/PosterDownload/200331_DGG_Plakat_A4_Clinical_Frailty_Scale_CFS.pdf, zuletzt geprüft am November 2022.

Sipers, Walther M. W. H.; Verdijk, Lex B.; Sipers, Simone J. E.; Schols, Jos M. G. A.; van Loon, Luc J. C. (2016): The Martin Vigorimeter Represents a Reliable and More Practical Tool Than the Jamar Dynamometer to Assess Handgrip Strength in the Geriatric Patient. In: *Journal of the American Medical Directors Association* 17 (5), 466.e1-7. DOI: 10.1016/j.jamda.2016.02.026.

Smedt, Delphine de; Clays, Els; Höfer, Stefan; Oldridge, Neil; Kotseva, Kornelia; Maggioni, Aldo Pietro et al. (2016): Validity and reliability of the HeartQoL questionnaire in a large sample of stable coronary patients: The EUROASPIRE IV Study of the European Society of Cardiology. In: *Eur. J. Prev. Cardiol.* 23 (7), S. 714–721. DOI: 10.1177/2047487315604837.

Smith, Tasha; Gildeh, Nadia; Holmes, Clive (2007): The Montreal Cognitive Assessment: validity and utility in a memory clinic setting. In: *Canadian journal of*

*psychiatry. Revue canadienne de psychiatrie* 52 (5), S. 329–332. DOI: 10.1177/070674370705200508.

Snowdon, David; Haines, Terry P.; Skinner, Elizabeth H. (2014): Preoperative intervention reduces postoperative pulmonary complications but not length of stay in cardiac surgical patients: a systematic review. In: *Journal of Physiotherapy* 60 (2), S. 66–77. DOI: 10.1016/j.jphys.2014.04.002.

Spiroski, Dejan; Andjić, Mojsije; Stojanović, Olivera Ilić; Lazović, Milica; Dikić, Ana Djordjević; Ostojić, Miodrag et al. (2017): Very short/short-term benefit of inpatient/outpatient cardiac rehabilitation programs after coronary artery bypass grafting surgery. In: *Clinical cardiology* 40 (5), S. 281–286. DOI: 10.1002/clc.22656.

Stammers, Andrew N.; Kehler, D. Scott; Afilalo, Jonathan; Avery, Lorraine J.; Bagshaw, Sean M.; Grocott, Hilary P. et al. (2015): Protocol for the PREHAB study-Pre-operative Rehabilitation for reduction of Hospitalization After coronary Bypass and valvular surgery: a randomised controlled trial. In: *BMJ open* 5 (3), e007250. DOI: 10.1136/bmjopen-2014-007250.

Statistisches Bundesamt (2016): 13. koordinierte Bevölkerungsvorausberechnung für Deutschland, Altersaufbau 2050. Online verfügbar unter https://www.destatis.de/bevoelkerungspyramide/#!y=2050.

Steinmetz, Carolin; Bjarnason-Wehrens, Birna; Walther, Thomas; Schaffland, Tim Fabian; Walther, Claudia (2022): Efficacy of prehabilitation prior to cardiac surgery: a systematic review and meta-analysis. In: *American journal of physical medicine & rehabilitation*. DOI: 10.1097/PHM.0000000000002097.

Threapleton, Diane E.; Chung, Roger Y.; Wong, Samuel Y. S.; Wong, Eliza; Chau, Patsy; Woo, Jean et al. (2017): Integrated care for older populations and its implementation facilitators and barriers: A rapid scoping review. In: *International journal for quality in health care : journal of the International Society for Quality in Health Care* 29 (3), S. 327–334. DOI: 10.1093/intqhc/mzx041.

Topp, Robert; Ditmyer, Marcia; King, Karen; Doherty, Kristen; Hornyak, Joseph (2002): The effect of bed rest and potential of prehabilitation on patients in the intensive care unit. In: *AACN clinical issues* 13 (2), S. 263–276. DOI: 10.1097/00044067-200205000-00011.

Vahanian, Alec; Beyersdorf, Friedhelm; Praz, Fabien; Milojevic, Milan; Baldus, Stephan; Bauersachs, Johann et al. (2021): 2021 ESC/EACTS Guidelines for the management of valvular heart disease. In: *European journal of cardio-thoracic surgery : official journal of the European Association for Cardio-thoracic Surgery* 60 (4), S. 727–800. DOI: 10.1093/ejcts/ezab389.

van der Wulp, Kees; van Wely, Marleen; van Heijningen, Lars; van Bakel, Bram; Schoon, Yvonne; Verkroost, Michel et al. (2019): Delirium After Transcatheter Aortic Valve Implantation Under General Anesthesia: Incidence, Predictors, and Relation to Long-Term Survival. In: *Journal of the American Geriatrics Society* 67 (11), S. 2325–2330. DOI: 10.1111/jgs.16087.

Vanhees, Luc; an Stevens; Schepers, Dirk; Defoor, Johan; Rademakers, Frank; Fagard, Robert (2004): Determinants of the effects of physical training and of the complications requiring resuscitation during exercise in patients with cardiovascular disease. In: *European journal of cardiovascular prevention and rehabilitation : official journal of the European Society of Cardiology, Working Groups on Epidemiology & Prevention and Cardiac Rehabilitation and Exercise Physiology* 11 (4), S. 304–312. DOI: 10.1097/01.hjr.0000136458.44614.a2.

Vasunilashorn, Sarinnapha M.; Fong, Tamara G.; Albuquerque, Asha; Marcantonio, Edward R.; Schmitt, Eva M.; Tommet, Douglas et al. (2018): Delirium Severity Post-Surgery and its Relationship with Long-Term Cognitive Decline in a Cohort of Patients without Dementia. In: *Journal of Alzheimer's disease : JAD* 61 (1), S. 347–358. DOI: 10.3233/JAD-170288.

Velazquez, Eric J.; Lee, Kerry L.; Jones, Robert H.; Al-Khalidi, Hussein R.; Hill, James A.; Panza, Julio A. et al. (2016): Coronary-Artery Bypass Surgery in Patients with Ischemic Cardiomyopathy. In: *The New England journal of medicine* 374 (16), S. 1511–1520. DOI: 10.1056/NEJMoa1602001.

Wallace, Meredith; Shelkey, Mary (2007): Katz Index of Independence in Activities of Daily Living (ADL). In: *Urologic nursing* 27 (1), S. 93–94.

Weltärztebund (2013): WMA Deklaration von Helsinki - Ethische Grundsätze für die medizinische Forschung am Menschen. 64. WMA-Generalversammlung im Oktober 2013, Fortaleza (Brasilien). Hg. v. Weltärztebund (WMA). Fortaleza.

Wilson, D.; Cutts, J.; Lees, I.; Mapungwana, S.; Maunganidze, L. (1992): Psychometric properties of the revised UCLA Loneliness Scale and two short-form measures of loneliness in Zimbabwe. In: *Journal of personality assessment* 59 (1), S. 72–81. DOI: 10.1207/s15327752jpa5901_7.

Witlox, Joost; Eurelings, Lisa S. M.; Jonghe, Jos F. M. de; Kalisvaart, Kees J.; Eikelenboom, Piet; van Gool, Willem A. (2010): Delirium in elderly patients and the risk of postdischarge mortality, institutionalization, and dementia: a meta-analysis. In: *JAMA* 304 (4), S. 443–451. DOI: 10.1001/jama.2010.1013.

Yau, Derek King Wai; Underwood, Malcolm John; Joynt, Gavin Matthew; Lee, Anna (2021): Effect of preparative rehabilitation on recovery after cardiac surgery: A

systematic review. In: *Annals of physical and rehabilitation medicine* 64 (2), S. 101391. DOI: 10.1016/j.rehab.2020.03.014.

Zarit, S.; Zarit, J. (1987): Instructions for the burden interview.

Zarit, S. H.; Reever, K. E.; Bach-Peterson, J. (1980): Relatives of the impaired elderly: correlates of feelings of burden. In: *Gerontologist* 20 (6), S. 649–655. DOI: 10.1093/geront/20.6.649.
