# Supplementary material for: Prehabilitation in older patients prior to elective cardiac procedures (PRECOVERY): study protocol of a multicenter randomized controlled trial
Source: Trials. 2023 Aug 15;24:533. doi: 10.1186/s13063-023-07511-w (PMC10426165; doi:10.1186/s13063-023-07511-w)
Supplement: Supplementary file 2 — Additional file file 2. [file 13063_2023_7511_MOESM2_ESM.pdf]

UNIVERSITÄTSMEDIZIN GÖTTINGEN UMG

Statistical Analysis Plan

PREhabilitation “Karl-Heinz” mit Schwerpunkt auf Cardiale und kOgnitiVe Funktionen vor Eingriffen am HERzen: eine AnalYse des Gesundheitzustandes

PRECOVERY

**Study Protocol**	1.1 / 10.01.2023
**EudraCT No.**	-
**Clinicaltrials.gov-No. / DRKS -No.**	-
**Internal Protocol ID No.**	-
**NCT-No.**	-
**SAP Version**	1.0 / 20.01.2023
**SAP Revisions**	-
**Development Phase**	-
**Sponsor**	University Medical Center Göttingen<br>represented by the Head of Clinical trials unit UMG<br>Robert-Koch-Str. 40<br>37075 Göttingen, Germany
**Coordinating Investigator**	Prof. Dr. Christine von Arnim<br>Abteilung für Geriatrie<br>Universitätsmedizin Göttingen (UMG)<br>Robert-Koch-Str. 40<br>37075 Göttingen

Approval of the Statistical Analysis Plan

PRECOVERY

Protocol Version No: V 2 / 20.01.202

Coordinating Investigator<br>Prof. Dr. Christine von Arnim	27.1.2023	
	Date	Signature
Biostatistician<br>Dr. Thomas Asendorf	27.01.2023	
	Date	Signature

Table of Contents

Approval of the Statistical Analysis Plan **2**
Table of Contents **3**
List of Figures **4**
List of Tables **4**
List of Abbreviations **5**
1 Introduction **6**
1.1 Background and Rationale 6
1.2 Objectives and Endpoints 6
1.3 Primary objective and endpoint 8
1.4 Secondary objectives and endpoints 8
1.5 Tertiary/other objectives and endpoints 8
2 Study methods **8**
2.1 Trial design 8
2.2 Randomization 8
2.3 Sample Size 8
2.4 Framework 9
2.5 Statistical Interim Analyses and Stopping Guidance 9
2.6 Timing of the Final Analysis 9
2.7 Timing of Outcome Assessments 9
2.8 Methods against Bias 10
3 Statistical Principles **11**
3.1 Confidence intervals and p-values 11
3.2 Adherence and protocol deviations 11
3.3 Analysis populations 11
4 Trial population **11**
4.1 Screening data 11
4.2 Eligibility 12
4.3 Recruitment 12
4.4 Withdrawal/follow-up 13
4.5 Baseline patient characteristics 13
5 Analysis **13**
5.1 Outcome definitions 13
5.2 Analysis methods 14
5.3 Missing data 15
5.4 Additional analyses 15
5.5 Harms 15
5.6 Statistical software 15
6 References **15**

List of Figures

No table of figures entries found.

List of Tables

Table 1 Objectives and related endpoints 6
Table 2 Timing of Outcome Assessments 9
Table 3 Definition of analysis population 11
Table 4 Baseline patient characteristics .... **Fehler! Textmarke nicht definiert.**

List of Abbreviations

6-MWT *6-Minute Walking Test*

ADL *Activity of daily living*

AE *Adverse Event*

ATS *American Thoracic Society*

BIA *Bioelectrical impedance analysis*

CSHA *Canadian Study of Health and Aging*

DMP *Disease-Management-Program*

HADS *Hospital Anxiety and Depression Scale*

ICU *intensive care unit*

MI *Multiple Imputation*

MNA *Mini Nutritional Assessment*

MoCa *Montreal Cognitive Assessment*

MQ *Maastricht Questionnaire*

PSQI *Pittsburgh Sleep Quality Index*

R-UCLA *Revised UCLA Loneliness Scale*

SAE *Severe Adverse Event*

SAP *Statistical Analysis Plan*

SMI *Self-reported subjective memory impairment*

SPPB *Short Physical Performance Battery*

1 Introduction

This document has been written based on information contained in the trial protocol version 2.0, 01/2023.

The clinical trial aims to investigate the effect of prehabilitating measures in elderly (≥ 75 years) undergoing heart intervention. For this purpose, a randomized, controlled, longitudinal, multicentric, assessor-blinded, two-arm parallel group clinical trial is planned. It is hypothesized, that 12 months after cardiac intervention the intervention group shows

1) improvement of quality of life and one-year survival
2) improvement of everyday function, physical and mental performance
3) reduction of health related costs for the health insurance

in comparison to the control group.

1.1 Background and Rationale

Elderly patients with planned heart intervention undergo a two-week prehabilitation program prior to cardiac intervention. After the program, the heart intervention is conducted and patients are followed up for a total of 12 months.

See the study protocol for more details on the background and rationale of the clinical trial.

1.2 Objectives and Endpoints

To answer the general formulation of the trial a set of primary and secondary endpoints was chosen. Clinical endpoints are evaluated by the trial statistician, while endpoints related to health economic aspects of the trial are evaluated by the Institute for Health Economics UKE and therefore not specified in this SAP. Primary, secondary, tertiary and safety end points are listed as follows.

Table 1 Objectives and related endpoints

	**Objective**	**Endpoint**
**Primary**	Assessment of the health status after 12 months after intervention, to assess the efficacy of the intervention in comparison to standard of care	EQ-5D-5L total score difference at month 12 after intervention, adjusted for baseline
	Assessment of mortality within 12 months after intervention, to assess efficacy of the intervention in comparison to standard of care	Mortality difference within 12 months after intervention
**Secondary**	Evaluation of the functional status of older adults is through assessment of the ability to perform activities of daily living (ADL)	Katz-Index (ADL) difference at month 12 after intervention, adjusted for baseline
	Assessment of age-related mobility disability and physical functioning	Short Physical Performance Battery difference at month 12 after

	Objective	Endpoint
		intervention, adjusted for baseline (SPPB)
	Assessment of cognitive impairment	Montreal Cognitive Assessment (MoCa) difference at month 12 after intervention, adjusted for baseline
	Assessment of heart related quality of life	HeartQoL difference at month 12 after intervention, adjusted for baseline
	Determination of perceived anxiety and depression	Hospital Anxiety and Depression Scale (HADS) difference at month 12 after intervention, adjusted for baseline
	Subjective reporting of overall health	EQ-VAS difference at month 12 after intervention, adjusted for baseline
**Tertiary**	30-day mortality to assess short-term effects of the intervention	Mortality difference within 30 days after intervention
	Evaluation of everyday physical capabilities	Grip Strength difference at month 12 after intervention, adjusted for baseline
	Evaluation of everyday physical capabilities (aerobic capacity and endurance)	6-Minute Walking Test (6-MWT) difference at month 12 after intervention, adjusted for baseline
	Evaluate malnourishment or risk of malnutrition	Mini Nutritional Assessment (MNA) difference at month 12 after intervention, adjusted for baseline
	Evaluate influence of intervention on sleep quality	Pittsburgh Sleep Quality Index (PSQI) difference at month 12 after intervention, adjusted for baseline
	Measure vitality exhaustion as a prodromal syndrome of heart attack	Maastricht Questionnaire (MQ) difference at month 12 after intervention, adjusted for baseline
	Measure self-reported loneliness of patients	Revised UCLA Loneliness Scale (R-UCLA) difference at month 12 after intervention, adjusted for baseline
	Evaluation of everyday cognitive capabilities	Self-reported subjective memory impairment (SMI) difference at month 12 after intervention, adjusted for baseline
	Assessment of age-related changes in frailty	Canadian Study of Health and Aging (CSHA) Frailty-Index difference at month 12 after intervention, adjusted for baseline
	Assessment of dyspnoe	American Thoracic Society scale (ATS scale) difference at month 12 after intervention, adjusted for baseline
**Safety**	Assessment of safety of the intervention	Number of Adverse Events (AEs) per group
		Number of Severe Adverse Events (SAEs) per group

	Objective	Endpoint
		Rate of admissions to intensive care unit (ICU)
		Number of pre- and post interventional complications per group

1.3 Primary objective and endpoint

To determine health status the EuroQoL (EQ-5D-5L) questionnaire is used. It is a validated instrument for measuring health status in clinical trials, observational studies and health economic trials and valid for patients with coronary heart disease.

1.4 Secondary objectives and endpoints

Secondary endpoints are recorded as listed in Table 1. For further details, see Section 5.1.

1.5 Tertiary/other objectives and endpoints

Tertiary endpoints are recorded as listed in Table 1.

2 Study methods

2.1 Trial design

This study is a two-arm randomised controlled longitudinal multicentre clinical trial. Patients are screened and randomised either to the intervention or standard of care group and followed-up 12 months after their planned intervention.

2.2 Randomization

Patients are randomized in a 1:1 ratio to either intervention or control (standard of care). Block-randomization is performed with random block length, stratified for center, age (<81 years vs. ≥81 years) and sex.

2.3 Sample Size

The sample size estimate is based on the minimally important difference calculated in McClure et al. 2017 (McClure, Al Sayah, Xie, Luo, & Johnson, 2017), and the observed standard deviation of the EQ-5D-5L reported in McClure et al. 2018 (McClure, Al Sayah, Ohinmaa, & Johnson, 2018).

Assuming a total sample size of 338 equally split into two groups (1:1 randomization), the analysis of covariance would have 80% power to detect a two-sided significance level of 5%, assuming a mean difference of 0.045 between groups at end of study and an R^2 of 0.25 with included covariates. Further assuming a dropout-rate of 20%, a sample size of 422 patients (211 per group) was chosen to adequately power the trial. Power calculations were performed in nQuery 8.

2.4 Framework

Unless stated otherwise, all endpoints are tested for superiority of the intervention over control.

2.5 Statistical Interim Analyses and Stopping Guidance

No interim analyses are planned.

Non-comparative assessments of data quality is performed on a regular basis, following the data management plan. A blinded review is planned when 50% of all patients have been recruited to the trial. The blinded review will include a non-comparative re-estimation of underlying nuisance parameters of the sample size, the dropout rate and mortality. A report of the results will be presented to- and discussed with the coordinating investigator.

A second blinded review is planned after the data has been completely entered. The second blinded review will focus on data completeness and quality to ensure consistency in data received.

In case of expected mortality rates higher than 20% at end of study, the shared frailty model is considered to reduce bias of estimates when integrating non-ignorable missingness.

2.6 Timing of the Final Analysis

The final analysis will take place when all outcomes have been collected, a blinded review of the data was performed and the database is locked.

2.7 Timing of Outcome Assessments

Several fixed time points for outcome assessment are intended. Following table displays the assessment of variables within these time points:

Table 2 Timing of Outcome Assessments

	Baseline/ Randomisation (Day -21)	Pre-OP (Day -1)	Post-OP (Day 0)	Day 30	Month 6	Month 12
EQ-5D-5L	X	X	X	X	X	X
Mortality	Continuously Measured					
ADL	X	X	X	X	X	X
SPPB	X	X				X
MoCa	X	X				X
HeartQoL	X	X		X	X	X
HADS	X	X		X	X	X

Grip Strength	X	X				X
6-MWT	X	X				X
BIA	X	X				X
MNA	X	X				X
PSQI	X	X				X
MQ	X	X		X		X
R-UCLA	X			X		X
SMI	X	X		X	X	X
CSHA Frailty-Index	X	X	X			X
ATS scale	X	X	X	X	X	X
Safety Endpoints	Continuously Measured					

Deviations from the ideal time point of assessment, relative to the date of intervention, are allowed to a certain degree as follows:

- Baseline/Randomisation: day -35 to day -12; but always before pre-habilitation program
- Pre-OP: day -10 to day -1; but always after end of pre-habilitation program and before OP
- Post-OP: day 0 to day 7; but always after OP
- Day 30: day 14 to day 44
- Month 6 ± 30 days
- Month 12 ± 60 days

Deviation higher than the intended are marked as protocol deviations and will be excluded from the primary analysis.

2.8 Methods against Bias

Selection Bias: Patients are randomized in a 1:1 ratio to either intervention or control (standard of care). Block-randomization is performed with random block length, stratified for center, age (<81 years vs. ≥81 years) and sex. Performance Bias: Blinding of Patients is not possible. Study personnel not involved in the pre-habilitation are blinded. Detection Bias: Outcomes are assessed by blinded study personnel. Attrition Bias: Missing values due to death are taken into account through a joint frailty model for longitudinal data. Further missing values are treated using multiple imputation methods. Sensitivity analyses using complete cases are planned to discuss possible differences. Reporting Bias: Confirmatory analyses are pre-specified within this SAP. All results will be published independently of whether or not statistical significance could be shown.

3 Statistical Principles

3.1 Confidence intervals and p-values

Unless specified otherwise, all tests will be performed two-sided with 5% significance level. Consequently, confidence intervals will be reported with 95% confidence level.

3.2 Adherence and protocol deviations

Participants will be considered adherent to the intervention if they complete at least 50% of the pre-habilitation measures.

3.3 Analysis populations

The choice of analysis population and estimands is based on the ICH E9 (R1) addendum on estimands and sensitivity analysis in clinical trials to the guideline on statistical principles for clinical trials from the 17th of February 2020.

Table 3 Definition of analysis population

Full Analysis Set	All patients who were enrolled into the study, independent of whether or not protocol requirements were met
Intention to Treat Set	All patients who were randomized to either intervention or control
Per Protocol Set	All patients who followed the protocol without major protocol deviations
Safety Population	All patients who took part in the pre-habilitation program and/or underwent cardiac intervention

Primary analysis is planned following the treatment policy strategy with intention-to-treat principle. In case of 1-year mortality rates higher than 20%, a composite variable strategy to incorporate non-informative missingness from death will be employed instead. Sensitivity analysis are conducted for the per protocol set. Safety endpoints are collected through the safety population.

4 Trial population

4.1 Screening data

Screening data will be reported and described within a CONSORT flowchart.

4.2 Eligibility

Inclusion criteria for patients:

1. Patients who are 75 years old or older will be included if one of the following surgeries are necessary:
   a) Replacement of heart valve with prosthesis (5-351)
   b) Change of prosthetic heart valves (5-352)
   c) Valvuloplasty (5-353)
   d) Other heart valve surgeries (5-354)
   e) Minimally invasive operations on heart valves (incl. TAVI & MitraClip) (5-35a)
   f) Desobliteration (endarterectomy) of the coronary arteries (5-360)
   g) Placement of an aortocoronary bypass (5-361)
   h) Placement of an aortocoronary bypass by minimally invasive technique (5-362)
   i) Other revascularization of the heart (5-363)
2. Sufficient degree of independence
3. Health insured (AOK Lower Saxony)
4. Capacity for consent
5. Willingness to take part in the study voluntarily after being informed with a signed declaration of consent
6. Sufficient knowledge of the German language

Exclusion criteria for patients:

1. Inability to give consent
2. Katz-Index of 0
3. Need for treatment in an acute hospital setting
4. severe dementia; severe mental disorders (acute psychoses, severe depressive episodes, acute suicidality), acute delirium
5. Diagnosis of acute alcohol or drug abuse
6. Unstable angina pectoris

4.3 Recruitment

Recruitment numbers will be reported and described within a CONSORT flowchart.

4.4 Withdrawal/follow-up

A CONSORT flowchart is presented to document reasons for withdrawal. Reasons for drop-out are collected in a corresponding form within the trial data base. Other protocol deviations are assessed from individual protocols and also reported within the CONSORT flowchart.

4.5 Baseline patient characteristics

All baseline patient characteristics will be summarized using descriptive statistics (e.g., mean, standard deviation, median, interquartile range) and appropriate graphical methods (e.g., boxplots, barplots, histograms, progress plots) depending on the data type. Due to randomization, it is assumed that data are equally distributed in both treatment groups. Therefore, no statistical comparison of data is intended.

5 Analysis

5.1 Outcome definitions

A full list of outcomes and their timing is described in Section 2.7. For a detailed description of all outcomes see study protocol section 5.

EQ-5D-5L (Quality of life) is a questionnaire designed to assess the quality of life of patients in 5 different dimensions using 5 questions assigned to 5 domains: mobility, self-care, usual activities, pain/discomfort, and anxiety/depression. The responses are on a 5-point-likert scale ranging from 1 (indicating no problem) to 5 (indicating unable to/extreme problems). Additionally, the **EQ-VAS** is also recorded, which asks for the general health of a patient on a scale from 0 to 100. For the analysis of quality of life, the EQ-5D-5L utility score and EQ-VAS will be used. To obtain the index values of the EQ-5D-5L for each participant, the EQ-5D-5L Crosswalk Index Value Calculator tool for German index value sets will be used (Van Hout, et al., 2012). MI method will be utilised in imputing missing 5Q-5D-5L utility scores and EQ-VAS.

Katz-Index (ADL) is an instrument to assess functional status as a measurement of the client's ability to perform activities of daily living independently. The Index ranks adequacy of performance in the six functions of bathing, dressing, toileting, transferring, continence, and feeding. Clients are scored yes/no for independence in each of the six functions. A score of 6 indicates full function, 4 indicates moderate impairment, and 2 or less indicates severe functional impairment (Wallace & Shelkey, 2007).

SPPB is a group of measures that combines the results of the gait speed, chair stand and balance tests. (Guralnik, et al., 1994). In each task, the patient can score up to four points. At the end, the results of all three tests are added together. The maximum possible total score is therefore twelve, the minimum zero points (Büsching, 2015).

MoCa is a 1-page 30-point test. Details on the specific items are as follows. The short-term memory recall task (5 points) involves 2 learning trials of 5 nouns and delayed recall after approximately 5 minutes. Visuospatial abilities are assessed using a clock-drawing task (3 points) and a 3D cube copy (1 point). Multiple aspects of executive functions are assessed using an alternation task adapted from the Trail Making B task (1 point), a phonemic fluency task (1 point), and a 2-item verbal abstraction task (2 points). Attention, concentration, and working memory are evaluated using a sustained attention task (1 point), a serial subtraction task (3 points), and digits forward and backward (1 point each).

Language is assessed using a 3-item confrontation naming task with low-familiarity animals (3 points), repetition of 2 syntactically complex sentences (2 points), and the aforementioned fluency task. Orientation to time and place is evaluated (6 points) (Nasreddine, et al., 2005).

HeartQoL consists of a physical (10 items) and an emotional (4 items) subscale making up the 14-item global scale with higher values representing better HRQL. All items on the physical (e.g., “In the last 4 weeks, have you been bothered by having to lift or move heavy objects?”) and the emotional subscale (e.g., “In the last 4 weeks, have you been bothered by being worried?”) are answered on a 4-point scale ranging from “bothered a lot” (= 0) to “not bothered” (= 3) (Oldridge, et al., 2014).

HADS is a 14-item self-assessment questionnaire used to screen for anxiety and depressive symptoms with an anxiety (e.g., “I get a sort of frightened feeling as if something awful is about to happen”) and a depression subscale (e.g., “I look forward with enjoyment to things”). The items are answered on a scale ranging from 0 to 3 with higher scores representing higher levels of anxiety or depression (Petermann, 2015).

Grip strength, 6-MWT and **BIA** are measured on continuous scales.

MNA provides a single, rapid assessment of nutritional status in elderly patients in outpatient clinics, hospitals, and nursing homes. It has 6 items and scores values between 0 and 14.

PSQI is a self-rated. questionnaire which assesses sleep quality and disturbances over a 1-month time interval. The measure consists of 19 individual items, creating 7 components that produce one global score. Each component is weighted on a 0–3 interval scale. The global PSQI score is then calculated by summing the seven component scores, providing an overall score ranging from 0 to 21, where lower scores denote a healthier sleep quality.

R-UCLA is described and used as a unidimensional measure of loneliness; conceptualizing and assessing loneliness as a unitary, global experience. The score contains 20 items on a likert scale from 1 to 4. The total score (adding up all items) therefore ranges from 20 to 80.

CSHA is a frailty scale ranging from 1 (very fit) to 9 (terminally ill).

5.2 Analysis methods

The primary endpoint is analysed using a joint frailty model for longitudinal data and terminal event (Rondeau, Mazroui, & Gonzalez, 2021) (Król, et al., 2016). Such a joint-model allows to take into account the missing values due to expected mortality, which are non-ignorable in the evaluation of quality of life. Stratifying factors age, sex, disease-management-program (DMP) participation, and center are included as covariates in the model. Both the impact of treatment on longitudinally assessed EQ-5D-5L (t_1 to t_6), and on 1-year survival (co-primary endpoint) are represented via the model. Regression coefficients of the statistical model, specifically the interaction between intervention and time, and the hazard ratio of intervention, are reported with standard errors, asymptotic 95% confidence intervals and p-values (two-sided test versus null hypothesis of no group difference). Time courses of EQ-5D-5L are presented group-specifically longitudinally and survival rates descriptively using Kaplan-Meier curves.

The secondary endpoint HEART-QOL is evaluated analogously to the primary endpoint. Secondary endpoints ADL, SPPB, MoCa, and HADS are evaluated using mixed linear models with the factors time, treatment and the interaction as well as the covariates age, gender, participation in the DMP and center. Multiple imputation (van Buuren & Groothuis-Oudshoorn, 2011) is planned for the intention-to-treat evaluation. For inter- and intra-group comparisons,

marginal means (Lenth, 2023) will be calculated and treatment effects reported with 95% confidence intervals. Additional endpoints (see Table 1) will also be evaluated using generalized mixed linear models, depending on data type. The 30-day mortality is analysed using a Cox regression with the additional factors of sex, age, disease management program and center.

5.3 Missing data

For participants to be included, they must have baseline values and at least one follow-up value, then the missing data will be imputed using multiple imputation (MI). Details on the type of multiple imputation will be added following a blinded data review.

5.4 Additional analyses

Additional analyses, especially those of tertiary endpoints, will be on an explorative basis.

5.5 Harms

AEs and SAEs as well as pre- and post interventional complications will be summarized as frequencies and percentages per group and the rate of admissions to intensive care unit per group will be calculated.

5.6 Statistical software

All analyses will be carried out in the current version of R or SAS. Employed packages and the specific version are documented within the statistical report. The primary endpoint is analysed using *frailtypack* from R in its current version. Secondary endpoints will be analysed using packages *lme4* and *emmeans*. Multiple imputation is conducted using the package *mice*.

6 References

Büsching, G. (2015). Short Physical Performance Battery Test–Ein Muss in der Geriatrie. *physiopraxis, 13*(01), S. 42-43.

Guralnik, J. M., Simonsick, E. M., Ferrucci, L., Glynn, R. J., Berkman, L. F., Blazer, D. G., & Wallace, R. B. (1994). A short physical performance battery assessing lower extremity function: association with self-reported disability and prediction of mortality and nursing home admission. *Journal of gerontology, 49*(2), S. M85-M94.

Henderson, R., Diggle, P., & Dobson, A. (2000). Joint modelling of longitudinal measurements and event time data. *Biostatistics, 1*(4), S. 465-480.

Król, A., Ferrer, L., Pignon, J.-P., Proust-Lima, C., Ducreux, M., & Bouché, O. (2016). Joint model for left-censored longitudinal data, recurrent. *Biometrics, 72*(3), S. 907–916.

Lenth, R. (2023). emmeans: Estimated Marginal Means, aka Least-Squares Means. Von https://CRAN.R-project.org/package=emmeans abgerufen

McClure, N. S., Al Sayah, F., Ohinmaa, A., & Johnson, J. A. (2018). Minimally important difference of the EQ-5D-5L index score in adults with type 2 diabetes. *Value in Health, 21*(9), S. 1090-1097.

McClure, N. S., Al Sayah, F., Xie, F., Luo, N., & Johnson, J. A. (2017). Instrument-defined estimates of the minimally important difference for EQ-5D-5L index scores. *Value in Health, 20*(4), S. 644-650.

Nasreddine, Z. S., Phillips, N. A., Bédirian, V., Charbonneau, S., Whitehead, V., Collin, I., & Chertkow, H. (2005). The Montreal Cognitive Assessment, MoCA: a brief screening tool for mild cognitive impairment. *Journal of the American Geriatrics Society, 53*(4), S. 695-699.

Oldridge, N., Höfer, S., McGee, H., Conroy, R., Doyle, F., Saner, H., & Investigators, H. P. (2014). The HeartQoL: Part I. Development of a new core health-related quality of life questionnaire for patients with ischemic heart disease. *European journal of preventive cardiology, 21*(1), S. 90-97.

Petermann, F. (2015). Hospital anxiety and depression scale, deutsche version (HADS-D). *Zeitschrift für Psychiatrie, Psychologie und Psychotherapie*.

Rondeau, V., Mazroui, Y., & Gonzalez, J. R. (2021). frailtypack: An R Package for the Analysis of Correlated Survival Data with Frailty Models Using Penalized Likelihood Estimation or Parametrical Estimation. *Journal of Statistical Software, 47*(4), S. 1-28. Von https://www.jstatsoft.org/v47/i04/ abgerufen

van Buuren, S., & Groothuis-Oudshoorn, K. (2011). mice: Multivariate Imputation by Chained Equations in R. *ournal of Statistical Software, 45*(3), S. 1-67. doi:10.18637/jss.v045.i03

Van Hout, B., F, J. M., Feng, Y.-S., Kohlmann, T., Busschbach, J., Golicki, D., . . . Pickard, A. S. (2012). Interim scoring for the EQ-5D-5L: Mapping the EQ-5D-5L to EQ-5D-3L value sets. *Value in health, 15*(5), 708-715.

Wallace, M., & Shelkey, M. (2007). Katz index of independence in activities of daily living (ADL). *Urol Nurs*(27(1)), S. 93-94.
