# Supplementary material for: Prehabilitation in older patients prior to elective cardiac procedures (PRECOVERY): study protocol of a multicenter randomized controlled trial
Source: Trials. 2023 Aug 15;24:533. doi: 10.1186/s13063-023-07511-w (PMC10426165; doi:10.1186/s13063-023-07511-w)
Supplement: Supplementary file 3 — Additional file file 3. [file 13063_2023_7511_MOESM3_ESM.pdf]

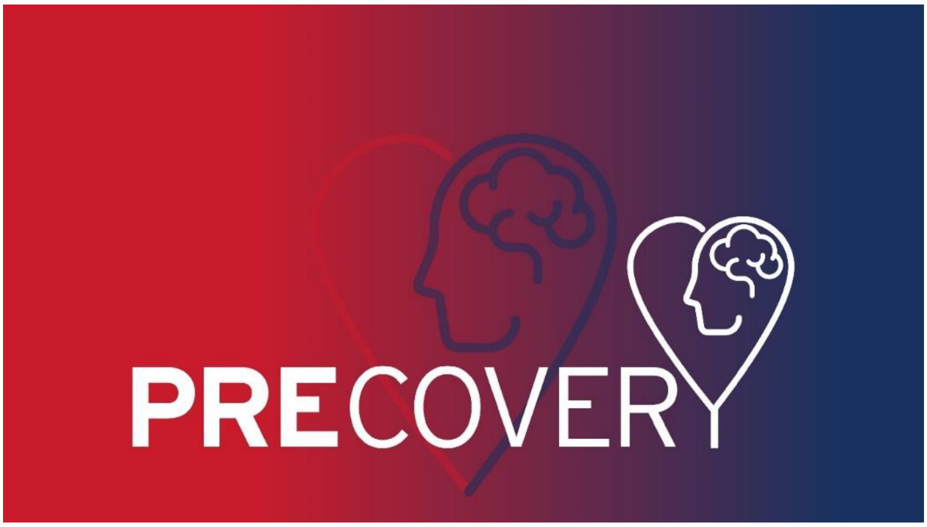

Treatment manual of Prehabilitation intervention “Karl-Heinz”

Basic medical-therapeutic concept

Version 2.0 from 11th April 2023

Created by:

Prof. Dr. med. Christine von Arnim
Specialist in Neurology - Geriatrics - Rehabilitation - Social Medicine
Director of the Geriatric Clinic
Robert-Koch-Str. 40
37075 Goettingen

Dr. med. Monika Sadlonova
Specialist in Psychosomatic Medicine and Psychotherapy
Department of Psychosomatic Medicine and Psychotherapy in Scientific Liaison with Department of Cardiac, Thoracic and Vascular Surgery
Robert-Koch-Str. 40
37075 Goettingen

Dr. Sportwiss. Carolin Steinmetz
Research assistant
Clinic for Geriatrics
Robert-Koch-Str. 40
37075 Goettingen

Jun.-Prof. Dr. Sportwiss. Thomas Schmidt
Institute for Circulation Research and Sports Medicine
German Sports University Cologne
At the Sportpark Müngersdorf 6
50933 Cologne

M.Sc. Katja Roth
Institute for Circulation Research and Sports Medicine
German Sports University Cologne
At the Sportpark Müngersdorf 6
50933 Cologne

Dr. rer. medic. /Dipl.-Psychologist Simone Krieger
Research assistant
Department of Psychosomatic Medicine and Psychotherapy
By-Siebold-Str. 3
37075 Goettingen

Dr. Stephanie Heinemann
Researcher
Clinic for Geriatrics
Robert-Koch-Str. 40
37075 Goettingen

M.Sc. Anne Linke
Rehabilitation psychologist
Clinic for Geriatrics
Robert-Koch-Str. 40
37075 Goettingen

M.Sc. Jessica Peters
Nutritionist
Clinic for Geriatrics
Robert-Koch-Str. 40
37075 Goettingen

Table of content

LIST OF TABLES 4

1 INTRODUCTION 5

1.1 Indication for cardiac prehabilitation 5

2 PREHABILITATION MEASURE BEFORE THE ELECTIVE PROCEDURE 5

2.1 General prehabilitation goals 5

2.2 Modules of Prehabilitation 6

Module 1: Sports and exercise therapy 6

Module 2: Occupational Therapy 7

Module 3: Cognitive Training 8

Module 4: Psychosocial support 9

Module 5: Disease specific education 10

Module 6: Informative talks with relatives 11

Module 7: Special hygiene training/nutritional intervention 12

3 THERAPY UNITS, WEEKLY SCHEDULE & MINIMUM REQUIREMENTS 13

List of tables

Table 1. OPS codes for patients indicated for of prehabilitation. 5

Table 2. Therapy units with duration and frequency per week 13

Table 3. Exemplary weekly schedule of prehabilitation 14

Table 4. Overview of the minimum of therapy requirements during prehabilitation program “Karl-Heinz” 15

1 Introduction

1.1 Indication for cardiac prehabilitation

The prehabilitation program “Karl-Heinz” includes patients aged ≥ 75 years of age with a basic cardiac disease prior to major planned interventional or surgical procedure.

Table 1. OPS codes for patients indicated for of prehabilitation.

OPS Code	Procedure
5-35	Surgery on cardiac valves and septa as well as vessels near the heart
5-351	Replacement of heart valves with prosthesis
5-352	Change of heart valve prostheses
5-353	Valvuloplasty
5-354	Other surgeries on heart valves
5-35a	Minimally invasive surgery on heart valves
5-36	Surgery on coronary vessels
5-360	Desobliteration (endarterectomy) of coronary arteries
5-361	Creating an aortoronary bypass
5-362	Creating an aortocoronary bypass using minimally invasive technique
5-363	Other revascularisation of the heart

Abbreviation. OPS = operation and procedure codes.

2 Prehabilitation measure before the elective procedure

2.1 General prehabilitation goals

Prehabilitation includes a multimodal, interdisciplinary and individually adapted therapeutic approach to achieve the best possible physical and mental health before a planned procedure in a cardiac rehabilitation center. By strengthening the physical exercise capacity as well as the continuous implementation of everyday activities prior to cardiac procedures, we expect an effective prevention of postoperative/post-interventional complications, premature frailty, and loss of independence. An interactive treatment concept consisting of medical, cognitive behavioral and psychosocial interventions can lead to the reduction of fear of movement, a realistic assessment of individual capacity, improvement of body perception, optimization of cardiovascular risk factors as well as a corresponding reduction of anxiety and depression. By advising relatives, an intensification of social support and long-term treatment motivation as well as adherence can be achieved. From a defined pool of therapy modules, the treating physicians of the rehabilitation centers compile the respective treatment plan individually.

General objectives of the prehabilitation intervention:

1) Improvement of subjective health by multidisciplinary prehabilitation one year after an elective cardiac procedure (e.g., transcatheter aortic valve replacement, open surgical or minimal surgical aortic valve replacement).
2) Improvement of everyday function, frailty, depression and anxiety, physical and cognitive performance, as well as a reduction in the number of postoperative/post-interventional complications and delirium prevalence.

The following professional groups are available in the prehabilitation centers to implement the “Karl-Heinz” treatment manual: physicians, nurses, physiotherapists, sports scientists/ therapists, occupational therapists, massage therapist, psychologists, art therapists, nutrition therapists, and clinical social workers.

Under 2.2, the specific modules of prehabilitation and their minimum requirements are explained, which are offered in the above disciplines and thus guarantee a standard for the care of the patients. Group therapies can be offered together with patients inside cardiac rehabilitation in order to ensure practical implementation and to promote the exchange between rehabilitation and prehabilitation patients.

2.2 Modules of Prehabilitation

Module 1: Sports and exercise therapy

The module “Sport and Exercise Therapy” includes the training forms of aerobic endurance training, dynamic strength training, flexibility and coordination training as well as breathing therapy. The contents of the individual training interventions are based on current study results, surveys of the participating rehabilitation centers as well as expert interviews. Due to the low scientific evidence and the high heterogeneity of the existing prehabilitation training intervention studies in cardiac patients, the training forms were designed in addition to existing secondary preventive recommendations for cardiological sports and exercise therapy.

Concrete strategies:

- Aerobic endurance training: minimum of 45-90 minutes per week, at least 3x per week, continuous method, 40-60% $Watt_{max}$, 65-75% HF_{max}, RPE-Scale: 11-14)
- Dynamic strength training + flexibility training (focus of the exercises: muscle groups prone to shortening): two times per week for at least 60, muscular strength endurance training, 10-15 repetitions, 1 to 3 sets, 30-50% 1RM, RPE-Scale: 12-13)
- Coordination training (focus on exercises to improve the balance ability): at least 90 min per week, 3x per week)
- Breathing therapy (focus on breast, abdominal, flank breathing and lip brake): at least 90 min per week, 3 x per week in a group setting, supplemental daily self-training with the help of a breathing volume trainer)

All training forms are carried out by a qualified professional, such as a physiotherapist, sports scientist/therapist. An ECG monitoring while exercising is recommended.

Precautions:

Care must be taken to perform exercises in a manner that does not cause symptoms such as dizziness or malaise. Patients with presyncope, syncope or dizziness are advised against any type of exercise.

The content of this module is mainly based on the following references:

American College of Sports M, Chodzko-Zajko WJ, Proctor DN, Fiatarone Singh MA, Minson CT, Nigg CR, et al. American College of Sports Medicine position stand. Exercise and physical activity for older adults. Med Sci Sports Exerc. 2009;41:1510–30. https:// doi.org/10.1249/MSS.0b013e3181a0c95c.; Nelson ME, Rejeski WJ, Blair SN, Duncan PW, Judge JO, King AC, et al. Physical activity and public health in older adults: recommendation from the American College of Sports Medicine and the American Heart Association. Med Sci Sports Exerc. 2007;39:1435–45. https://doi.org/10.1249/mss.0b013e3180616aa2.; Arthur

HM, Daniels C, McKelvie R, Hirsh J, Rush B: Effect of a preoperative intervention on preoperative and postoperative outcomes in low-risk patients awaiting elective coronary artery bypass graft surgery. A randomized, controlled trial. Ann Intern Med 2000;133:253–62.; Albrecht, K. & Meyer, S. (2014, 22. Oktober). Stretching und Beweglichkeit: Das neue Expertenhandbuch (3., überarbeitete). Karl F. Haug.; Bjarnason-Wehrens B et al. Leitlinie körperliche Aktivität zur Sekundärprävention und Therapie kardiovaskulärer Erkrankungen. Clin Res Cardiol Suppl 2009;4(3):19; Froböse, I & Wilke, C. (2015). Training in der Therapie. Grundlagen. (4. Aufl.). Urban & Fischer: München; Hulzebos, E. H. J., Helders, P. J. M., Favié, N. J., De Bie, R. A., Brutel de la Riviere, A. & Van Meeteren, N. L. U. (2006, 18. Oktober). Preoperative Intensive Inspiratory Muscle Training to Prevent Postoperative Pulmonary Complications in High-Risk Patients Undergoing CABG Surgery. JAMA, 296(15), 1851. https://doi.org/10.1001/jama.296.15.1851; AWMF online, DGPR, SCPRS und ÖKG. S3 – Leitlinie zur kardiologischen Rehabilitation (LL-KardReha) im deutschsprachigen Raum Europas, Deutschland, Österreich, Schweiz (D-A-CH), Langversion – Teil 1. 2020. https://register.awmf.org/assets/guidelines/133-001k_S3-Kardiologische-Rehabilitation-in-D-A-CH_2020-12_-_verlaengert.pdf.; Schwan U NR. Training mit Herzpatienten. 2021.; Stammers AN: Optimizing the pre-operative risk profile of older adults undergoing elective cardiac surgery: A randomized controlled trial [Master's thesis]. Manitoba, Canada: University of Manitoba, 2016., Steinmetz C, Bjarnason-Wehrens B, Baumgarten H, Walther T, Mengden T, Walther C: Prehabilitation in patients awaiting elective coronary artery bypass graft surgery – effects on functional capacity and quality of life: a randomized controlled trial. Clin Rehabil 2020 Oct; 34(10):1256–67. Thomschke, R. (2017, 27. November). Beweglichkeits-Training: Übungen für mehr Flexibilität und zur Muskelentspannung (Trainingsreihe von Ronald Thomschke) (4.). Steffen Verlag.,

Module 2: Occupational Therapy

The focus and overall goal of occupational therapy is the preservation and promotion of the greatest possible independence. This includes, among other things, the promotion of resilience and endurance, training to change everyday routines, adaptation of the daily structure, and the use of limited resources. In order to increase compliance and adherence, an addressee-oriented basic attitude is effective.

Activities of Daily Living (ADL)

Concrete strategies:

- 45 minutes each session, 1-2x per week
- Focus on independence in eating, drinking, personal hygiene and clothing, improvement of gross and fine motor skills if necessary – emphasis on the postoperative phase (methods: therapy oriented to everyday life, treatment according to the Bobath concept)

Precautions:

In conversations with the patients we advise them to be prepared for the fact that their abilities in the area of activities of daily living will be limited after surgery and will first need to be rebuilt. Likewise, we point out that their recovery may occur less quickly than expected. A possible pressure of expectation and performance can be taken so that there is no motivational crack. In order to increase compliance and adherence, it is advisable to ask in advance what is particularly important and desirable for patients in this area.

Brain performance training in occupational therapy

Concrete strategies:

- 45 minutes each session, 1-2x per week
- Focus on promotion and training of cognition, development of everyday cognitive performance (methods: daily oriented therapy, treatment with Coggack® or RehaCom®)

The content of this module is mainly based on the following references:

Habermann, C. und Kolster, F. (2020). Ergotherapie im Arbeitsfeld Neurologie. New York, Leipzig: Thieme Verlag; Habermann, Wittmershaus (Hg.) (2005): Ergotherapie im Arbeitsfeld der Geriatrie. 1. Aufl. Stuttgart: Thieme.

Module 3: Cognitive Training

Cognitive skills include all processes of thought and perception, including: attention, executive functions (e.g., problem solving, planning, action, thought processes), perception processes (e.g., visual-spatial) and memory with its memory functions themselves (e.g., working memory, long-term and short-term memory). Also, movements are already simple cognitive processes, controlled by memory. By specifically improving the cognitive performance of geriatric patients, delirium prevention is implemented before an elective procedure. An individual diagnosis of cognitive performance at the beginning of prehabilitation is important.

Everyday memory strategies

<u>Concrete strategies</u>:

- 10-15 minutes each session, 1-3 times per week
- Focus on improving attention and short-term memory, associative learning to strengthen the verbal fluency in relation to everyday tasks (methods: discuss day-to-day tasks; train sequences; classify processes in the day-to-day events; perform small exercise sequences)

<u>Precautions</u>:

We recommend making sure that patients are not cognitively overwhelmed, discouraged or frustrated. Therefore, please carefully observe the time window of the concentration range so that patients are required and encouraged.

Attention Training

<u>Concrete strategies</u>:

- 20-30 minutes each session, 2-5 times per week
- Focus on improving concentration, attention and short-term memory, associative learning and strengthening verbal fluency (methods: concentration and memory exercises in the group, memory aid for remembering texts, training of visual attention e.g. on the computer).

<u>Precautions</u>:

Again, it is important make sure that patients are not cognitively overwhelmed. Attention and concentration spans can be individually adjusted and then serve as motivation.

Orientation strategies

<u>Concrete strategies</u>:

- 5-10 minutes twice per day, each day

- Focus on preservation and/or improvement of orientation (methods: Routinely trigger orientation/self-orientation)

Precautions:

Best possible circumstances are to keep the same room for patients or to refrain from many room changes. Please keep the number of identifiers moderate (‘as much as necessary – as few as possible’).

Mediation of procedure and daily routine prior to cardiac intervention/surgery

Concrete strategies:

- 15-30 minutes, one conversation prior to cardiac procedure
- Focus on providing a structured aid specifically for the day of the procedure. Visualize and make available the daily overview together with the patients (methods: concentration and memory exercises in the group, memory aids for reminding texts, training visual attention, e.g. on the computer)

Precautions:

Here, it is advisable to present patients with only a few appointments to avoid unnecessary confusion. It is important to find out which self-service is offered and where hospital staff support is available to help patients during their stay. Patients can quickly feel overwhelmed: Especially (too) much information can cause anxiety and trigger unnecessary stress reactions.

The content of this module is mainly based on the following references:

Finauer, G. (2019). Therapiemanuale für die neurobiologische Rehabilitation. Heidelberg, Berlin: Springer; Goldenberg, G. (2017). Neuropsychologie. Grundlagen, Klinik, Rehabilitation. München: Elsevier; Habermann, Wittmershaus (Hg.) (2005): Ergotherapie im Arbeitsfeld der Geriatrie. 1. Aufl. Stuttgart: Thieme.

Module 4: Psychosocial support

Psychological interventions are an important part of multimodal cardiological rehabilitation in German-speaking countries. The therapeutic objectives of the psychological sessions before a planned surgical/interventional procedure can be divided as follows:

1. Improvement of affective comorbidity (e.g., reduction of anxiety and depression)
2. Strengthening self-management (e.g., strengthening therapy motivation, development of adequate/positive health beliefs, assumption of responsibility)
3. Improvement of postoperative/post-interventional quality of life (e.g., optimization of disease management with resource activation that gains confidence in the functioning of one’s own body)

These interventions serve as preparation for cardiac procedures and are also conveyed through information materials. In addition, the educational and cognitive behavioral elements should also support patients in the further course of health and disease management.

Concrete strategies:

- **Assessment of psychosocial status** (e.g., social support, quality of sleep, psychological comorbidities, distress due to physical discomfort, cognitive limitations, psychopharmacological treatment)
- **Reduction of anxiety** (e**.g.,** expectation anxiety, fear of death and complication fears through cognitive restructuring)
- **Psychoeducation** (e.g., elaborate subjective model of disease of the patients, explain the purpose of stress management as well as psychotherapy and psychopharmaceuticals, emphasize importance of adherence, activation of social support, develop realistic expectations regarding postoperative phase and intensive care unit), education about delirium
- **Development of treatment motivation** (elements of motivational interviewing, systemic questions on the solution-oriented approach [e.g., miracle question]; reframing, correction of dysfunctional disease assumptions, strengthening self-efficacy, taking into account the limits of autonomy and individual preferences of the patient. Furthermore, imagination exercises taking into account the personal interests and wishes of the patients, development of coping strategies [e.g., skills suitcases], highlighting the benefits of surgery compared to the initial situation, as well as stressing the regained control of heart disease (behavior-related result expectancy).

During the prehabilitation intervention, two psychological sessions of 50 minutes each are recommended. Topics “The Intervention – Newly gained freedom and life savior” and “The time after the procedure – I’m ready”.

Precautions:

Please do not make patients feel insecure, especially if treatment-related fears and fears about the future are discussed. It is recommended to focus on reducing anxiety and improving the quality of life after the procedure. In the case of a known depressive disorder, it is possible to ask about passive or active suicidal ideations. If there is a psychopharmacological medication, attention should be paid to delirogenic drugs (e.g., benzodiazepines). It is advised to be informed about possible dependencies. Furthermore, no antidepressants should be paused as part of the cardiac procedure (precautionary with weaning phenomena).

The content of this module is mainly based on the following references:

Rief W, Shedden-Mora MC, Laferton JAC, Auer C, Petrie KJ et al (2017). Preoperative optimization of patient expectations improves long-term outcome in heart surgery patients: Results of the randomized controlled PSY-HEART trial. BMC Medicine 15:4.; Sadlonova M, Nagel J, Becker S, Neumann S, Staab J, Meyer T, et al. Feasibility and preliminary efficacy of perioperative interventions in patients undergoing coronary artery bypass grafting: the randomized controlled I-COPE trial. Eur J Cardiothorac Surg 2022. doi:10.1093/ejcts/ezac041.; Tigges-Limmer, K. (2010). Wirksamkeit psychologischer Interventionen auf Genesung herzchirurgischer Patienten. https://www.db-thueringen.de/servlets/MCRFileNodeServlet/dbt_derivate_00021101/Tiggesimmer-/dissertation.pdf Zugriff 15.11.2022.; Tigges-Limmer, K., Rosendahl, J., Strauss, B. et al (2011). Psychologische Interventionen in der Herzchirurgie. Z Herz- Thorax- Gefäßchir 25 (Suppl 1), 29 (2011). https://doi.org/10.1007/s00398-011-0836-z.; Tigges-Limmer, K., Sitzer, M. und Gummert, J. (2021). Psychologische Akutinterventionen in der Herzchirurgie. Möglichkeiten und klinischer Nutzen. In: Deutsches Ärzteblatt Dtsch Arztebl Int 2021; 118: 339-45; DOI: 10.3238/arztebl.m2021.0116.

Module 5: Disease specific education

The motivation for treatment and adherence of the patients is a crucial prerequisite for the long-term success of a surgical or interventional cardiac procedure. Through targeted and individually adapted education, advice and training, knowledge, attitude, motivation and skills

of the patient can be improved in relation to heart disease and cardiovascular risk factors. Training content is made available in the prehabilitation centers in the form of video material.

The disease-specific trainings include the following topics:

- Help and psychological support in coping with the disease.
- Implementation of a healthy lifestyle (e.g., diet, exercise)
- Smoking cessation (e.g., behavioral therapy-based training programs for smokers)
- Dealing with stress and mindfulness
- Prevention, detection, and treatment of cardiovascular risk factors and high-risk diseases.
- Information about major cardiology or cardiac surgery procedures
- Recognition and behavior in acute disease-related complications in cardiac surgery or cardiology

The above-mentioned disease-specific training courses can be accessed at the following link: https://herzzentrum.umg.eu/precovery/patienten/. The duration of the videos varies from 5-20 minutes.

The content of this module is based on expert-interviews.

Module 6: Informative talks with relatives

The uncertainty before a planned cardiac procedure can lead to anxiety, withdrawal or restraint, as well as social isolation in affected patients. The cardiac diseases and their consequences not only affect the patients, but often involve a significant burden on the relatives. This can also lead to family conflicts and stressful situations.

Concrete strategies:

- 50 minutes, one conversation between psychologist and relatives at the beginning of the prehabilitation
- Focus on elucidation with regard to basic cardiological disease and cardiovascular risk factors, description of the prehabilitation process and the role of relatives; reduction of fears and time for open questions, realistic preparation for the planned cardiac procedure, emphasize the support role of the family and the form of support

Precautions:

Relatives should not be unsettled, especially when treatment-related fears and future fears are discussed. In the case of pronounced own fears of the relatives, it should be explicitly pointed out that they should not transmit their own fears to the patients. The focus should also be on reducing anxiety and improving the patient's quality of life after the procedure.

The content of this module is mainly based on the following references:

Tigges-Limmer, K. (2010). Wirksamkeit psychologischer Interventionen auf Genesung herzchirurgischer Patienten. https://www.db-thueringen.de/servlets/MCRFileNodeServlet/dbt_derivate_00021101/Tiggesimmer-/dissertation.pdf Zugriff 15.11.2022.; Tigges-Limmer, K., Sitzer, M. und Gummert, J. (2021). Psychologische Akutinterventionen in der Herzchirurgie. Möglichkeiten und klinischer Nutzen. In: Deutsches Ärzteblatt Dtsch Arztebl Int 2021; 118: 339-45; DOI: 10.3238/arztebl.m2021.0116.; Wilz, G., Meichsner, F. (2012). Involving family members of chronically ill patients in physician-patient communication. DOI: 10.1007/s00103-012-1532-1.; Gay, E., B., Pronovost, P., J., Bassett, R., D., Nelson, J., E. (2009).

The intensive care unit family meeting: Making it happen. In: Journal of Critical Care, vol. 24, Issue 4, December 2009, Pages 629.e1-629.e12.

Module 7: Special hygiene training/nutritional intervention

Nutrition

The focus of this module is on the preoperative detection of deficiency conditions in order to prepare patients as optimally as possible for the surgical procedure.

Concrete strategies:

- 1-2 appointments with a nutritional therapist during prehabilitation, 30 minutes each session
- Focus on compensation for missing nutrients (see blood count from recruitment center), detection/remediation of malnutrition and preoperative reduction of cardiovascular risk factors (e.g., optimization of blood sugar as well as blood fat levels).
- Even without deficiency, the following recommendations can be implemented during the preoperative period in addition to regular food intake: vitamine D supplementation and protein intake of 20-40 g per day (e.g., via whey protein).

Precautions**:**

The consumption of coffee, alcohol and sugar should be avoided or at least reduced during the preoperative period in order to counteract postoperative complications. By patients with severe renal insufficiency (glomerular filtration rate by 15 ml/min), you should be careful with protein substitution as it triggers uremia.

Special health training

The elucidation with regard to a targeted health concept should be strongly focused on the elderly patients and the clear therapeutic approach "to bring people to self-responsibility". All patients receive general information on the importance of personal care (e.g., dental hygiene after heart valve surgeries).

- Education on hygiene measures and their usefulness before and after the planned cardiac procedure (e.g., protection against urinary tract infection, pneumonia, bronchitis or gastrointestinal infection)
- Education on adequate dental hygiene after cardiac valve surgery
- Education regarding the need for endocarditis prophylaxis after heart valves procedure

A video is part in the disease-specific training courses and can be accessed at the following link: https://herzzentrum.umg.eu/precovery/patienten/.

The content of these modules is mainly based on the following references:

Cermak NM, Res PT, De Groot LC, Saris WH, Van Loon LJ. Protein supplementation augments the adaptive response of skeletal muscle to resistance type exercise training a meta analysis. Am J Clin Nutr 2012;96:1454-64.; Tang JE, Manolakos JJ, Kujbida GW, et al. Minimal whey protein with carbohydrate stimulates muscle protein synthesis following resistance exercise in trained young men. Appl Physiol Nutr Metab 2007;32: 1132-8.; Gabay C, Kushner I. Acute-phase proteins and other systemic responses to inflammation. N Engl J Med 1999;340:448–54.

3 Therapy units, weekly schedule & minimum requirements

The following table gives an insight into the therapy units with duration and frequency per week.

Table 2. Therapy units with duration and frequency per week.

Therapy unit	Duration	Week 1	Week 2
Aerobic endurance training	30 min.	3x	3x
Dynamic strength training + flexibility training	30 min.	2x	2x
Coordination training (group gymnastics)	30 min.	3x	3x
Respiratory therapy (group)	30 min.	3x	3x
Breathing therapy (self-training)	Max. 30 min.	5x	5x
Occupational therapy (single)	30 min.	2x	2x
Cognition training	30 min.	3x	3x
Progressive muscle relaxation (PMR)*	30 min.	3x	3x
Psychology (individual)	60 min	1x	1x
Massage*	25 min.	2x	2x
Visite, Laboratory & Diagnostics	15-20 min.	2x + if required	2x + if required
Family Talks	25 min.	1x	1x
Social Service (individual)	30-60 min.	if necessary,	if necessary,
Diet counselling (individual)	30 min.	1x	if necessary,
Hygiene training	15-20 min.	2x	2x
Sickness-specific education	60 min.	2x	2x

* Not described in treatment manual. Optionally feasible in prehabilitation center, provided that PMR/massage is initiated for patients and is offered in regular care.

Abbreviations. min. = minute.

The structure of an exemplary weekly schedule can be found in Table 3.

Table 3. Exemplary weekly schedule of prehabilitation.

	**Monday**	**Tuesday**	**Wednesday**	**Thursday**	**Friday**
**07:30**	Laboratory	Diagnostics	Laboratory	Diagnostics	Laboratory
**08:00**	Breakfast				
**08:30**					
**09:00**	Exercise Therapy	Occupational therapy		Occupational therapy	Social Counselling
**09:30**					
**10:00**	Breathing Therapy		Breathing Therapy		Breathing Therapy
**10:30**	Visit	Visit	Visit	Visit	Visit
**11:00**		Exercise Therapy	Exercise Therapy		
**11:30**	Cognitive Therapy			Cognitive Therapy	
**12:00-13:30**	Lunch				
**14:00**					
**14:30**	Massage	Cognitive Therapy		Exercise Therapy	Exercise Therapy
**15:00**			Occupational therapy		
**15:30**					
**16:00**	Health education	Conversations with relatives	Psychosocial intervention	Psychosocial intervention	Health education
**16:30**					
**17:00**	Breathing Therapy	Breathing Therapy	Breathing Therapy	Breathing Therapy	Breathing Therapy
**17:30**	Dinner				

Table 4 summarizes the overview of the minimum therapy requirements during the prehabilitation program “Karl-Heinz”.

Table 4. Overview of the minimum of therapy requirements during prehabilitation program “Karl-Heinz”.

Form of therapy	Minimum of requirements
(1) Sports and exercise therapy	Scope: 1 active unit per day (5x per week) of 30 minutes each.<br>Content: endurance, strength/flexibility, coordination training
(2) Breathing therapy	Scope: 3 times per week of 30 minutes each.<br>Content: Group setting with daily self-training
(3) Occupational therapy	Scope: 2 times per week of 30 minutes each.<br>Content: see exercise therapy plus tips for the sustainability of prehabilitation
(4) Cognitive training	Scope: 2 times per week of 30 minutes each.<br>Content: attention and concentration training
(5) Psychosocial intervention	Scope: 2 times during prehabilitation period<br>Content: Delirium education, preparation for surgery inclusive procedures in the hospital and PMR
(6) Disease specific education	Scope: 3 units in the prehabilitation period, video training if necessary.<br>Content: sleep, nutrition, exercise, risk factors, mindfulness, social counseling.
(7) Informative talks with relatives	Scope: 1 time during the prehabilitation period<br>Content: see treatment manual in the addional file
