# Supplementary material for: Prehabilitation in older patients prior to elective cardiac procedures (PRECOVERY): study protocol of a multicenter randomized controlled trial
Source: Trials. 2023 Aug 15;24:533. doi: 10.1186/s13063-023-07511-w (PMC10426165; doi:10.1186/s13063-023-07511-w)
Supplement: Supplementary file 4 — Additional file file 4. [file 13063_2023_7511_MOESM4_ESM.pdf]

Gemeinsamer
Bundesausschuss
Innovationsausschuss

**Geschäftsstelle**

**Besuchsadresse:**
Gutenbergstraße 13
10587 Berlin

**Ansprechpartner/in:**
Dr. Annelie Brendel

**E-Mail:**
Annelie.brendel@if.g-ba.de

**Internet:**
www.if.g-ba.de

**Unser Zeichen:**
01NVF21109

**Datum:**
23. Januar 2023

**Innovationsausschuss beim G-BA**, Postfach 12 06 06, 10596 Berlin

Prof. Dr. Christine von Arnim
Universitätsmedizin Göttingen
Abteilung für Geriatrie
Robert-Koch-Straße 40
37073 Göttingen

**Confirmation government funding and assessment corresponding to a peer-review**

Dear Professor von Arnim,

we hereby confirm that the project „PRECOVERY – Prehabilitation „Karl-Heinz“ mit Schwerpunkt auf cardiale und kognitive Funktionen vor Eingriffen am Herzen: eine Analyse des Gesundheitszustands“ is funded by the German Innovation Fund with the funding number: 01NVF21109.

Projects funded by the German Innovation Fund run through an assessment procedure that correspond to a peer-review.

Details of the assessment procedere are described in § 9 of the Code of procedure of the council of the German Innovation Fund: Verfahrensordnung IA (g-ba.de).

The application for „ PRECOVERY“ was assessed by an council of experts and finally (under consideration of the results of the experts) of the council of the German Innovation Fund (existing of 12 members).

The council of the German Innovation Fund is located at the Federal Joint Committee (G-BA)[1]. The Federal Joint Committee is the highest decision-making body of the joint self-government of

[1] in German: Innovationsausschuss beim Gemeinsamen Bundesausschuss.

physicians, dentists, hospitals and health insurance funds in Germany. It issues directives for the benefit catalogue of the statutory health insurance funds (GKV) for more than 73 million insured persons and thus specifies which services in medical care reimbursed by the GKV.

The documentation of the PRECOVERY project is accessible on the website of the funding body: PRECOVERY – Prehabilitation „Karl-Heinz“ mit Schwerpunkt auf cardiale und kognitive Funktionen vor Eingriffen am Herzen: eine Analyse des Gesundheitszustands - G-BA Innovationsfonds.

Please contact us if additional questions arise.

Sincerely yours,

Brendel

Dr. Annelie Brendel
