# Supplementary material for: Prehabilitation in older patients prior to elective cardiac procedures (PRECOVERY): study protocol of a multicenter randomized controlled trial
Source: Trials. 2023 Aug 15;24:533. doi: 10.1186/s13063-023-07511-w (PMC10426165; doi:10.1186/s13063-023-07511-w)
Supplement: Supplementary file 5 — Additional file file 5. [file 13063_2023_7511_MOESM5_ESM.pdf]

UNIVERSITÄTSMEDIZIN GÖTTINGEN UMG

Ethik-Kommission der Universitätsmedizin Göttingen, Von-Siebold-Straße 3, 37075 Göttingen

Ethik-Kommission der
Universitätsmedizin Göttingen
Vorsitzender: Prof. Dr. Jürgen Brockmöller
Referentin
Regierungsrätin Doris Wettschereck
0551 / 39-68644 **Telefon**

Von-Siebold-Straße 3, 37075 Göttingen Adresse
0551 / 39-61261 **Telefon**
0551 / 39-69536 **Fax**
ethik@med.uni-goettingen.de **E-Mail**
www.ethikkommission.med.uni-goettingen.de

Frau
Prof. Dr. med. Christine von Arnim
Klinik für Geriatrie
Robert-Koch-Straße 40
37075 Göttingen

per christine.arnim@med.uni-goettingen.de

25.01.2023 br - fr Datum

**Application no.**:	**20/12/22** (please refer to this number in all correspondence)
**Study title**:	Prehabilitation “Karl-Heinz” mit Schwerpunkt auf cardiale und kognitive Funktionen vor Eingriffen am Herzen: eine Analyse des Gesundheitszustands<br><br>Study title in English: Prehabilitation "Karl-Heinz" focusing on cardiac and cognitive functions prior to cardiac interventions: a health status analysis
**Submitter:**	Prof. Dr. med. Christine von Arnim, Dr. med. Stephanie Heinemann, Dr. med. Carolin Steinmetz, Klinik für Geriatrie, Dr. med. Monika Sadlonova, Prof. Dr. med. Ingo Kutschka, Klinik für Thorax-, Herz- und Gefäßchirurgie, Prof. Dr. med. Gerd Hasenfuß, Klinik für Kardiologie und Pneumologie, Prof. Dr. med. Eva Hummers, Institut für Allgemeinmedizin, UMG

The following documents were submitted for evaluation:

- Cover letter from 13.01.2023 (E-Mail)
- Cover letter to Amendment from 10.01.2023
- PRECOVERY_Studienprotokoll_V1.1_20230110, with changes marked
- Gesamtdokument_Anlagen_V1.1_20230110G_komprimiert, with changes marked

Dear Prof. Dr. von Armin, Dear Sir / Madam,

We hereby acknowledge that the ethics committee of the Medical Faculty of the University of Göttingen has taken note of the Amendment. The members of our Ethics-committee did not have any ethical or legal objections against this amendment.

We would like to inform you that irrespective of this positive vote allowing to start with the study it is your responsibility to control at every step of the investigations the ethically and legally correct conduct of the study. Any changes in the study protocol or termination of the study have to be reported to the Ethics committee. When the study is finished please be so kind to send us a brief report about the study including any severe adverse events and the results from the study; alternatively a scientific publication about the study would fulfill the same purpose.

We wish you a good success with your interesting research proposal.

Sincerely yours

Prof. Dr. Jürgen Brockmöller
Professor of Clinical Pharmacology
Chairmen of the Ethics committee
